# PubChem chemical structure standardization

**DOI:** 10.1186/s13321-018-0293-8

**Published:** 2018-08-10

**Authors:** Volker D. Hähnke, Sunghwan Kim, Evan E. Bolton

**Affiliations:** 1 National Center for Biotechnology Information, National Library of Medicine, National Institutes of Health, Department of Health and Human Services, 8600 Rockville Pike, Bethesda, MD 20894 USA; 2 Present Address: European Patent Office, Patentlaan 2, 2288 EE Rijswijk, The Netherlands

**Keywords:** PubChem, Standardization, InChI, Tautomerism, Aromaticity, Kekulization

## Abstract

**Background:**

PubChem is a chemical information repository, consisting of three primary databases: Substance, Compound, and BioAssay. When individual data contributors submit chemical substance descriptions to Substance, the unique chemical structures are extracted and stored into Compound through an automated process called structure standardization. The present study describes the PubChem standardization approaches and analyzes them for their success rates, reasons that cause structures to be rejected, and modifications applied to structures during the standardization process. Furthermore, the PubChem standardization is compared to the structure normalization of the IUPAC International Chemical Identifier (InChI) software, as manifested by conversion of the InChI back into a chemical structure.

**Results:**

The observed rejection rate for substances processed by PubChem standardization was 0.36%, which is predominantly attributed to structures with invalid atom valences that cannot be readily corrected without additional information from contributors. Of all structures that pass standardization, 44% are modified in the process, reducing the count of unique structures from 53,574,724 in substance to 45,808,881 in compound as identified by de-aromatized canonical isomeric SMILES. Even though the processing time is very low on average (only 0.4% of structures have individual standardization time above 0.1 s), total standardization time is completely dominated by edge cases: 90% of the time to standardize all structures in PubChem substance is spent on the 2.05% of structures with the highest individual standardization time. It is worth noting that 60% of the structures obtained from PubChem structure standardization are not identical to the chemical structure resulting from the InChI (primarily due to preferences for a different tautomeric form).

**Conclusions:**

Standardization of chemical structures is complicated by the diversity of chemical information and their representations approaches. The PubChem standardization is an effective and efficient tool to account for molecular diversity and to eliminate invalid/incomplete structures. Further development will concentrate on improved tautomer consideration and an expanded stereocenter definition. Modifications are difficult to thoroughly validate, with slight changes often affecting many thousands of structures and various edge cases. The PubChem structure standardization service is accessible as a public resource (https://pubchem.ncbi.nlm.nih.gov/standardize), and via programmatic interfaces.
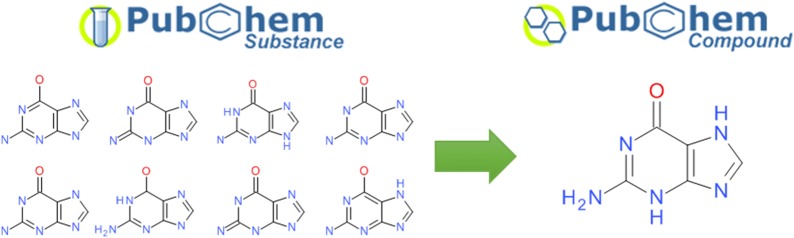

**Electronic supplementary material:**

The online version of this article (10.1186/s13321-018-0293-8) contains supplementary material, which is available to authorized users.

## Background

**Fig. 1:**
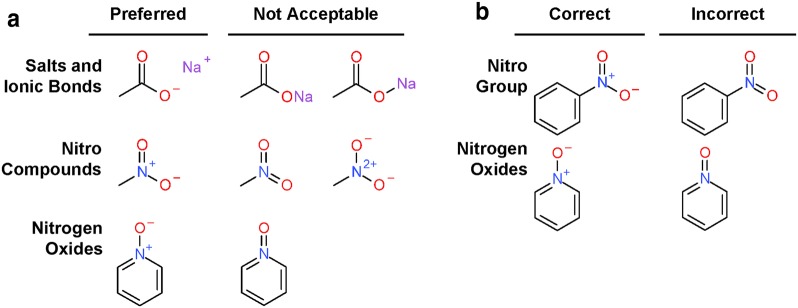
Exemplary drawings conventions for functional groups. **a** Examples taken from the IUPAC graphical representation standards for chemical structure diagrams concerning ionic bonds and salts and nitrogen compounds [7]. **b** Examples taken from the FDA substance registration system standard operating procedure substance definition manual. For nitro group and nitrogen oxides, both conventions agree on the preferred representation [8]

Chemical information has co-evolved with cheminformatics over the past 40 or so years [1–3]. Whereas cheminformatics focuses on development and application of property prediction models for atoms and molecules [4–6], the primary tasks of chemical information are the accurate representation, registration, and retrieval of chemical structures in computer systems. The lack of universally adopted standards for chemical structure representation in chemical structure collections is notable. The International Union of Pure and Applied Chemistry (IUPAC) released guidelines in 2007 for the graphical representation of chemical structure diagrams, defining how structures should be depicted for unambiguous human interpretation [7]. These contain specifications and recommendations for two-dimensional (2-D) molecular structure diagrams considering bond angles and lengths, atom label font, line widths, and the layout of ring systems. Only for very few cases do they contain specifications for the actual configuration of atoms and bonds, with respect to location of charges and bond orders. Furthermore, there is a notable lack of consideration for machine interpretation, for example, by allowing implied stereo in saccharide rings (please see examples in Fig. 1). The same is true for the “US Food and Drug Administration (FDA) Substance Registration System Standard Operating Procedure Substance Definition Manual” (accessed March 2013) [8] (the latter, in earlier versions, was explicitly referred to as the ‘Structure Drawing Guide’). With a lack of globally recognized and enforced standards and a large pre-existing corpus of chemical structures from various data sources, the representation of structures or structural elements is highly influenced by multiple factors. These include chemists’ personal preferences, organization-based conventions, history, and so called ‘RoboChemistry’ (computer algorithms providing automated clean-up by adapting structure layout, functional group representation, aromaticity annotation and tautomeric states to diverging standards, potentially leading to the corruption and deterioration of entire structure collections).

Several machine-readable molecule representations have been developed. Among the most popular are line notations [9–17], systematic IUPAC names [18–20], connection table files, and reaction data files [21–24]. The level of detail in these representations varies, especially with respect to the specification of hydrogen atoms and the configuration of stereocenters. Conversion between different structure representations is prone to information loss and errors [25, 26]. The perception of structures from three-dimensional (3-D) atom coordinates is an additional source for structural errors [27–30]. Erroneous (interpretation of) structures are a major problem, as it has been shown that even small errors in structure representations can lead to significant loss of predictive ability of computer models [31], affecting downstream computation in cheminformatics.

**Fig. 2:**
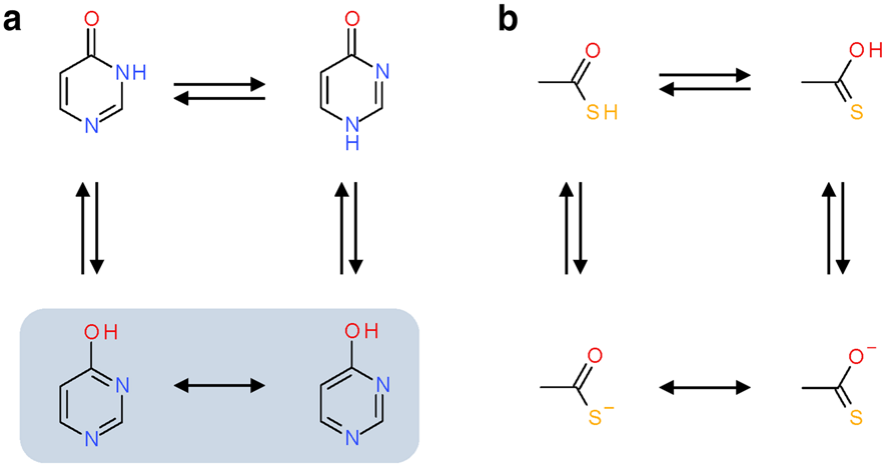
Natural effects contributing to molecular diversity. Implicit hydrogen atoms on carbon atoms are not shown. **a** Three tautomeric variants of pyrimidin-4-one. The bottom two structures are different Kekulé representations of the same tautomer. **b** Thioacetic acid as an example for tautomerism (top, in left–right direction), ionization (left and right, in top–bottom direction) and mesomerism (bottom, in left–right direction). Redrawn with permission from Sayle 2010 [32]

**Fig. 3:**
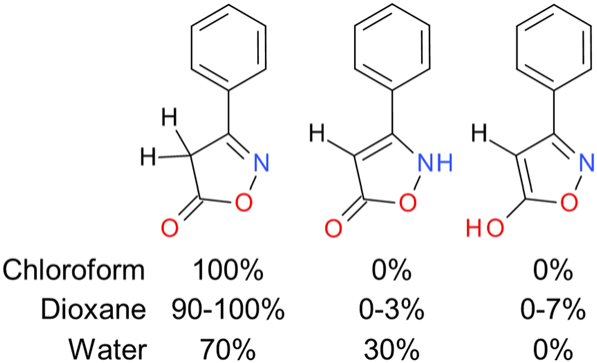
Effects of solvent on tautomeric preference for simple heterocycles. Listed are percentages of three tautomeric variants of the same structure in different solvents [36]

**Fig. 4:**
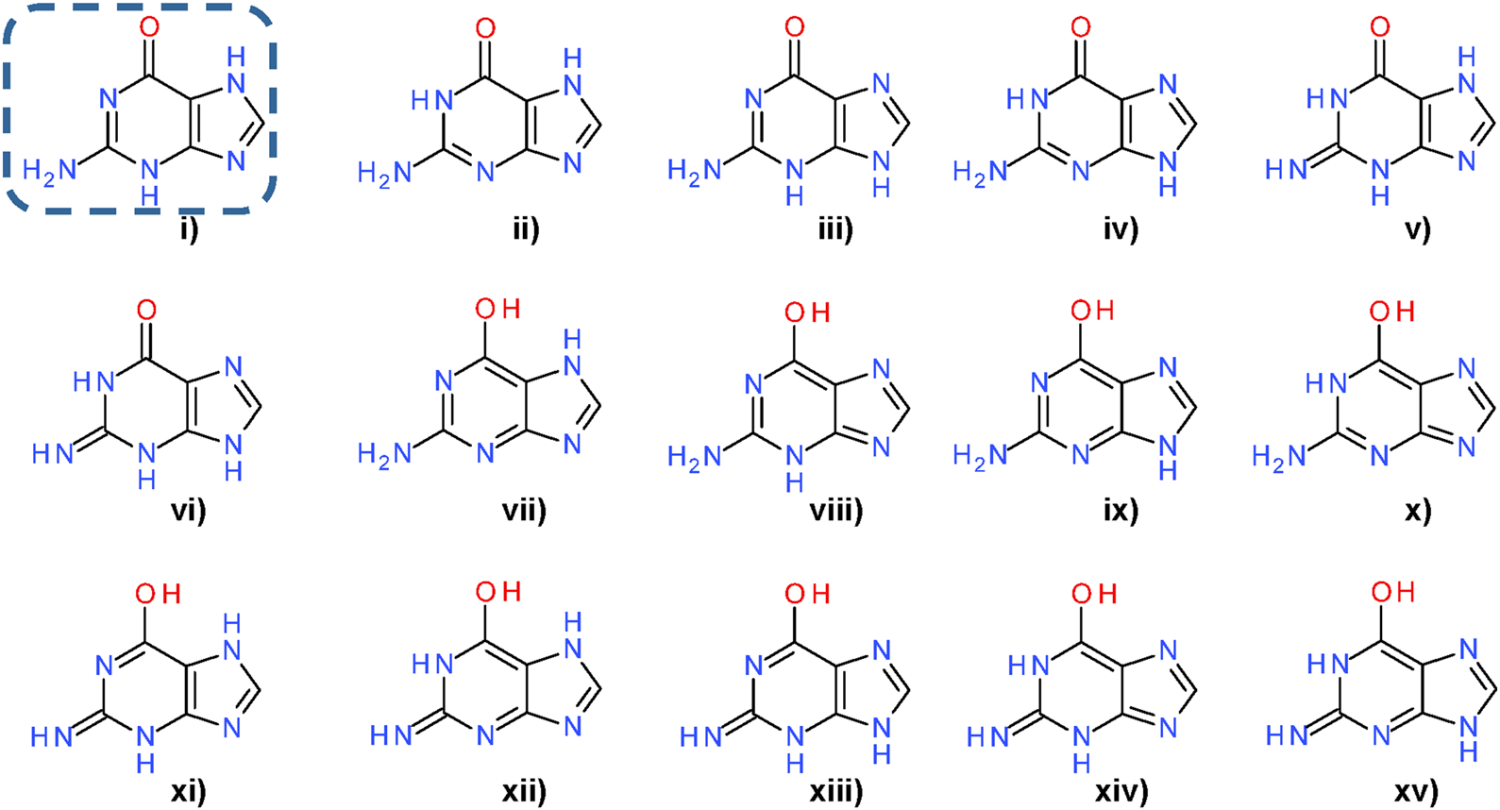
Tautomers of Guanine. Tautomers were generated by the approach described in the “Methods” section (under *Standardize Valence Bond Form*) in the indicated order. The dashed frame highlights the variant chosen from the ensemble as the canonical tautomer

Tautomerism, mesomerism and alternate ionization states contribute to the number of possible valid, non-identical representations of the same structure, which often exist in equilibrium, as illustrated in Fig. 2 [32]. Tautomer standardization and prediction algorithms can yield diverging results because of different enumeration strategies, diverging opinions on energy barriers between representations, or assumptions about external factors such as solvent, temperature, and pH, which can strongly influence the dominating tautomeric species (Fig. 3) [33–36]. The choice of representative tautomers has consequences in computed properties such as the assignment of hydrogen bond acceptor and donor functionalities in the definition of potential pharmacophoric features [37]. It was shown that tautomerism and choice of the predominant variant heavily impact computed compound similarity, predicted activity and other properties [38–45]. Diverging tautomer representations can also influence the recognition of features in structure-based chemical ontologies [46, 47]. This is not a minor problem: rates of affected structures in databases have been reported between 0.5% [48], 26% [49], 30% [50], and > 67% [38]. Several methods for the enumeration of tautomers have been published [38, 39, 48, 50]. While they enable access to various tautomers of a structure, they create a new problem: preferred tautomeric forms must be identified, and, if desired, one needs to be chosen as the canonic representative. The problem is illustrated in Fig. 4 using tautomers of guanine as an example. Selection criteria reported in the literature are based on predicted stability [50, 51], or count-based scoring functions [38, 40]. For chemical substance registration purposes, the generation of an arbitrary canonical tautomer may be sufficient [48, 52, 53] for uniqueness, even though ramifications for structure and substructure searches can be severe, if downstream search methods employed do not account for tautomer ambiguities [54]. On the contrary, appropriate tautomers should be selected for any applications that involve prediction of physicochemical properties of compounds.

**Fig. 5:**
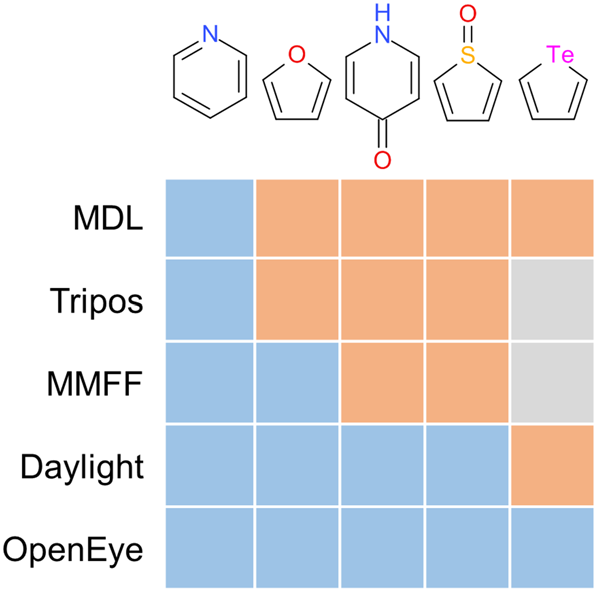
Comparison of five aromaticity perception models. Structure classification as aromatic is indicated by color (blue: aromatic; orange: not aromatic; grey: not available). Aromaticity was perceived in every structure using the function OEAssignAromaticFlags in the OpenEye OEChem C++ toolkit with the aromaticity models OEAroModelMDL (MDL), OEAroModelTripos (Tripos), OEAroModelMMFF (MMFF), OEAroModelDaylight (Daylight) and OEAroModelOpenEye (OpenEye). If at least one atom or bond in a structure was identified as aromatic, the whole structure was classified as aromatic. Atomic element Te is not available in the MMFF and Tripos aromaticity models. Redrawn with permission from the OpenEye Scientific Software Inc. OEChem C++ toolkit documentation [62]

The ‘aromaticity’ of a molecule can be considered a basic, yet underappreciated, ‘concept’ by users of chemical information. Aromaticity can be defined in a multitude of ways, based on various criteria including chemical behavior, energetic properties, magnetic effects, and structural features [55–59]. In the chemistry classroom, aromaticity is often taught as a binary property with a definition based on Hückel’s rule [60, 61], which is also implemented in aromaticity perception algorithms commonly used in cheminformatics. Unfortunately, implementations differ in the treatment of heteroatoms, exocyclic double bonds, considered ring size (such as in the case of the so-called ‘MDL aromaticity model’ used to assign the MACCS keys fingerprint where only alternating single/double bonds in a six-membered ring can be considered aromatic), and the handling of charged atoms, resulting in different aromaticity detection results as illustrated in Fig. 5 [62]. This impedes the exchange of structures and data and impairs the reproducibility of results, as the same structure could be represented with diverging aromaticity annotations originating from different perception models. Furthermore, these models may differ between implementations, especially in so called “corner cases” often involving various atom-types, potentially contributing to a change in structural identity to a related isomer with a significant energy barrier for interconversion.

Conversely, ‘aromatic’ moieties in structures can be represented in Kekulé form using alternating single- and double bonds [63, 64]. Several algorithms for the enumeration of Kekulé structures of conjugated systems have been reported in the literature [65–70]. Kekulé forms of a molecule (as opposed to the aromatic representation) may be necessary when computing descriptors or properties about a chemical structure or to remove ambiguity in aromaticity interpretation. Yet, methods attempting to generate a single representative Kekulé form (a process referred to as ‘kekulization’) are either heuristics (i.e., may not find a Kekulé representation even though it exists) or remain arbitrary (i.e., non-canonical) in the resulting structure [71, 72]. To the best of our knowledge, no method has been described that is dedicated to the generation of a representative canonical Kekulé form. This issue compounds the lack of a standard definition of aromaticity, because aromaticity is typically perceived from a Kekulé structure. On the other hand, given a structure with ‘aromatic’ (instead of single and double) bonds, the underlying (canonical) Kekulé structure is not obvious. Consequently, kekulization approaches should be able to deal with the various existing aromaticity definitions and compensate for their intrinsic differences, without generating cases where conjugation is broken (e.g., *–C=C=C–* or * = C–C–C= * instead of *–C=C–C= *) or where a different count of double bonds occurs due to differences in handling exo-cyclic heteroatoms. Lastly, aromaticity approaches should be coupled closely with tautomer handling approaches, as choice of tautomeric form may directly affect aromaticity, depending on the aromaticity model employed.

Chemical structure standardization is of utmost importance to compensate for the diverse (and potentially ambiguous) nature of chemical structure representation and interpretation, while identifying and correcting (or rejecting) erroneous structures, to ensure proper interpretation of chemical content by a given data system. Yet, guidelines or performance measures for this purpose remain scarce [53, 73, 74]. With increasing size and popularity of public chemical information resources this issue becomes even more important as the ready ability to download, normalize, and share millions of chemical structures increases the potential for rapid and broad spread of errors [75–77]. Once erroneous structures are shared, errors in these copies may not be easily recognized or corrected, especially if the chemical structure is deemed valid and the original data content provenance is lost. This is not a minor problem, as the percentage of affected erroneous structures has been estimated to be between 0.1 and 8% [31, 78–80].

**Fig. 6:**
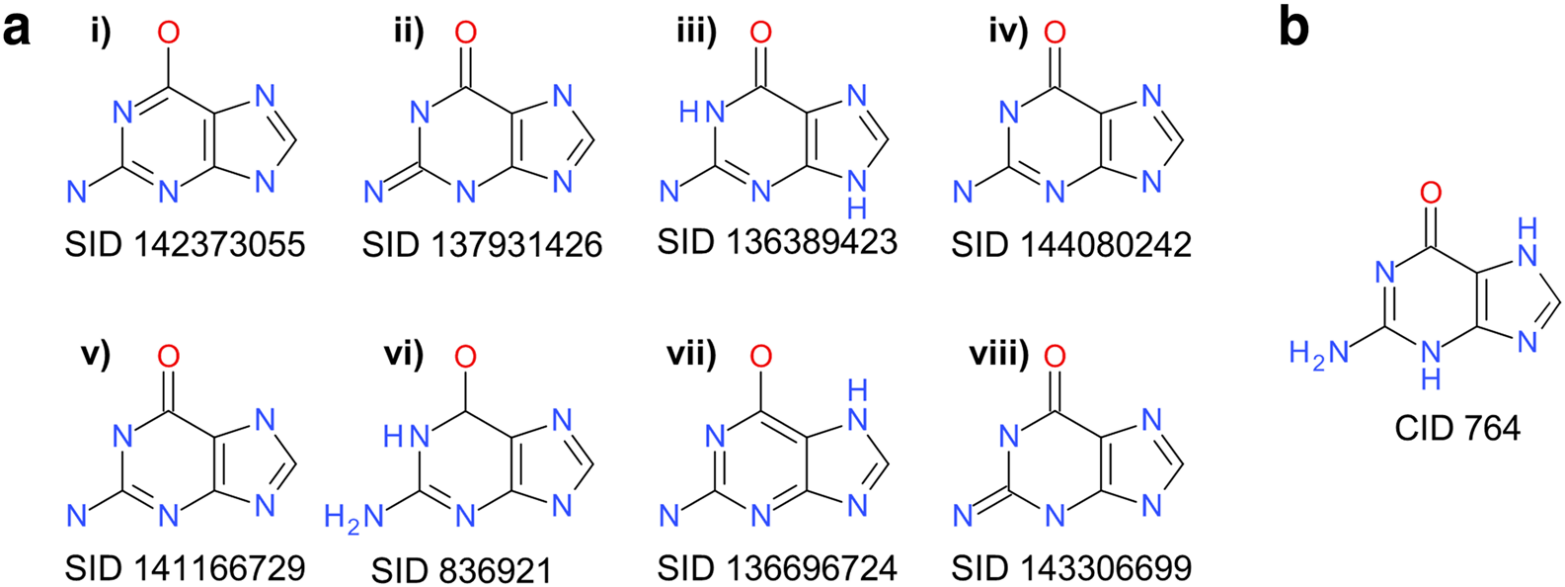
Substance to Compound relationship for guanine. In total, 153 entries in Substance are standardized to and mapped to the structure for CID 764. **a** Eight representative SIDs with non-identical structures that get standardized to guanine. No explicit hydrogen atoms were provided for (i), (ii), (iv), (v) and (viii). Hydrogen atoms are depicted as deposited for (iii), (vi) and (vii). **b** Standardized structure of Guanine (CID 764)

PubChem [81–83] is a public repository for information on chemical substances and their biological activities. It contains more than 237 million deposited chemical substances and 94 million unique structures as of December 2017. It is located at the US National Center for Biotechnology Information (NCBI), part of the US National Library of Medicine (NLM), an institute of the US National Institutes of Health (NIH). PubChem first became available in 2004 as a part of the Molecular Libraries and Imaging (MLI) component of the NIH Roadmap for Medical Research Initiative. With millions of unique users per month, thousands of citations (e.g., search PubMed [84, 85] for the term ‘pubchem’ in title or abstract), and a constantly evolving collection of content from a diverse set of hundreds of data contributors, PubChem deals with the aforementioned chemical structure normalization issues on a very large scale. To provide consistency and a highly visible provenance trail, structural information is stored in two separate databases: Substance and Compound. Substance contains versioned sample descriptions from individual contributors without any normalization processing (basically, as provided and interpreted). The Compound database is derived from Substance through automated structure standardization protocols that verify whether structures are chemically sensible (i.e., rooted in physical reality), recognize equivalent chemicals between depositors, and generate a preferred chemical representation. This allows for aggregating information between contributors by mapping substances (and their associated information) to the corresponding standardized compound record. An example of the resulting ‘many-to-one’ relationship arising from the standardization process is shown in Fig. 6. The standardized structures in Compound are then used as the basis for further computation of basic chemical properties and 3-D conformations [86–88].

**Fig. 7:**
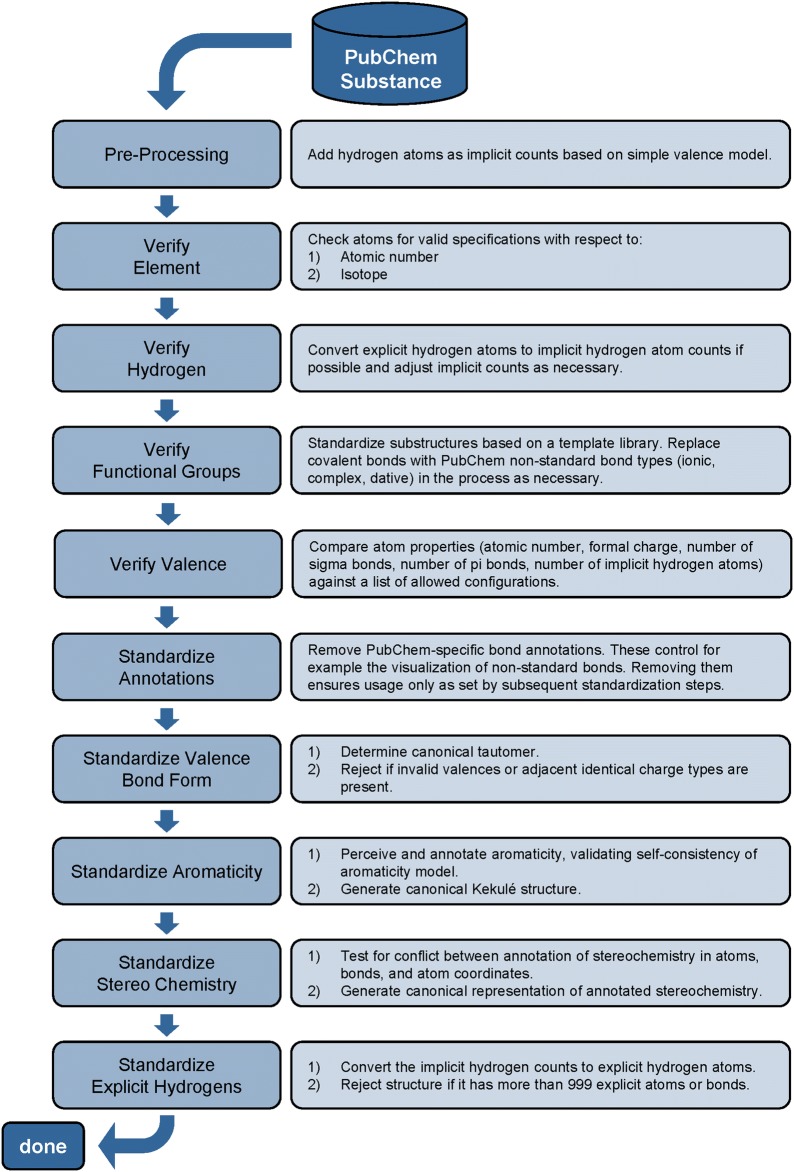
PubChem structure standardization protocols. For detailed descriptions of each step, see the “Methods” section

The PubChem structure standardization protocols are built on top of the OpenEye Scientific Software, Inc. C++ toolkits [89–92]. As outlined in Fig. 7 and described in the “Methods” section, the standardization process consists of two major phases (structure verification and structure normalization), which can be further divided into nine steps.

1. *Verify element*, which evaluates the validity of specified element and isotopic information.
2. *Verify hydrogen*, which performs adjustments to implicit hydrogen counts, as necessary.
3. *Verify functional groups*, which puts diverse functional group representations into a preferred form.
4. *Verify valence*, which evaluates connectivity and charge information per atom using a dictionary of allowed valences.
5. *Standardize annotations*, which removes perceived PubChem-specific bond type annotations.
6. *Standardize valence bond form*, which generates a canonical tautomer representation of the structure.
7. *Standardize aromaticity*, which determines a canonical Kekulé structure.
8. *Standardize stereochemistry*, which evaluates available information about stereocenters and attempts a canonical configuration.
9. *Standardize explicit hydrogens*, which converts implicit hydrogen counts to explicit hydrogen atoms in the molecular graph.

The present study describes each of these steps and presents examples for success as well as failure of the employed method. This study provides a global view of structures deposited in PubChem by analyzing structural redundancy before and after standardization. For this purpose, we compare the frequency of unique non-standardized structures and their corresponding standardized counterparts. The results are compared to those obtained from the normalization procedure performed in the generation of IUPAC International Chemical Identifiers (InChIs) [11–13] used elsewhere for compound registration [93], the reduction of database redundancy [94], and chemical data linking approaches.

## Results and discussion

### Standardization success rates

**Fig. 8:**
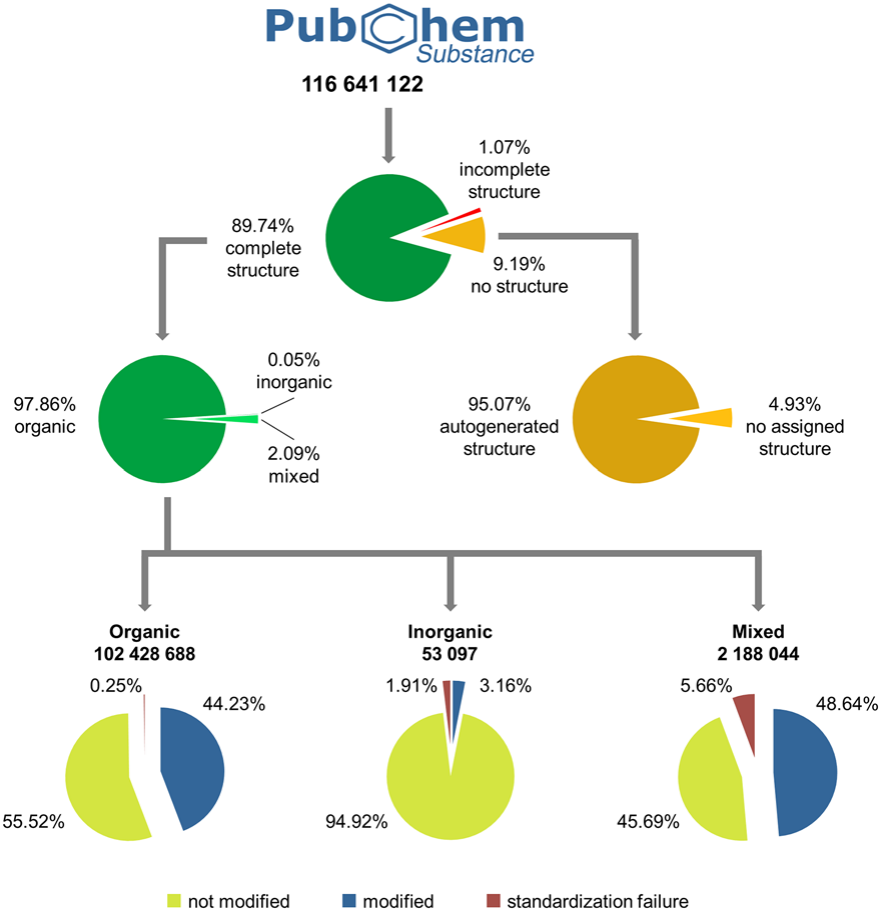
Standardization statistics. The version of PubChem Substance used in this study contains 116,641,122 deposited substances. Almost 90% of those entries contain fully specified structures. The majority of these are organic. The average standardization success rate is 99.64% with 44.43% of successfully standardizing structures getting modified in the process

Success and modification rates during standardization are presented in Fig. 8. The version of PubChem Substance used in this study contained 116,641,122 entries (from January 2013). Not all substances had fully defined structures. A total of 1,246,584 records in Substance (1.1%) contain chemical structures that have at least one arbitrarily defined atom (‘pseudo’-atom). In 10,724,749 cases (9.2%) no structure was deposited, and of these, 95.1% had a structure assigned (‘auto-generated’) using a chemical name (please note that this is not performed by default and it enables structure-less resources with chemical information to be integrated with PubChem). When no chemical structure is provided for a chemical substance, three different strategies are used for automated structure assignment by chemical name: (1) if the deposited substance contains a direct reference to an existing CID (e.g., “CID2244”), the corresponding structure is used; (2) if a chemical name is an annotated MeSH [95] synonym (e.g., “aspirin”), the structure assigned by PubChem to that name is used; and (3) name-to-structure conversion is performed using the OpenEye Lexichem Toolkit [92] (e.g., “1,2-dichloroethane”). If a non-conflicting chemical structure can be assigned by one of these three approaches (applied in the order mentioned), it is used as the chemical structure for the substance during standardization processing. Only 4.9% of the entries with no deposited structure have no structural information associated (a total of 528,484 substances). Entries with no structural information, auto-generated structures, and incompletely specified structures were not considered in the analysis of standardization protocols. The remaining 104,669,789 structures (89.7% of substance records) were subject to PubChem’s standardization approach and reported herein. Standardization was successful in 99.6% of all processed cases with a success rate of 99.8% for organic structures (only containing the ten organic elements: H, C, N, O, F, P, S, Cl, Br, and I), 98.1% for inorganic structures (only containing non-organic elements) and 94.3% for mixed structures (containing both organic and inorganic atoms). Only 0.4% of all cases (organic: 0.3%; inorganic: 1.9%; mixed: 5.7%) were rejected.

Table 1 lists the absolute rates of standardization failures for each step and each of the classes organic, inorganic and mixed. Most standardization failures are caused by invalid specifications of atomic information in the structure verification phase of standardization (i.e., the *Verify Element* and *Verify Valence* steps) (97.2% of all 376,355 rejected substances). A total of 141 substances are excluded from further processing because of an invalid combination of element and isotopic specification, and 365,485 substances fail the verification of atomic valences using an internal valence knowledgebase [provided as supporting information in Additional file 1 (see the “Methods” section)]. A typical example for the first case is “^4^Th” (thorium isotope with atomic mass 4 Da; SID 137288627). The known thorium isotopes have masses between 208 and 238 [96]; isotope ^232^Th has natural abundance of 100% [97]. The specified atomic mass of 4 Da is not among the known isotopes, consequently the atom is rejected and the substance fails standardization. Inspecting the original SDF file associated with this particular SID suggests an explanation for this unusual isotope: Using SDF format, isotopic information can be specified in two ways: (1) as part of the atom block as a delta value (i.e., a difference) to the most abundant isotope; and (2) in the properties block using the prefix ‘M ISO’ as an absolute value if it differs from the isotope that has highest natural abundance [21]. In the case of SID 137288627, method (2) was used with a value that would be appropriate for method (1), referring to ^236^Th. In total, 44 of the standardization failures in *Verify Element* are such mono-atomic substances. While fixable in this specific case, a generalized rule (which may do more harm than good) does not exist to correct this issue and the substance structure is rejected (i.e., not assigned a CID) as being invalid.

**Table 1:** Standardization rejection rates

	Organic	Inorganic	Mixed	Total
Number of substances	102,428,688	53,097	2,188,004	104,669,789
Verify element	107	14	20	141
Verify hydrogens	–	–	–	–
Verify functional groups	–	–	–	–
Verify valence	242,615	836	122,034	365,485
Standardize annotations	–	–	–	
Standardize valence bond	8245	165	1833	10,243
Standardize aromaticity	–	–	–	
Standardize stereochemistry	–	–	–	
Standardize explicit hydrogens	458	–	28	486
Rejected substances	251,425	1015	123,915	376,355
Rejection rate	0.25%	1.91%	5.66%	0.36%
Successfully standardized substances	102,177,263	52,082	2,064,089	104,293,434
Success rate	99.75%	98.09%	94.34%	99.64%

Listed in the table is the absolute number of rejected substances for every step of the PubChem standardization protocol and the total as well as the overall standardization rejection rate for every structure class (organic, inorganic, mixed) as applied to the PubChem Substance database

**Fig. 9:**
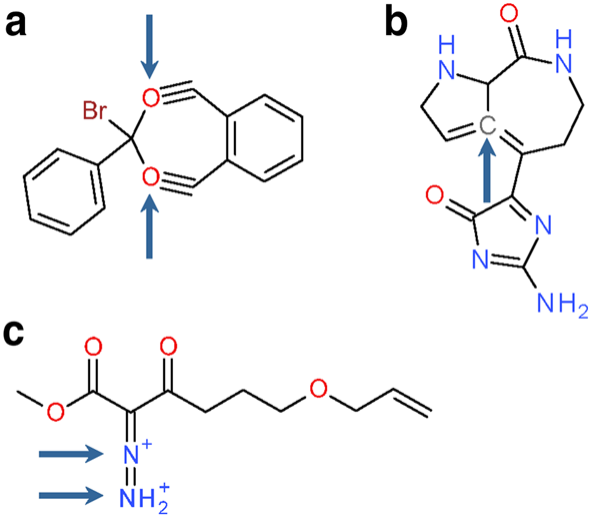
Standardization failure examples. Each structure is shown as it enters the respective standardization step, including modifications from previous steps. **a** SID 479450 contains two tetra-valent oxygen atoms (indicated by arrows) and fails the verification of atomic valences. **b** SID 8021026 contains a penta-valent carbon atom (indicated by arrow) and fails the verification of atomic valences as well. **c** SID 235635 contains two adjacent nitrogen atoms that both have a positive charge (indicated by arrows). A post-processing evaluation in the determination of a canonical tautomer lets structures fail if they have neighboring charges of the same type

An example of invalid valences is shown in Fig. 9a. SID 479450 contains two oxygen atoms that engage each in two σ and two π bonds, resulting in an oxygen valence of 4. The PubChem valence list does not allow tetra-valent oxygen, so the structure is rejected in the *Verify Valence* step. The original author of the depiction may have meant to imply a specialized interaction but it is not completely clear what was meant. Another example for a prominent valence violation is shown in Fig. 9b: SID 8021026 contains a penta-valent carbon atom. This may represent a simple mistake or a bizarre aromaticity Kekulization algorithm error (usually where an algorithm goes bad or where the molecule was aromatized by one algorithm but then Kekulized by another). As such, it is not clear what the original intent was without additional information and the structure is rejected. In total, 72,743 substances failing this step contain such pentavalent carbon atoms (many being likely aromaticity Kekulization errors by algorithms where an extra double bond is added, corrupting the molecule through the loss of a hydrogen molecule) (it is worth noting, and rather troubling, that structure-corrupting aromaticity Kekulization errors by algorithms involve addition or loss of a double bond in “typical” organic molecules. *These can be very hard to detect* when they do not cause a valence violation).

A total of 10,243 substances are rejected during the determination of a canonical tautomer in the *Standardize Valence Bond* step. The reasons for this can be very simple, as shown in Fig. 9c for SID 235635. A final structure sanity check tests for identical charge types on adjacent atoms and rejects structures that test positive. One can look at these as edge cases, whereby the structural representation becomes corrupted in some way. With such diverse structural content, while such cases are potentially fixable (e.g., by means of adjusting hydrogen count or removal of a charge), it usually is a sign of some other molecule corruption or oddity that should be rejected for later manual inspection.

During the conversion of implicit hydrogen atom counts to explicit hydrogen atoms (in the *Standardize Explicit Hydrogens* step), 486 substances are rejected. In most cases the affected structures are oligonucleotides. The addition of explicit hydrogen atoms to the molecule can result in those structures exceeding the current PubChem atom/bond limit of 999 (while not a technical limit, it is a ‘line in the sand’ defining a ‘small molecule’ project scope that may be changed in the future given the increasing number of therapeutic, chemically-modified biopolymers). This restriction mimics the limits of the MDL V2000 MOL file format for chemical structures. Exemplary substances are SID 596521 (a hammerhead ribozyme) and SID 596662 (Ampligen with Amphotericin B).

A structure failing standardization is not necessarily a shortcoming of the standardization approach. In most cases, the rejection of a chemical structure indicates that it does not comply with known/common chemical configurations. Without additional information indicating the original intent of the scientist, the chemical substances cannot be readily normalized and, consequently, are not mapped to a compound. Conflicting or ambiguous chemical structure drawing conventions add a barrier to the creation of normalization rules, as what may correct in one case may corrupt in another.

### Modification rates

We monitored structure modifications during standardization by comparing de-aromatized canonical isomeric SMILES generated before and after each standardization step, as described in the “Standardization modification tracking” subsection of the “Methods” section. We did not include data obtained from structures that eventually were rejected during standardization.

Of the 104,293,434 substances successfully passing the standardization process, 55.5% were not modified at all. The remaining 44.5% were altered in at least one of the standardization steps. The exact numbers per standardization step are presented in Table 2. The steps *Verify Element* and *Verify Valence* evaluate the validity of atom configurations in the molecular structure (as opposed to make changes). Consequently, no structures were modified in those steps. The *Standardize Annotations* step deals with PubChem internal bond annotation that cannot be reflected in SMILES; therefore, no structure modifications could be detected in this step, either.

**Table 2:** Standardization modification rates

	Organic		Inorganic		Mixed		Total	
	(102,177,263 successfully standardized substances)		(52,082 successfully standardized substances)		(2,064,089 successfully standardized substances)		(104,293,434 successfully standardized substances)	
	Modified substances	Exclusively modified Substances	Modified substances	Exclusively modified Substances	Modified substances	Exclusively modified Substances	Modified substances	Exclusively modified Substances
Verify element	–	–	–	–	–	–	0	0
Verify hydrogens	228,654	49,436	–	–	68,629	2598	297,283	52,034
Verify functional groups	226,890	66,911	1680	1678	296,446	121,551	525,016	190,140
Verify valence	–	–	–	–	–	–	0	0
Standardize annotations	–	–	–	–	–	–	0	0
Standardize valence bond	37,258,340	9,643,776	–	–	463,847	97,463	37,722,187	9,741,239
Standardize aromaticity	38,305,291	11,510,081	2	–	444,851	80,666	38,750,144	11,590,747
Standardize stereochemistry	17,614,166	9,738,948	–	–	597,317	407,022	18,211,483	10,145,970
Standardize explicit hydrogens	3190	8	–	–	3580	13	6770	21
Modified substances	45,307,338		1680		1,064,295		46,373,313	
Modification rate	44.34%		3.23%		51.56%		44.46%	

Provided is the number of substances that is modified in each standardization step on the PubChem Substance database as well as the number of substances that is modified exclusively in a given step. The total numbers of substances for every substance class (organic, inorganic, mixed) differ from those provided in Table 1 because structures rejected by standardization were not included in the modification analysis

In the *Verify Hydrogens* step, the (implicit) hydrogen atom counts in 297,283 substances (0.3% of successfully standardized substances and 0.6% of modified substances during standardization) were adjusted to obtain chemically-valid structures. No inorganic substance was modified in this step.

**Fig. 10:**
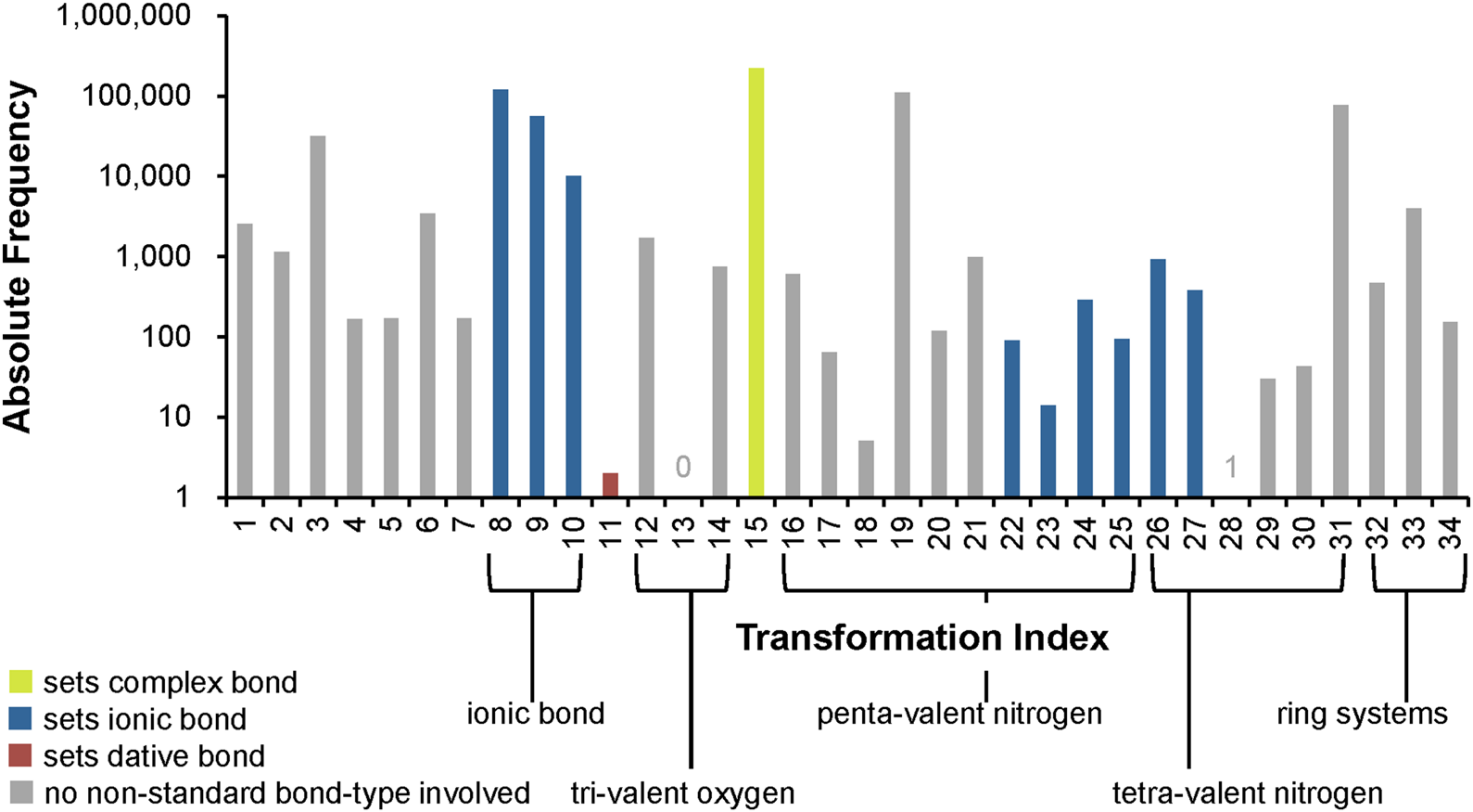
Functional group standardization statistics. A total of 522,757 substances is modified in the *Verify Functional Groups* step, which normalizes non-standard functional group configurations to preferred ones based on a set of standardization rules, each of which is designated with an integer called a “transformation index” for convenience. The total number of substances modified in this step is smaller than the sum of functional group transformations because multiple changes can be performed in the same structure. Nine standardization rules set ionic bonds (8, 9, 10, 22-27), one sets complex bonds (15—the processing of transition metals), and two set dative bonds (11, 28). Rule 13 is not used, indicating that carbon monoxide is only encountered in the correct configuration

The *Verify Functional Groups* step changed the configuration of functional groups in 525,016 substances (0.5% of standardized substances, 1.1% of modified substances). As described in the “Methods” section, this step normalizes non-standard configurations of common functional groups to preferred representations based on a set of 34 standardization rules. The adjustment rates for every rule (as described in the “Methods” section) are presented in Fig. 10. Note that, for convenience, each rule is designated with an integer called a transformation index. In the cases of tri-valent oxygen, penta-valent nitrogen and tetra-valent nitrogen, the total number of matched cases is higher than that of adjusted substances: 60,710 substances with tri-valent oxygen atoms are identified, but only 2442 of them needed adjustment. None of those is the special case of carbon monoxide; this compound was already configured as ^−^C≡O^+^ whenever it was encountered. Penta-valent nitrogen was identified in 112,477 substances and modified in all of them. Tetra-valent nitrogen was identified in 9,090,309 substances, at least one rule was applied in 78,414 cases. Highlighted in Fig. 10 are cases when non-standard bonds (i.e., complex, ionic, and dative bonds) are set. In total, ionic bonds are added in 187,481 substances, complex bonds in 223,467 substances and dative bonds in 3 substances.

The *Standardize Valence Bond* step performs the identification of a canonical tautomer. Consequently, the resonance form may be altered in this step and then again in a later, separate canonicalization. In addition, this step can change bond orders as well as alter hydrogen counts and formal charges. A total of 37,722,187 substances were affected by this step (36.2% of standardized substances, 81.3% of modified substances). The remaining 63.8% of standardized substances were not altered in this step, meaning that they either did not exhibit tautomerism or were already the preferred tautomeric form selected by the PubChem standardization procedure. Therefore, the detected change in 36% of substances may be considered a “lower bound” for the fraction of chemical structures that show some form of tautomerism. This is noteworthy as it is greater than the results obtained in some earlier studies (0.5% [48], 26% [49], 30% [50]).

To get a more accurate estimate for the fraction of structures subject to tautomerism, a more detailed analysis was performed by keeping track of the numbers of tautomers that were generated for every covalently-connected component in every substance (there can be multiple components per substance. Only components with two or more non-hydrogen atoms were considered. Otherwise, they skip this standardization step). Of the 104,293,434 standardized substances, 66,053,812 contained at least one component for which more than one tautomer were generated and evaluated during the valence bond canonicalization step. This means that 63.3% of Substance records show some form of tautomerism, but this number does not consider the redundancy in the Substance database. When multiple substances with the same fully-standardized structure (identified by comparing their de-aromatized canonical isomeric SMILES) are counted only once, 28,417,846 of 45,808,881 unique standardization results (62%) generated more than one tautomer during standardization. This result is comparable to that of the study by Sitzmann et al. [38], estimating more than 67% of chemical structures being affected by tautomerism.

**Fig. 11:**
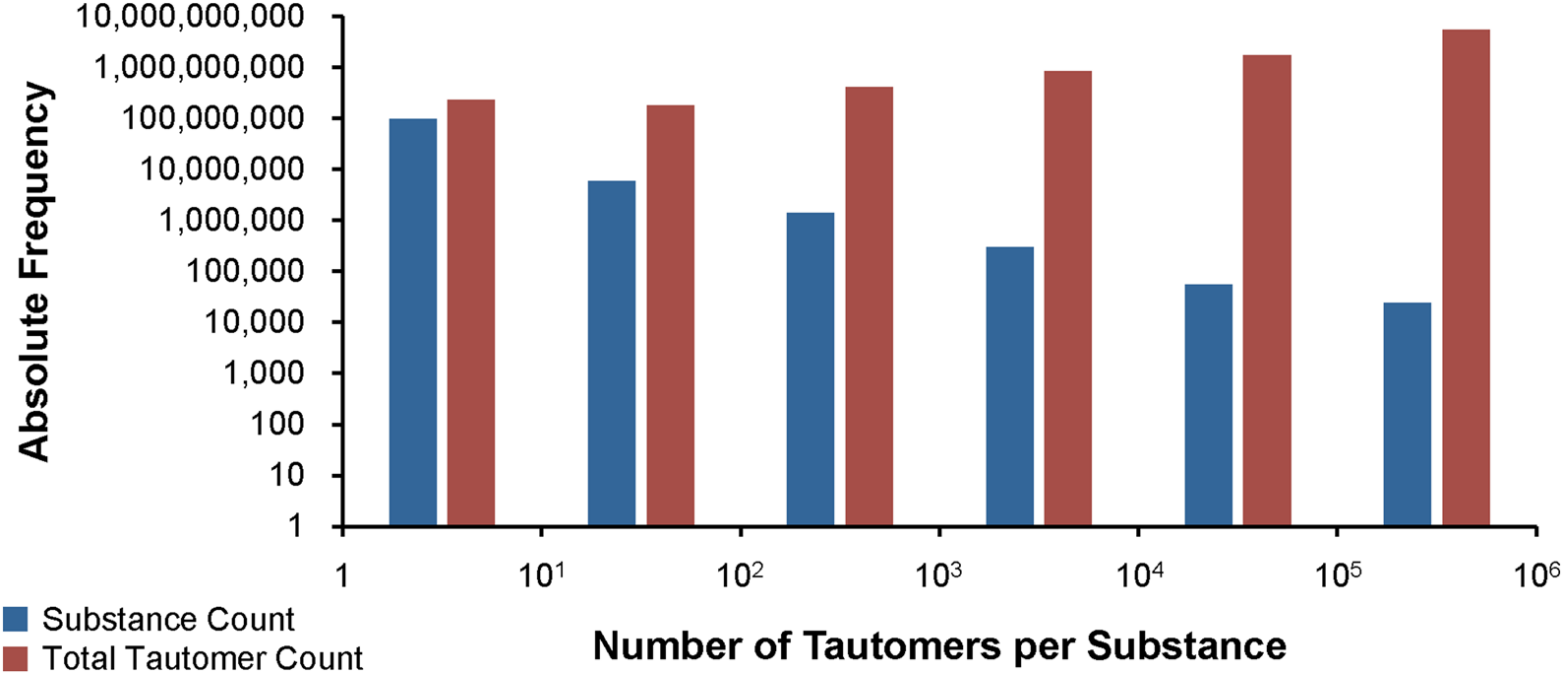
Binned tautomer counts per substance. Histogram is non-cumulative. The first data series (blue) shows how many substances have the respective range of tautomers generated during valence bond canonicalization. The second data series (red) indicates the total number of tautomers generated for those substances in the tautomer count range

**Fig. 12:**
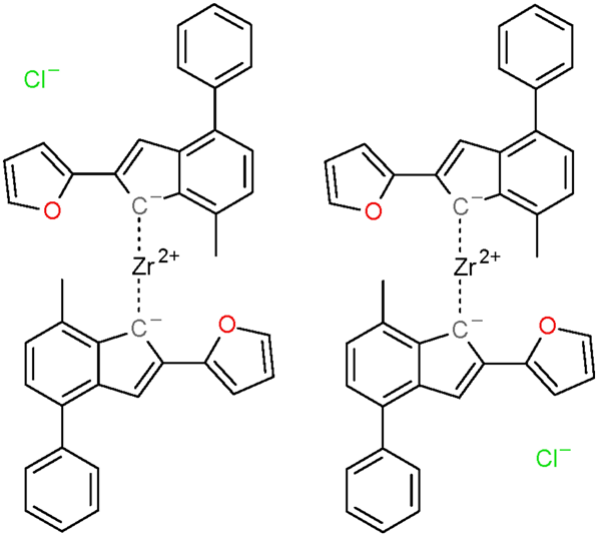
Example for a substance with the highest number of generated tautomers. Shown is SID 30283854 as it enters the step *Standardize Valence Bond Form*. Dashed lines indicate complex bonds as set in *Verify Functional Groups*. Zr and Cl ions skip valence bond canonicalization. Each one of the non-monoatomic connected components reaches the limit of 250,000 generated tautomers. In total, 1 million tautomers are generated during the standardization of this substance, with none of them being considered preferred over the original one by the standardization protocol

The number of tautomers generated for a substance was also computed as the sum of those per-component counts (note: the maximum count of tautomers per component is 250,000. In addition, some components are limited to 25,000, while yet others are prevented from having any tautomers due to memory or computational expense. See the “Methods” section for more details). The resulting per-substance counts of generated tautomers are summarized in the binned histogram found in Fig. 11. In total, 96,421,574 substances (92.5% of standardized substances) were standardized with up to 10 tautomers generated in this processing step. However, the majority (61.2%) of all 8,781,184,002 tautomers generated during the standardization of Substance originated from the 23,778 substances that give rise to between 100,001 and 1,000,000 tautomer forms. The largest number of generated tautomers per substance is one million. The structure of one of those cases (SID 30283854) as it enters valence bond canonicalization is shown in Fig. 12. Each of the four components (with two or more atoms) reaches the enumeration limit of 250,000 tautomers due to negatively charged carbon atoms being allowed in the processing step. Note that this substance is a coordination complex, containing inorganic centers bonded with organic ligands. Most cheminformatics approaches for chemical structure representation cannot adequately handle inorganic and organometallic molecules, which is considered as an unresolved challenge in cheminformatics. With that said, this issue is exemplified by SID 30283854 and other inorganic and organometallic molecules discussed in this paper.

After the identification of a canonical valence bond form, a canonical resonance structure is determined in the step *Standardize Aromaticity*. In 38,750,144 cases, we detected the generation of an alternate Kekulé structure. In this step, aromaticity is perceived and annotated in 96,003,930 substances (92.1% of all successfully standardized substances), indicating that this fraction of structures in Substance has ‘aromatic’ structural elements in the employed perception model. Of the 45,808,881 unique structures after standardization, 41,614,562 (90.8%) contain aromatic systems under the perception model employed in this study.

The *Standardize Stereochemistry* step modified 18,211,483 substances. In 18,067,088 cases, stereo annotation was added to substances that did not have any prior to this standardization step (e.g., to annotate unspecified stereocenters). In 28,327 cases, existing stereochemistry annotation was modified (e.g., placing the stereo wedge on a different bond). In 116,068 substances, annotated stereochemistry was identified as being incorrect and removed [e.g., non-stereogenic Cahn–Ingold–Prelog (CIP)-type centers]. In 6,082,156 substances, existing annotation of stereochemistry was not changed. In total, after this step, 24,177,571 substances had annotated stereochemistry.

The *Standardize Explicit Hydrogens* step affected 6770 substances (0.006% of successfully standardized substances, and 0.015% of modified substances). Here, changes in the de-aromatized canonical isomeric SMILES, which we used for the detection of modifications, can be the result of two effects. First, the standard valence model gets re-applied to the structures, prior to the conversion of implicit hydrogen atom counts to explicit atoms. Second, hydrogen atoms adjacent to chiral atoms are represented as explicit ‘[H]’ in the SMILES strings.

A modification rate of 44.5% in successfully standardized substances (44.3% for organic, 3.2% for inorganic, 51.6% for mixed substances) indicates that almost half of all deposited structures in PubChem are modified by algorithms to provide a consistent structure representation. The standardized structures are used to determine structure equivalency to create unique entries in PubChem Compound and map the original substances (using their SIDs) to the corresponding CIDs. It is important to note that contributed substances are kept in their original state, allowing PubChem standardization rules to be changed as a function of time and re-applied to the original content. This is especially important to keep the original intent and to avoid corruption of structural content that sometimes occurs with coding errors or methodology shortcomings.

### Standardization time statistics

**Fig. 13:**
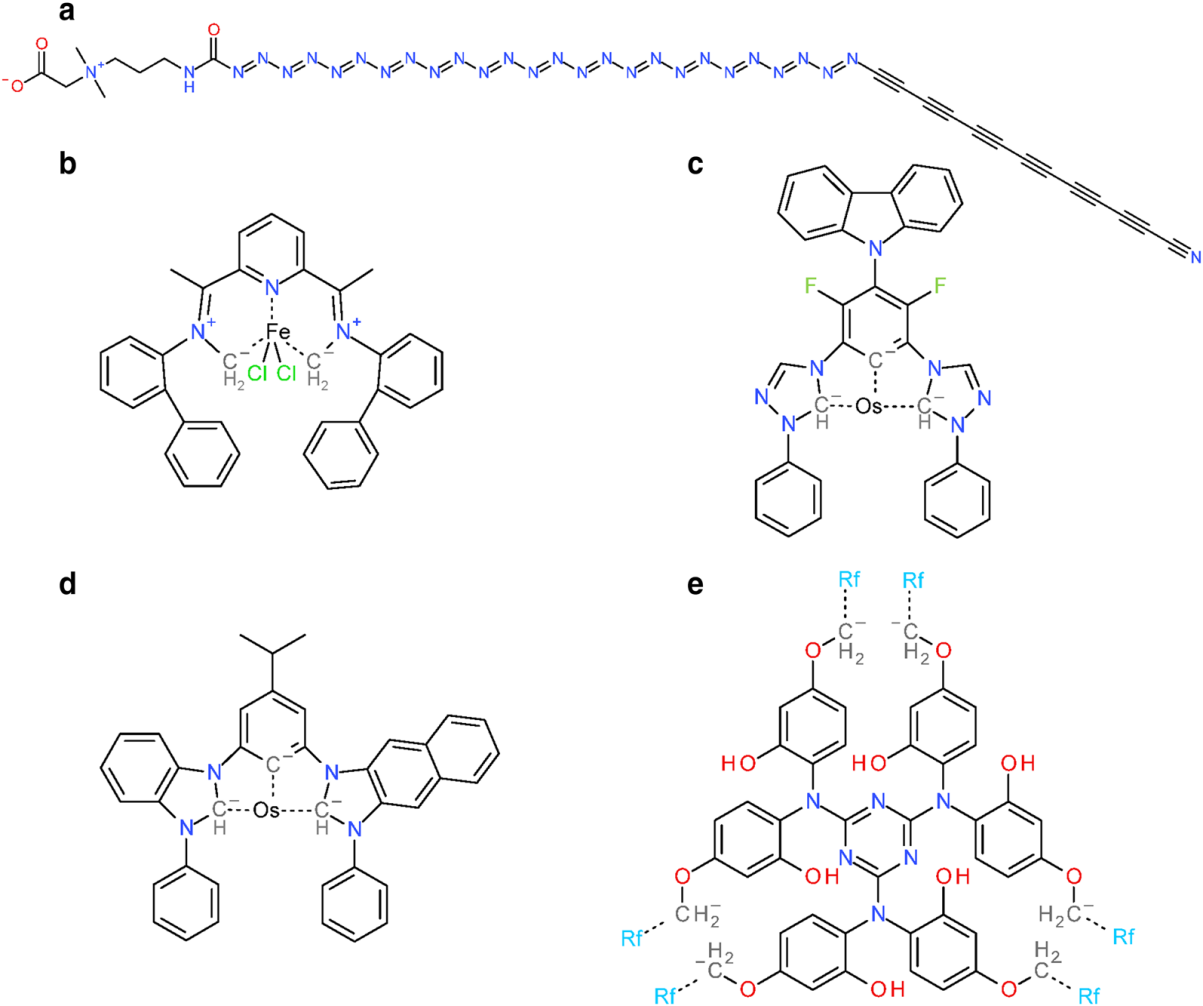
Top-five substances with highest standardization times. Dotted lines indicate complex bonds that were set in conjunction with charges on connected atoms. Shown are fully standardized structures. **a** SID 143137591, 9648 s; **b** SID 142254533, 7555 s; **c** SID 143474510, 3094 s; **d** SID 143474488, 2231 s; **e** SID 138154965, 1187 s

We kept track of the elapsed time spent in each standardization step for each substance. The minimum observed standardization time is 7.99 × 10^−5^ s for SID 42981423 (^81^Sr). All mono-atomic substances have comparable standardization times: the average for standardizing cases is 1.25 × 10^−4^ s with sample standard deviation 3.08 × 10^−3^ and maximum 7.01 × 10^−1^ s (SID 109456853, a phosphorous atom). Measured processing times vary due to the conditions on the heterogenous (many processor types), shared (many different users) compute cluster used for our study and the fact that we could only track wall (actual elapsed) time. The top five substances with the longest standardization time (maximum time 160 min) are presented in Fig. 13. In all five cases, valence bond canonicalization dominated total standardization time (see below for further discussion on filtering out long running cases.) Structures shown in Fig. 13b–e contain charged carbon species that have a major impact on this step. As described in detail in the “Methods” section, charged carbon atoms are not considered during valence bond canonicalization unless they are present in the structure prior to this step. If charged atom types are allowed during the tautomer enumeration, it dramatically increases the number of enumerated tautomeric structures. The analogue case for positively charged nitrogen occurs for SID 143137591, with a maximum standardization time of 9648 s (Fig. 13a). Tautomer enumeration cases resulting in excessive run time are manually limited or completely suppressed from this step periodically by means of examining processing logs. As such, statistics reported here are a lower bound with thousands of cases limited or excluded from analysis. The structures from Fig. 13 had yet to be placed into these excluded and limited cases but help to emphasize the issue as to why they are necessary (see Additional files 2 and 3 for a list of excluded and limited cases, respectively, represented as SMILES). Nearly all cases contain conjugated systems with either positively or negatively charged (carbon) atoms.

**Fig. 14:**
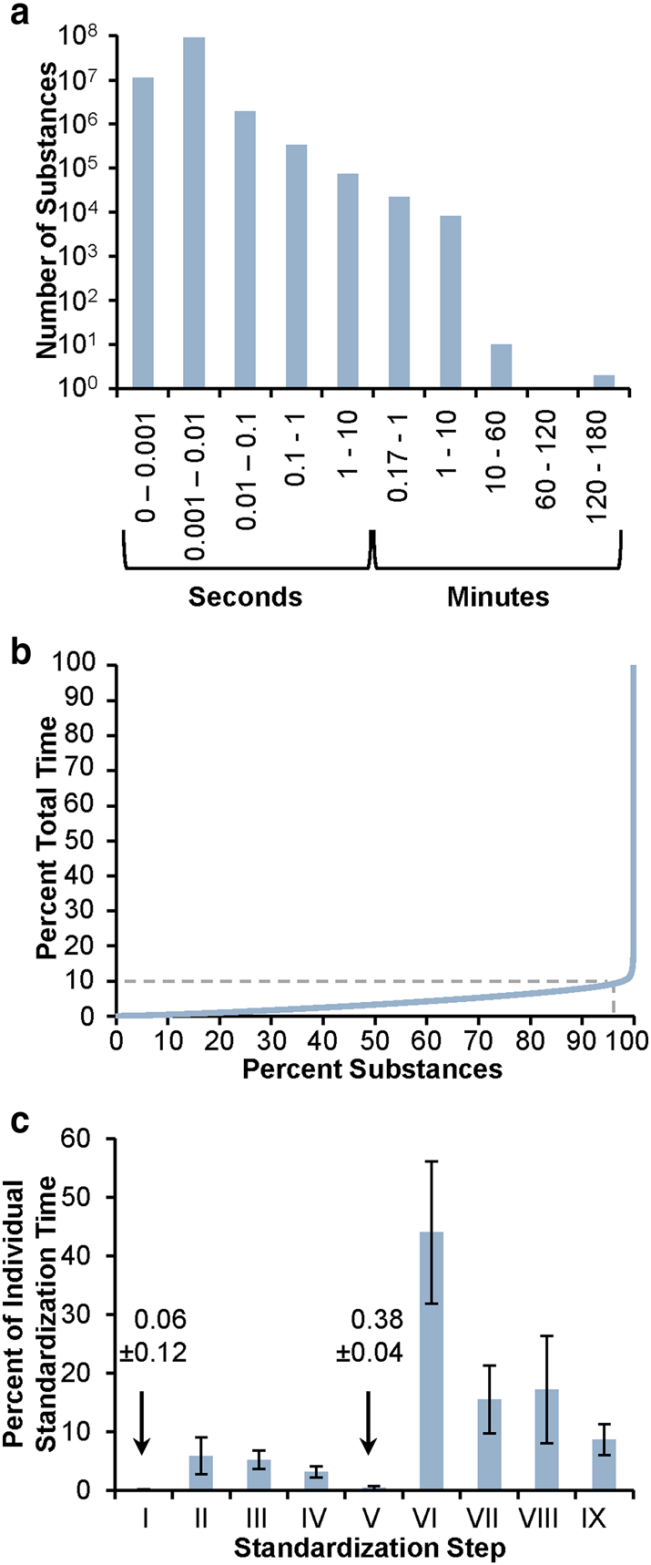
Standardization time statistics. Time was measured as wall time on a mixed-use, heterogeneous compute cluster. **a** Per substance standardization time as non-cumulative histogram. For each bin, the lower (inclusive) and upper (exclusive) boundary is provided. Making the step from s to min, a value of 0.17 min equals 10 s. **b** Cumulative standardization time per substance (sorted by ascending standardization time). 10% of total standardization time is spent on 97.95% of all substances. Within those 97.95%, the average standardization time is 0.0019 s (± 0.0012 s). **c** Average contribution to per substance standardization time per standardization step. Standardization steps are numbered by roman numerals: verify element (I), verify hydrogen (II), verify functional groups (III), verify valence (IV), standardize annotations (V), standardize valence bond form (VI), standardize aromaticity (VII), standardize stereochemistry (VIII), standardize explicit hydrogens (IX). For each substance, the time necessary for standardization is dominated by step (VI), which performs valence bond canonicalization (44 ± 12%)

A binned overview of the standardization time for individual substances is presented in Fig. 14a. The average standardization time is 0.0192 s, with a standard deviation of 1.6205 s. Standardization takes less than 0.001 s for 10.9% of all substances. Most substances (86.8%) have a standardization time between 0.001 and 0.01 s. Consequently, 97.7% of all substances take less than 0.01 s to standardize. The percentile/percentile plot presented in Fig. 14b illustrates that the remaining 2.3% of substances have a standardization time of more than 0.01 s and completely dominate the total time spent in standardization across all substances. Put another way, ~ 98% of all substances can be standardized using only 10% of total standardization time, with an average standardization time of 0.0019 s (standard deviation of 0.0012 s). Conversely, 90% of the standardization time is spent on only ~ 2% of substances.

To demonstrate the relative time per standardization step that consumes the most time, all individual per-step standardization times were normalized to the total standardization time of the particular substance. The resulting average percentages are presented in Fig. 14c. The steps *Verify Element*, *Verify Valence* and *Standardize Annotations* perform no modifications of the molecular graph (instead, they filter out ‘bad’ chemical structures). Consequently, they consume the least amount of time with averages of 0.1%, 3.1% and 0.4% of the time that is used per substance, respectively. The *Verify Hydrogen* step involves the conversion of non-special (e.g., non-isotopic and without stereo-wedge or formal charge), explicit hydrogen atoms into implicit hydrogen. On average, this step consumes 5.9% of the standardization time per structure. The *Verify Functional Groups* step comprises the repeated matching of substructure queries against the molecular graph. Detecting subgraph isomorphisms is an inherently complex problem [98], but due to the small size of substructure queries, the complexity does not fully manifest and the average fraction of per substance standardization time is 5.2%. Most of the standardization time is spent for valence bond canonicalization (in the *Standardize Valence Bond Form* step), with 44.0% of the per substance standardization time. The major computation expense is due, in part, to the approach used. It is not just focused on generation of a canonical tautomer. Rather, it performs a canonic walk through (potentially) many possible tautomeric forms and uses a tautomer scoring function to provide the “best” tautomer representation, as described in the “Methods” section.

Just like the generation of a canonical tautomer, the standardization of aromaticity is a global operation on the molecular graph. Consequently, it is more time consuming than the initial local checks of substructure representations and accounts for 15.5% of the per substance standardization time on average. The standardization of stereochemistry relies on the computation of atomic symmetry classes, which is an iterative procedure on the entire molecular graph. On average, it takes 17.2% of the per substance standardization time. The *Standardize Explicit Hydrogens* step consumes 8.6% of per substance standardization time, a comparable amount of time to its inverse, *Verify Hydrogen*.

In general, the described standardization workflow and its implementation are rather efficient. Only 0.4% of cases take longer than 0.1 s to be individually processed. Yet, those comparatively few cases are responsible for the highest fraction of total standardization time. Steps that involve only atom-wise checks and manipulations are faster than global operations on the molecular graph. Valence bond canonicalization is the most time-consuming step and is a good target for further optimization.

### Unique structure analysis

**Fig. 15:**
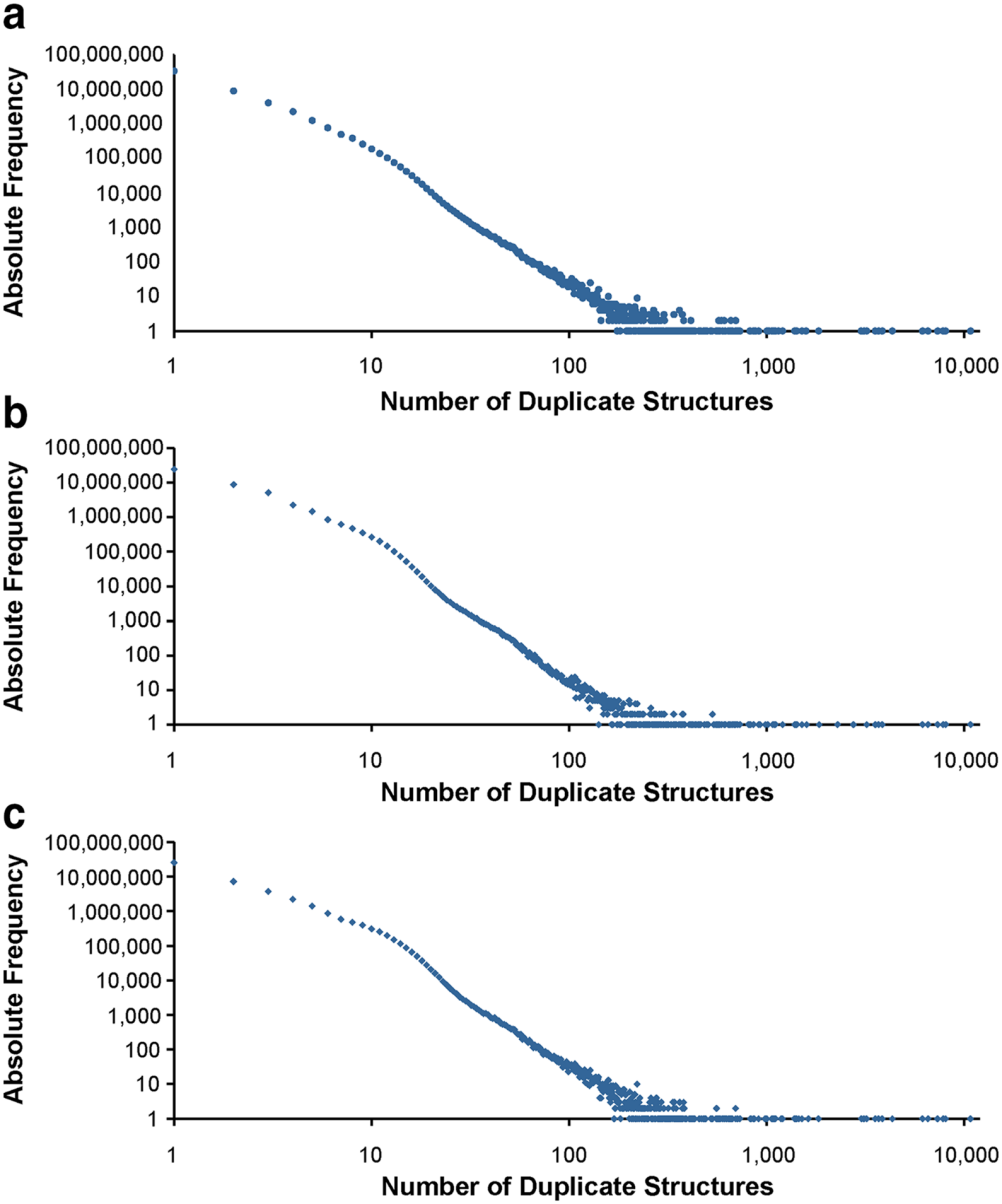
Structure duplicate frequencies in PubChem. Structure equivalency determined by de-aromatized canonical isomeric SMILES before standardization (**a**), after PubChem standardization (**b**) and by standard InChIs (**c**). The x-axis indicates the number of duplicates per structure, *Y*-axis the frequency of this number of duplicates. Plots are double-logarithmic for clarity to emphasize the region of low duplicate counts where the highest differences occur

**Fig. 16:**
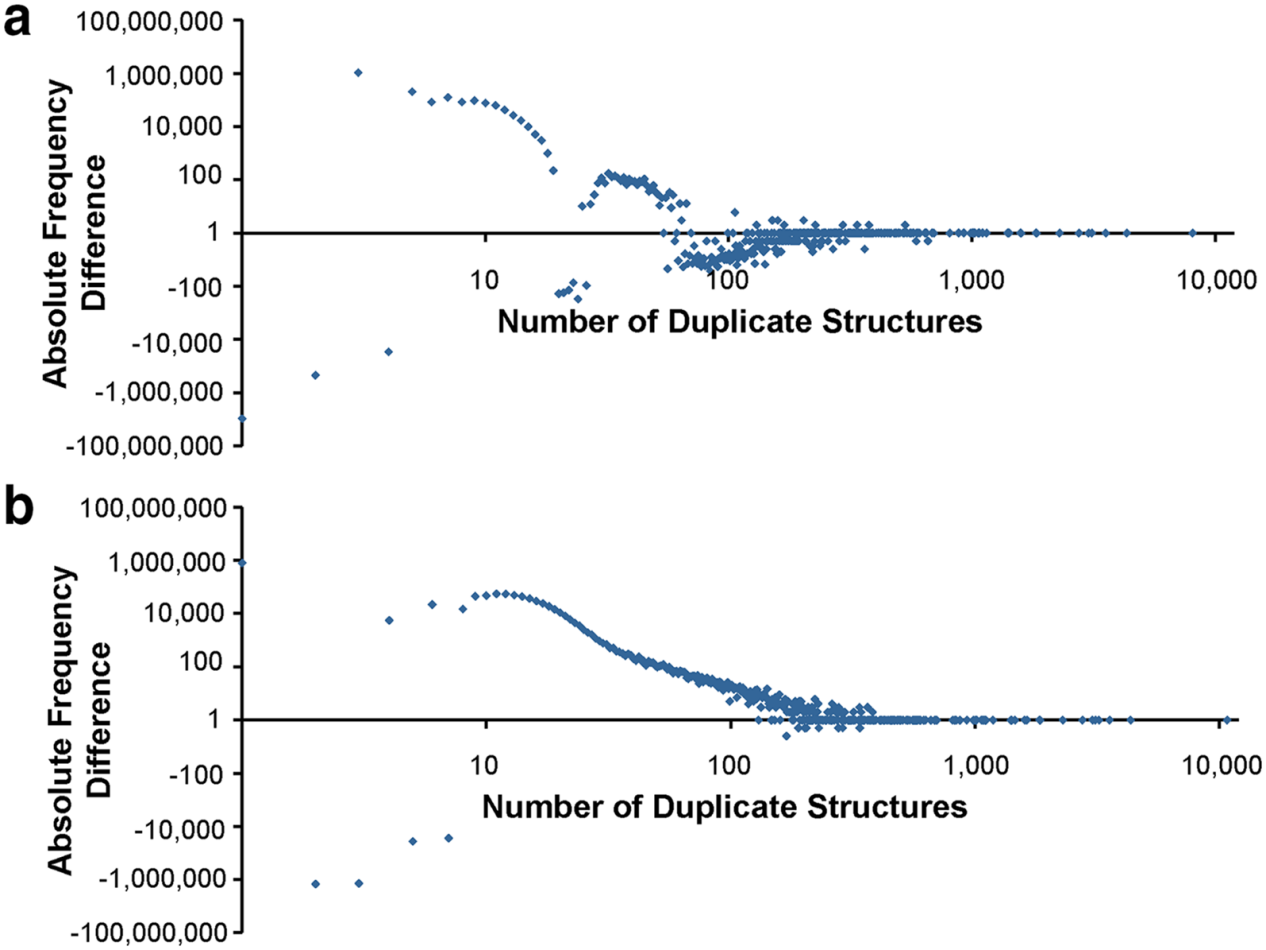
Differences in structure duplicate frequencies. **a** Frequency differences between before and after standardization, structure equivalency determined by de-aromatized canonical isomeric SMILES; **b** frequency differences between PubChem standardization and InChI normalization. X-axis indicates the number of duplicates per structure, *Y*-axis the frequency of this number of duplicates. Plots are double-logarithmic for clarity to emphasize the region of low duplicate counts where the largest differences occur

The effect of standardization on the number of unique structures is clearly noticeable. Before standardization, the 104,293,434 standardizing substances contain 53,574,724 unique structures as assessed by de-aromatized canonical isomeric SMILES, generated as described in the “Standardization modification tracking” subsection in the “Methods” section. This number is reduced to 45,808,881 unique chemical structures after standardization (a reduction of 14.5%). Histograms comparing the frequencies of unique structures before and after standardization are shown in Fig. 15a, b; frequency differences are illustrated in Fig. 16a. There are 34,220,500 singletons in Substance (substances that do not have a duplicate). Standardization reduces that number to 24,794,553 (a reduction of 27.5%). The top five most frequent structures before standardization are: (1) sulfuric acid in the protonated form (occurs 10,762 times); (2) glycerol (occurs 8055 times); (3) Zn^2+^ (occurs 7826 times); (4) Mg^2+^ (occurs 7332 times); and (5) Ca^2+^ (occurs 6557 times). After standardization, the occurrences of these top five most frequent structures remain unchanged, except for glycerol, which occurs two additional times (SIDs 129634019 and 135768721) (in these two substances, the central carbon atom was erroneously configured as a stereocenter, which was corrected by PubChem standardization). After standardization, all substances describing the same chemical structure get mapped to the same CID.

### Comparison to InChI-derived structure

**Fig. 17:**
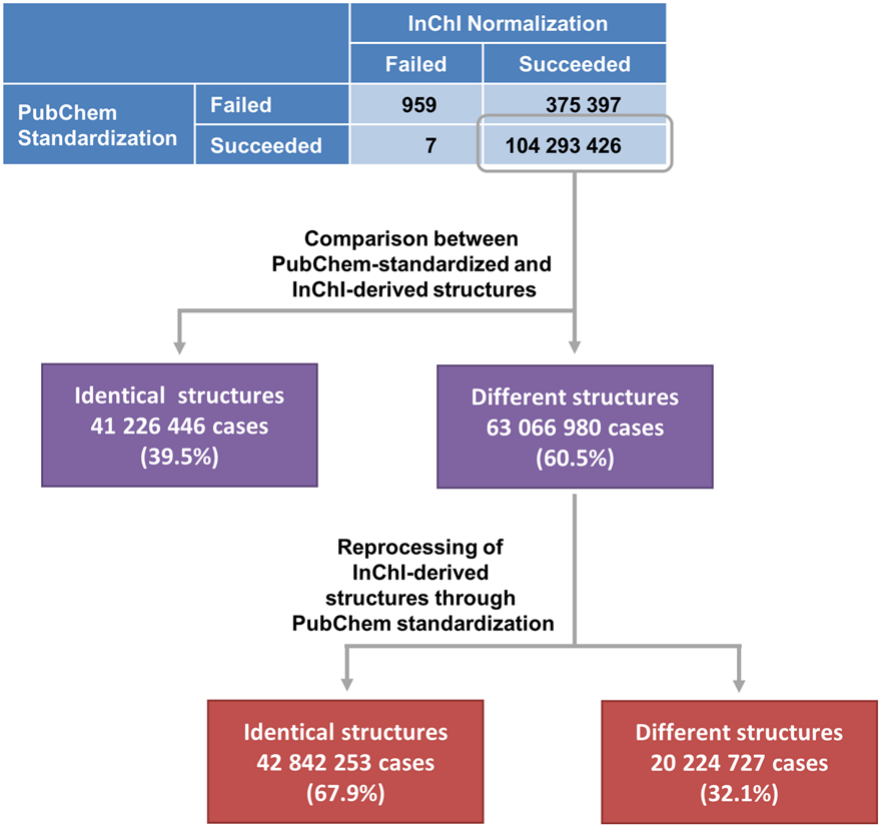
Comparison between PubChem standardization and InChI Normalization

We repeated the analysis of unique structures based on the standard IUPAC International Chemical Identifier (InChI) [11–13] (see Fig. 17) (note: all future reference to InChI normalization refers to standard InChI normalization, which sets specific InChI normalization flags). InChIs could be generated for 104,668,823 substances (99.9991% of all substances that were subjected to the PubChem standardization protocols). These include 375,397 substances (Additional file 4) that are rejected by PubChem standardization for the following reasons:

- 141 failing substances do not pass the initial check of element specifications during PubChem standardization due to invalid isotope specifications. InChI describes the given isotope as delta value to the most common isotope in the ‘/i’ layer. In this process, it seems to accept isotope specifications that are rejected by PubChem standardization (this was verified using the InChI executables: For a wide range of isotopes rejected by PubChem, the difference to the most common isotope is still encoded in the InChI. In the case of very high differences to the most common isotope, isotope specification is omitted in the generated InChI).
- In most cases (364,946 substances), those substances fail the PubChem valence check (Additional file 5).
- In 10,243 cases, substances are rejected in PubChem standardization after valence bond canonicalization for identical charges on adjacent atoms or invalid valences.
- The PubChem standardization protocols rejects 65 substances due to the limit of 999 explicit atoms.

**Fig. 18:**
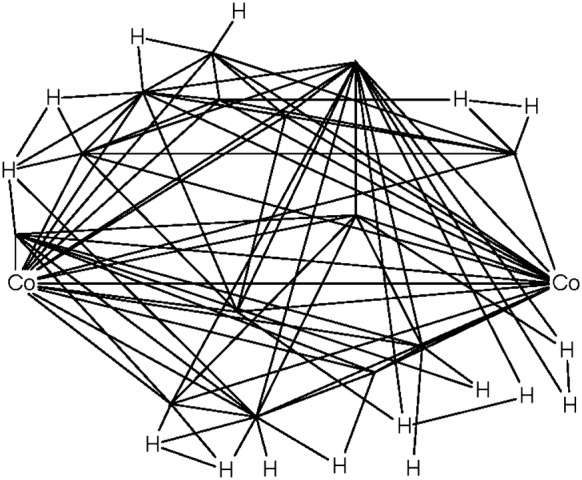
Example structure rejected by InChI normalization and PubChem standardization. SID 7822769 contains various invalid valences and therefore is rejected by both approaches

The list of the 375,397 substances rejected by PubChem standardization and the list of the 364,946 substances that failed the PubChem valence check are provided as supplementary materials (Additional files 4 and 5, respectively). On the other hand, InChI generation failed only in 966 cases in total. Of those, 7 substances passed PubChem standardization. These cases (SIDs 137568629, 140131081, 141166371, 141907841, 142624099, 142915261 and 143373736) involve multiple interactions between organic, metal, alkali metal and transition metal atoms represented as single covalent bonds in the deposited structures. PubChem standardization converts them to complex bonds, whereas InChI normalization fails. The remaining 959 substances fail in PubChem standardization as well as InChI normalization. These are macromolecular structures (e.g., ribozymes or siRNAs) or have multiple invalid valences as illustrated by the odd structure in Fig. 18.

**Fig. 19:**
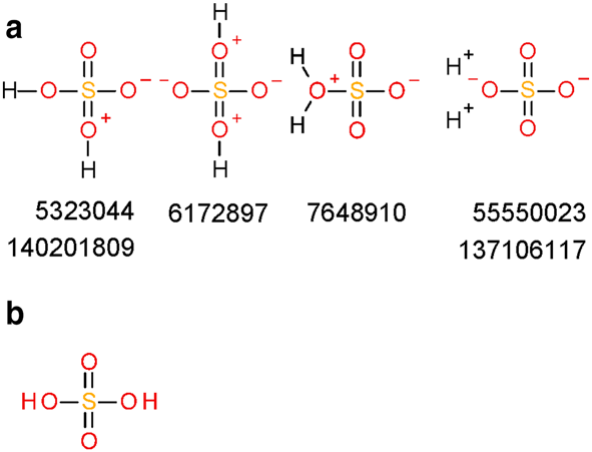
Differences between PubChem-standardized and InChI-derived structures—protonation. Sulfuric acid is the most commonly deposited structure in PubChem. The structures shown in **a** with their SIDs are normalized to the protonated form of sulfuric acid **b** by InChI normalization but not by PubChem standardization, which does not alter them at all

In total, 44,173,224 different structures can be distinguished by their InChIs generated from substances. The histogram of numbers of duplicate structures after InChI normalization is presented in Fig. 15c, which is analogous to Fig. 15b for PubChem standardization. The difference in duplicate structure frequencies between PubChem standardization and standard InChI normalization is illustrated in Fig. 16b. The top five most frequent structures after standard InChI normalization are identical with those after PubChem standardization. The occurrence of sulfuric acid diverges from that obtained from PubChem standardization (10,768 instead of 10,762 times). The additional six substances and standardization results are presented in Fig. 19.

With the increasing popularity of InChI as a chemical representation, some cheminformatics software packages provide the functionality to covert InChI strings into chemical structures. One may wonder how different PubChem-standardized and InChI-derived structures are [here, the InChI-derived structures refer to the structures generated from standard InChI strings using the GetStructFromINCHI() function in the InChI API library]. Therefore, the PubChem-standardized and InChI-derived structures of the 104,293,426 substances that passed both procedures (see Fig. 17) were compared with each other by using the de-aromatized canonical isomeric SMILES strings converted from them. This approach can be likened to Kekulization of an aromatic SMILES. Differences between PubChem-standardized and InChI-derived structures can be manifest in two ways, disagreement on which structures are the same and preference for a structural form. However, complicating a thorough analysis is that the conversion of a standard InChI string into a chemical structure can be problematic, yielding a structure with a different charge or tautomeric state or, especially in the case of metals, missing bonds found in the original structure. As a result, this subsequent analysis helps to identify differences between the PubChem-standardized structure and InChI-derived chemical structure.

For 60.5% (63,066,980 cases) of all the 104,293,426 substances considered, the SMILES strings for PubChem-standardized structures were different from those for InChI-derived structures. To identify differences between the PubChem-standardized and InChI-derived structures, these 63,066,980 InChI-derived structures were subjected to the PubChem standardization protocols and 67.9% (42,842,253 substances) of them yielded the same results as PubChem standardization of the original structures (this means that, although the InChI-derived structure was different, it was the same structure but in a different preferred structural form. In other words, PubChem and InChI normalization approaches agree on structure identity). Structural modifications during the re-processing of the 42,842,253 InChI-derived structures were tracked using de-aromatized canonical isomeric SMILES and are presented in Table 3. For the further analysis of differences between InChI normalization and PubChem standardization we focused on substances that were first modified in a specific standardization step to exclude modifications that were caused by the result of a previous standardization step.

**Table 3:** Modification frequencies in PubChem standardization applied to standard InChI-derived chemical structures

	Modified substances^a^	Exclusively modified substances^b^	First modified substances^c^
Verify element	–	–	–
Verify hydrogen	205,318	36,634	205,318
Verify functional groups	4,759,458	578,750	4,653,740
Verify valence	–	–	–
Standardize annotations	–	–	–
Standardize valence bond form	42,107,606	37,882,115	37,882,174
Standardize aromaticity	28,150,798	10,095	101,021
Standardize stereochemistry	90,424	90,364	90,364
Standardize explicit hydrogens	1558	547	547

For each standardization step on the PubChem substance database, three different substance counts are provided

^a^The total number of substances that are modified in a standardization step

^b^The number of substances that are only modified in the indicated standardization step and not in any of the others

^c^The number of substances that are first modified in the indicated standardization step without having been modified in any of the previous steps

**Fig. 20:**
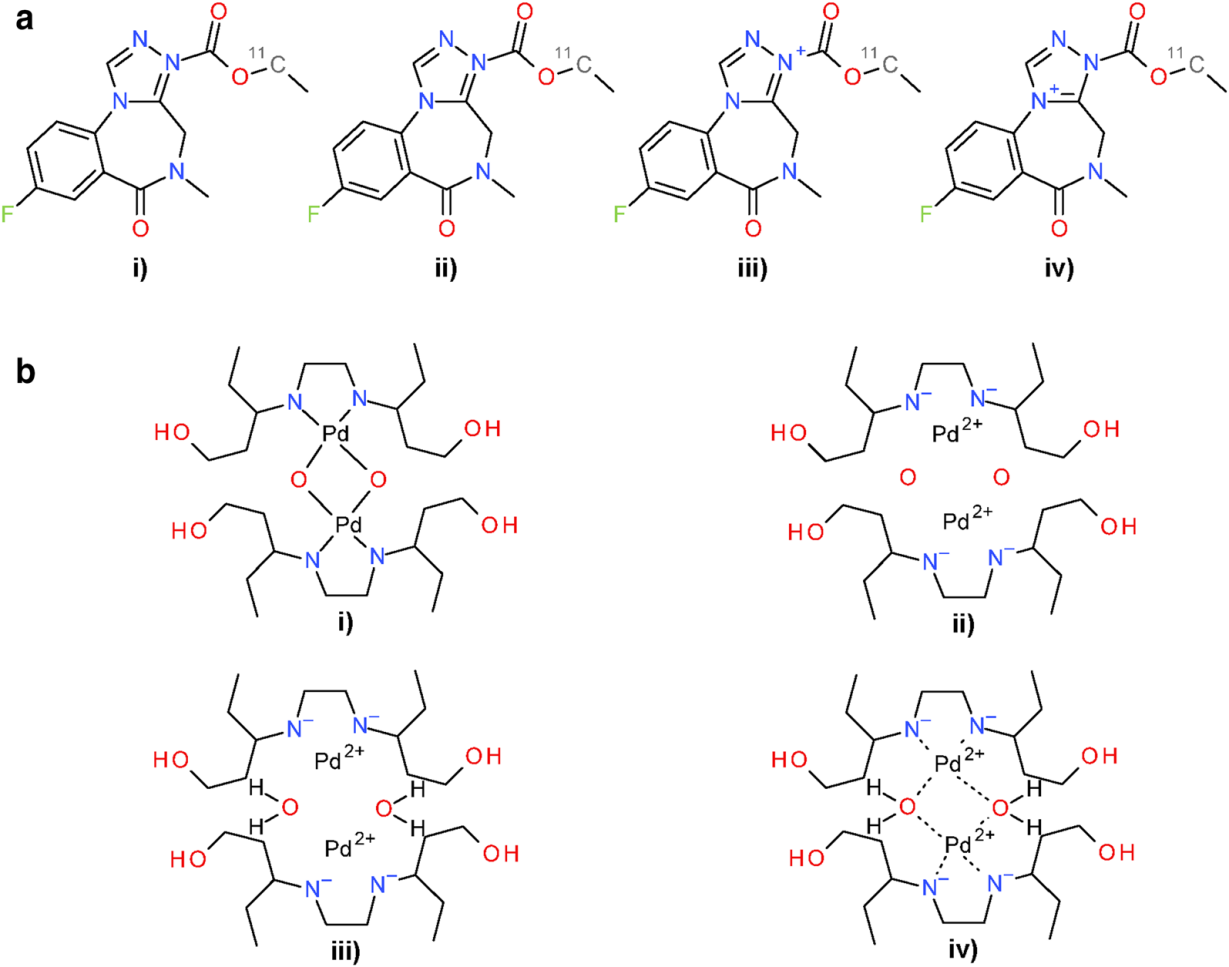
Differences between PubChem-standardized and InChI-derived structures—valence models. Examples illustrate modifications from InChI-derived structures applied in *Verify Hydrogen* (**a**) and *Standardize Explicit Hydrogen* (**b**) during PubChem standardization. **a** SID 1300. State in (i) as deposited. InChI-derived structure results in alternate Kekulé structure (ii). Subsequent PubChem standardization adds positive charge to tetra-valent nitrogen in step *Verify Hydrogen* (iii). The final result of PubChem standardization is shown in (iv). **b** SID 576083. State in (i) as deposited. Standard InChI-derived structure disconnects nitrogen and palladium as well as palladium and oxygen and places charges as appropriate (ii). During subsequent PubChem standardization, two hydrogen atoms are added to each oxygen atom (iii). The result of original PubChem standardization is shown in (iv). Even though (iii) does not possess the complex bonds between nitrogen, palladium and oxygen, the SMILES strings generated for the structures in (iii) and (iv) are identical

Modifications in *Verify Hydrogen* and *Standardize Explicit Hydrogens* indicate differences between valence models used in PubChem standardization and InChI normalization. This leads to changes in the number of hydrogen atoms associated with and/or adjacent to an atom. Examples are shown in Fig. 20.

**Fig. 21:**
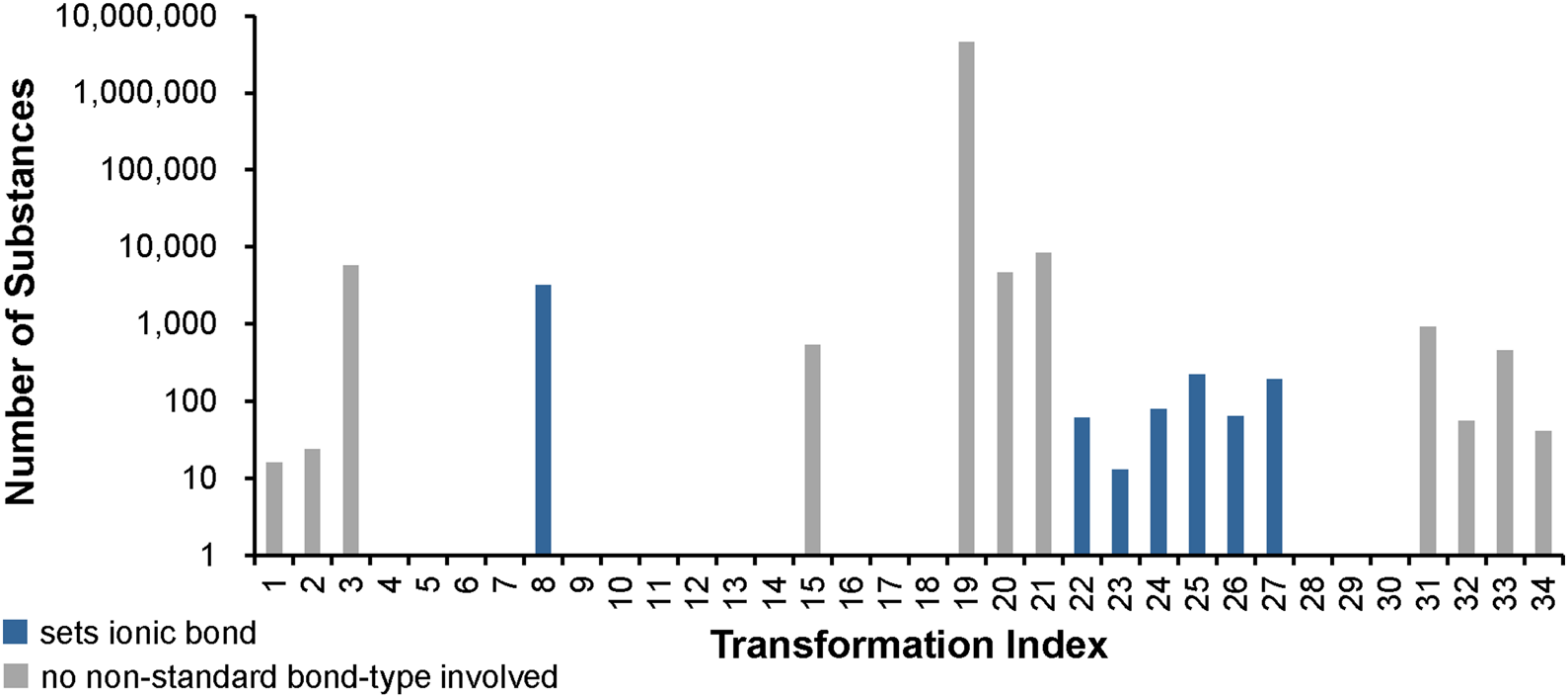
Differences between PubChem-standardized and InChI-derived structures—functional groups. The y-axis indicates the number of affected substances (not the absolute number of modifications in a substance) in the *Verify Functional Groups* step during the PubChem standardization of InChI-derived structures. Transformation indices represent respective standardization rules used to normalize non-standard functional group configurations (see the “Methods” section). All non-visible bars indicate zero affected substances

The *Verify Functional Groups* step performs template-based structural alterations. Analogous to Figs. 10 and 21 shows the statistics for the usage of each transformation rule as invoked during the PubChem standardization of InChI-derived structures (for a description of the actual structure modification, see the “Methods” section). Many substance modifications involve differing preference for functional groups. The most prominent of these involve nitro groups: the InChI-derived N(=O)=O configuration as compared to the PubChem [N+](=O)–[O–] (4,625,069 substances, 22.8% of the total, are affected). The modification rules defining non-standard bond types are used less when applied to InChI-derived structures. This seems logical, as bonds to metal atoms are broken during standard InChI generation.

**Fig. 22:**
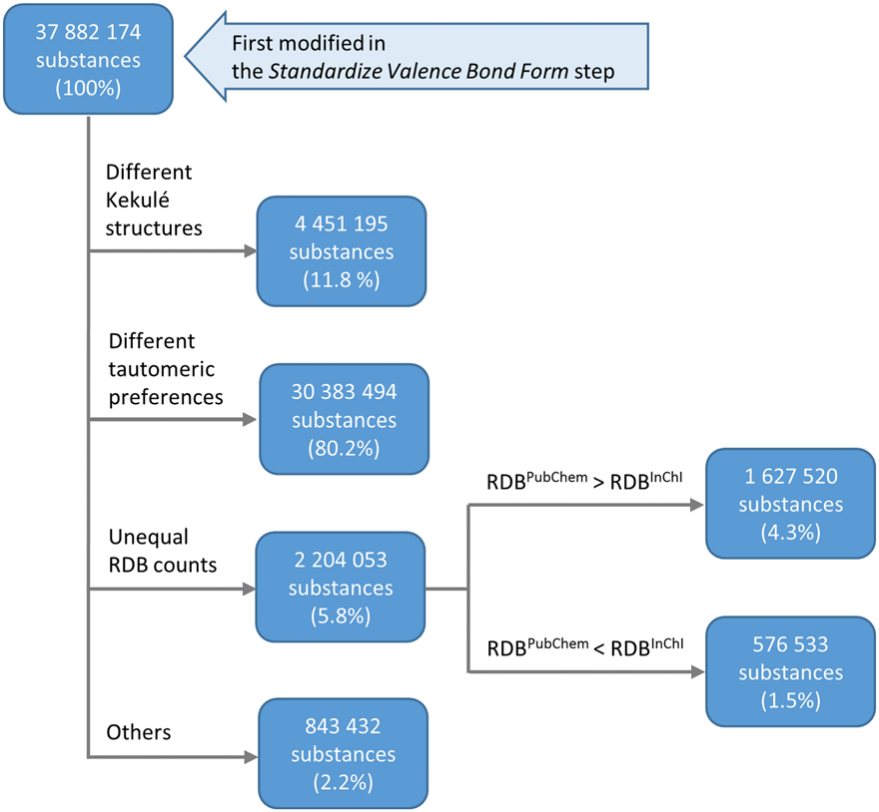
Analysis of 37,882,174 substances first modified in the Standardize Valence Bond Form step during PubChem standardization of InChI-derived structures. Modifications made in this step can be tracked by comparing the de-aromatized canonical isomeric SMILES of InChI-derived structures and PubChem-standardized structures. RDB stands for the count of ring double bonds, and RDB^PubChem^ and RDB^InChI^ are the RDB counts for PubChem-standardized and InChI-derived structures, respectively, emphasizing the tautomer scoring approach in PubChem for exocyclic double bonds

A total of 37,882,174 substances were first modified in the *Standardize Valence Bond Form* step. As shown in Fig. 22, they can be grouped into four classes according to the type of modifications made to them in this step. Due to the similarities of kekulization and tautomer canonicalization, some modifications were merely to different Kekulé structures, corresponding to 4,451,195 substances (11.8% of the 37,882,174 substances).

**Fig. 23:**
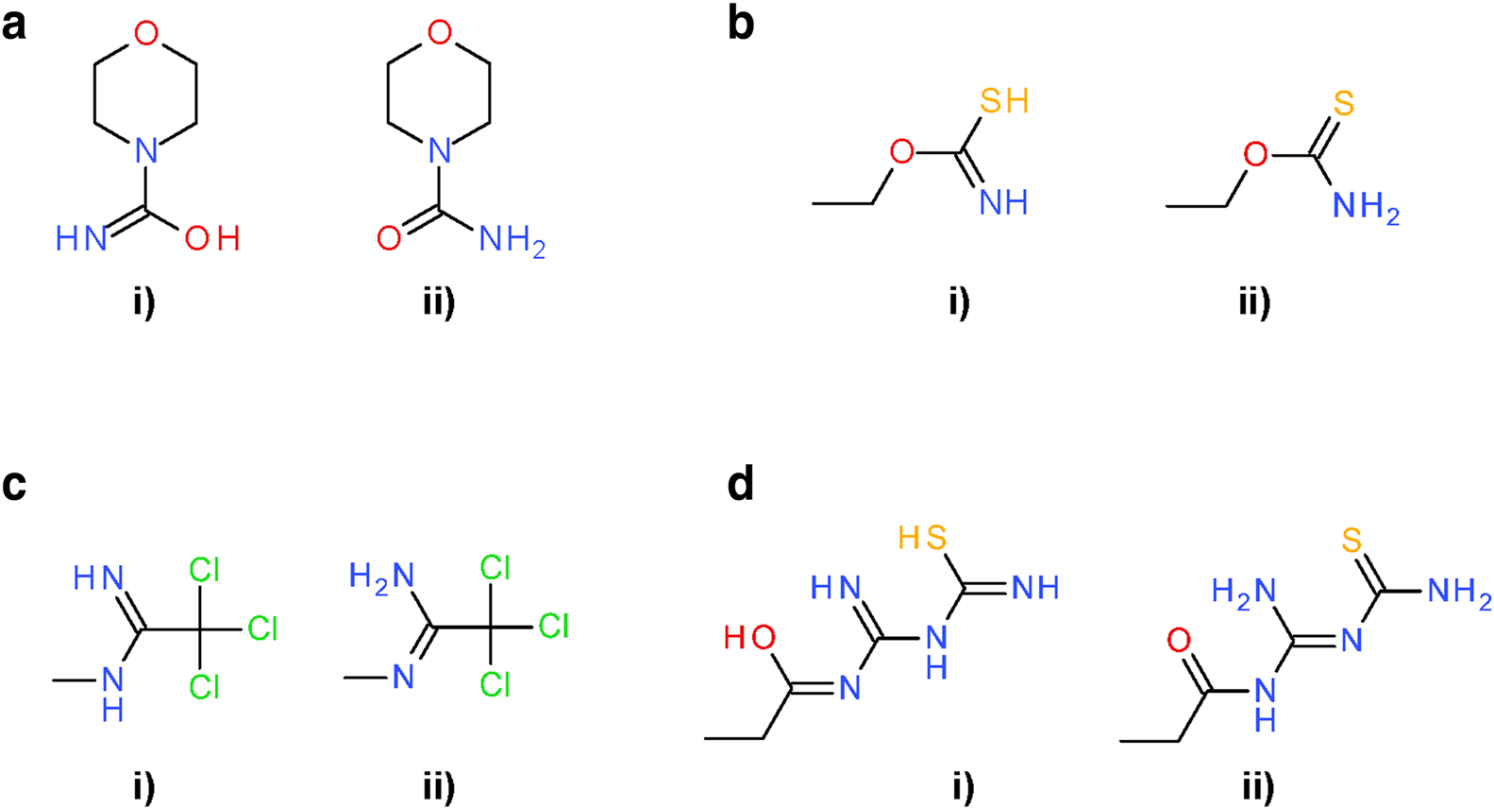
Differences between PubChem-standardized and InChI-derived structures—tautomeric preference in functional groups. Examples for tautomeric preferences of characteristic functional groups. **a** Amide (SID 75764); **b** Thioamide (SID 108898); **c** Amidine (SID 132494); **d** Functional groups and their preferences can occur simultaneously (SID 5856091). In all cases: (i) InChI-derived structure; (ii) structure after subsequent PubChem standardization

**Fig. 24:**
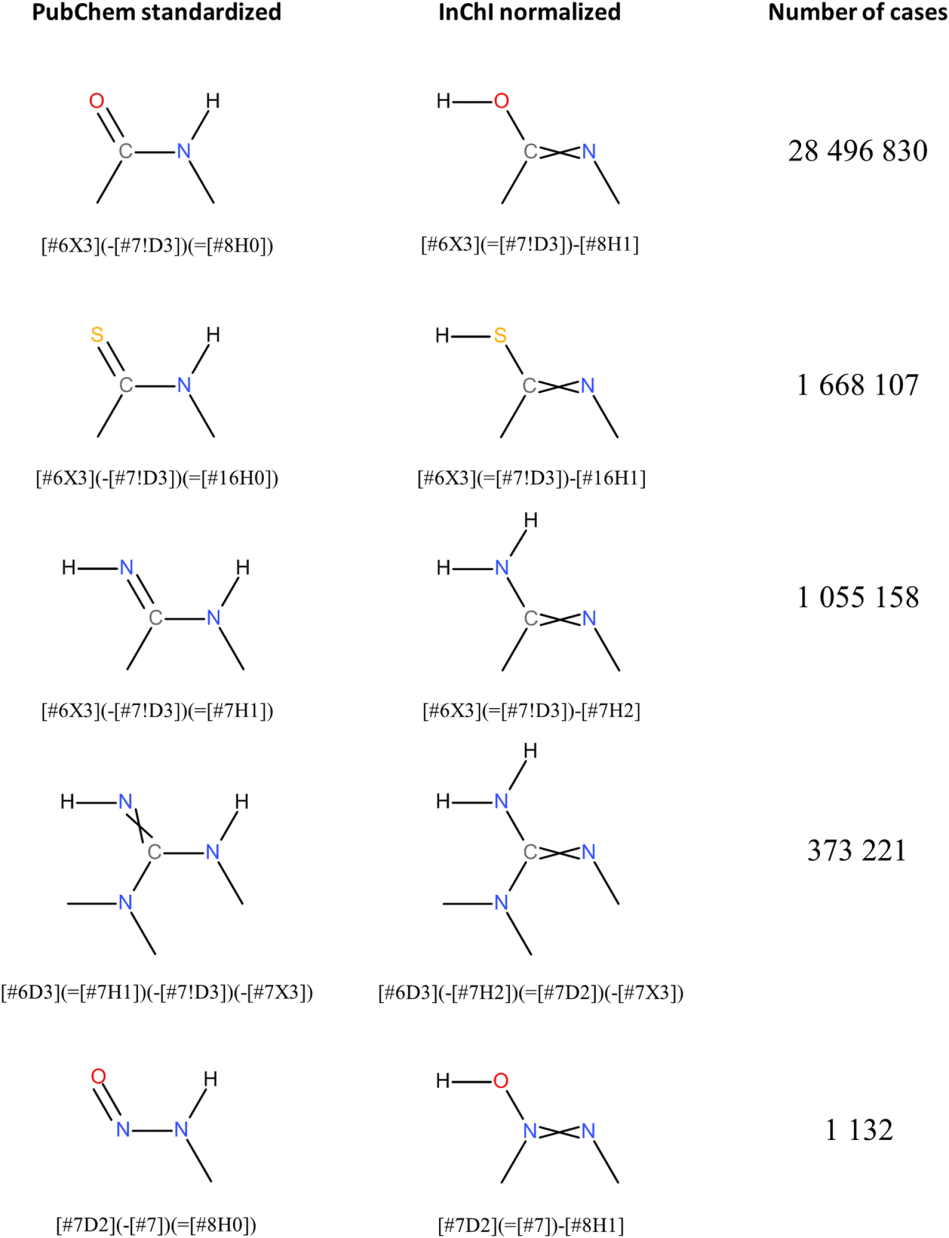
Five types of tautomeric state differences between PubChem-standardized and InChI-derived structures. The difference in tautomeric states between PubChem-standardized and InChI-derived structures are identified using SMARTS. Crossed double bonds are used to indicate stereogenic double bonds with undefined cis/trans configuration

The most common modifications in the *Standardize Valence Bond Form* step was conversion between different tautomers (Fig. 23), observed in 30,383,494 structures (80.2% of the 37,882,174 substances). Figure 24 lists five types of tautomeric conversions tracked using SMARTS strings. Noticeably, conversion from amides to imidic acids, which has been known as the characteristic of InChI-derived chemical structure [99], was most frequently observed (28,496,830 substances), followed by analogous conversion from thioamides and amidine (1,668,107 and 1,055,158 substances, respectively). Interconversion between different tautomeric states for the guanidine and nitrous amide groups was observed in 373,221 structures and 1132 substances, respectively (note: while InChI would appear to make odd choices, e.g., for imines over amides, the InChI is a descriptor. It is not intended to be used as a file format type. InChI-derived chemical structures were never intended to be viewed by scientists, being a canonic representation. On the other hand, PubChem-standardized structures are very visible, forcing care to be taken to pick a canonical structural form that reflects chemist preferences].

**Fig. 25:**
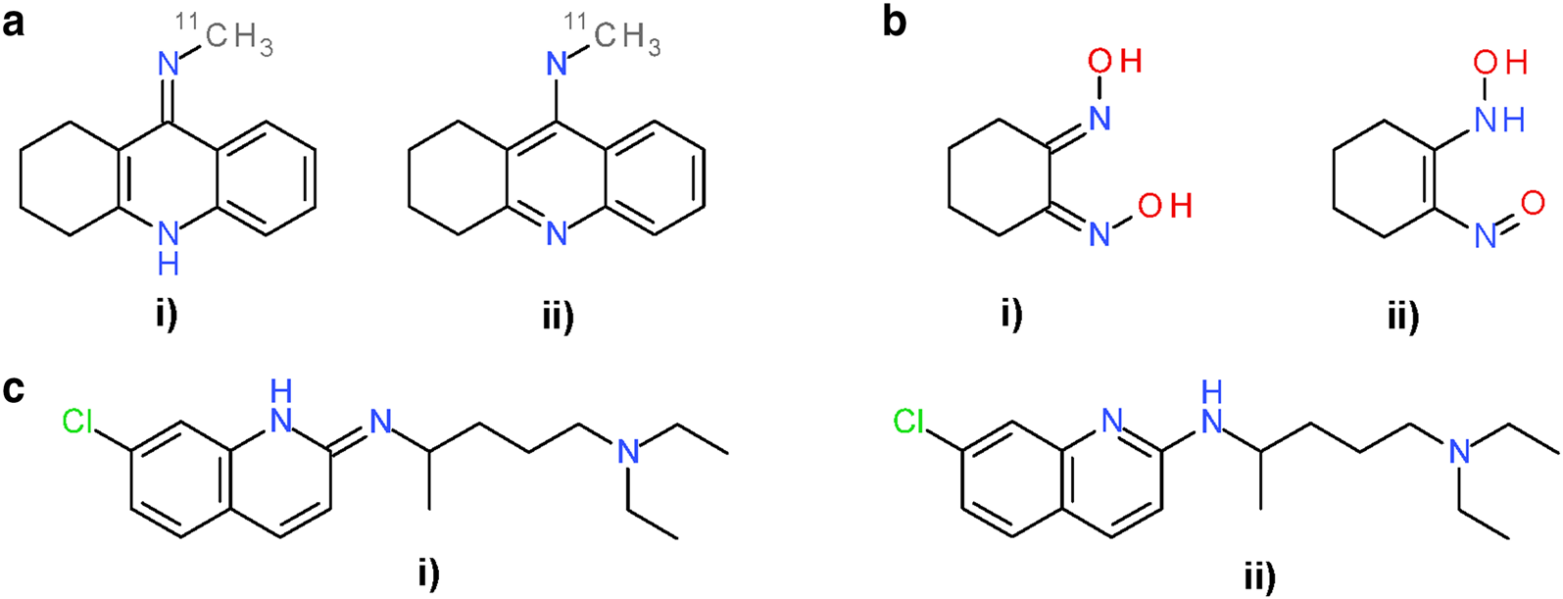
Differences between PubChem-standardized and InChI-derived structures—cyclic double bonds. Examples for preference of cyclic double bonds for PubChem standardization. **a** SID 1462; **b** SID 70471; **c** SID 78008. In all cases: (i) InChI-derived structure; (ii) structure after subsequent PubChem standardization

Still, 3,047,485 substances remain unaffected by the investigated differences (i.e., in terms of Kekulé structures and tautomeric preferences) (Fig. 22). Examples of these cases, as shown in Fig. 25, reveal a tendency of PubChem standardization to keep double bonds in ring systems. Unequal counts of ring double bonds (RDBs) between PubChem-standardized and InChI-derived structures were observed in 2,204,053 substances (5.8% of the 37,882,174 substances) first modified in the *Standardize Valence Bond Form* step (Fig. 22). Among them, 1,627,520 substances had more RDBs in PubChem-standardized structures, and 576,533 substances had more RDBs in InChI-derived structures, revealing that PubChem standardization tends to generate more RDBs than InChI-derived structures. This observation was closely related to how differently exocyclic terminal oxygens are configured in PubChem-standardized and InChI-derived structures (i.e., whether they are single- or double-bonded), because more RDBs are generated when exocyclic terminal oxygens are configured to be *single* bonded. For example, 1,027,027 of the 1,627,520 substances with more RDBs in PubChem-standardized structures contained exocyclic terminal oxygen atoms. In 11,270 of these 1,027,027 cases, PubChem standardization resulted in more of those oxygen atoms being single-bonded, whereas InChI-derived structures generated more single-bonded exocyclic oxygens only in 566 cases. In the majority (1,015,191 cases, or 98.85%), the numbers of single-bonded exocyclic oxygen atoms were identical. On the other hand, of the 576,533 substances for which InChI normalization generated more RDBs, 558,487 substances contained exocyclic terminal oxygen atoms. In 513,567 of these cases, InChI-derived structures resulted in more of those oxygen atoms being single-bonded; in no case did PubChem standardization generated a structure with more single-bonded oxygen atoms. Only in 44,920 cases, the number of single-bonded exocyclic oxygen atoms is the same for both PubChem-standardized and InChI-derived structures.

**Fig. 26:**
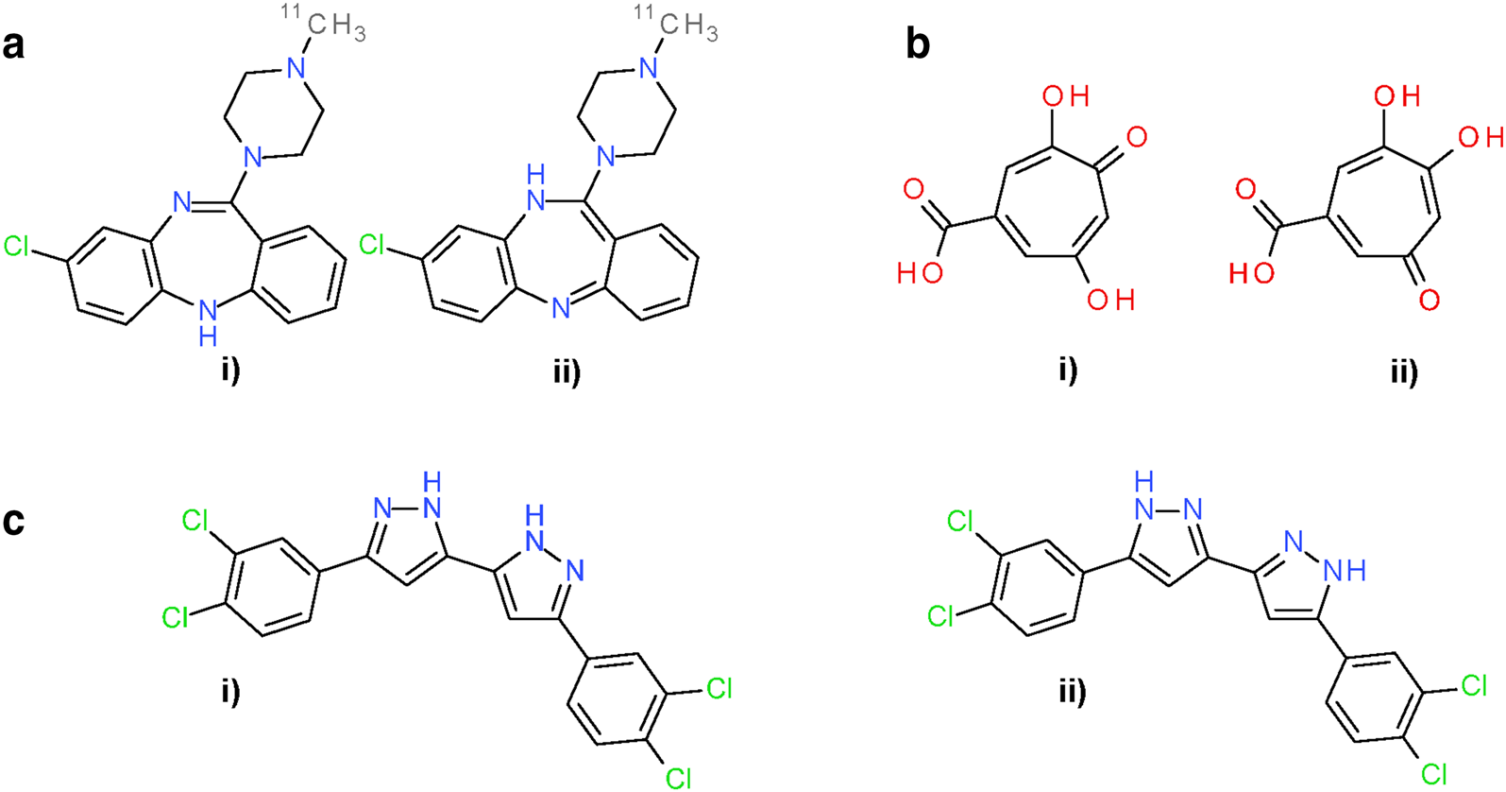
Differences between PubChem-standardized and InChI-derived structures—tautomeric preference. Examples for tautomeric preferences not rooted in specific functional group preferences or the size of conjugated systems. **a** SID 1403; **b** SID 4970. **c** SID 468090. In all cases: (i) InChI-derived structure; (ii) structure after subsequent PubChem standardization

The remaining 843,432 substances first modified in the *Standardize Valence Bond Form* step (Fig. 22) had equal RDB counts for PubChem-standardized and InChI-derived structures. They are examples of longer-range proton transfers as shown in Fig. 26 (note that non-standard InChI normalization allows for longer proton transfers over standard InChI].

Differences noted during the *Standardize Aromaticity* step are rooted in the respective approaches used for the generation of a Kekulé structure. Quoting from the InChI technical manual, “the conversion of aromatic bonds to alternating single and double bonds is done through radical cancellation” [13]. It means that each aromatic atom initially is represented as a radical. Electrons from neighboring such radicals are combined to an additional (pi) bond between them if permitted by their valence. Just as the related PubChem approach, the outcome of this procedure depends on the (canonical) processing order of atoms. This, and consequently the resulting Kekulé structure, cannot be expected to be equivalent between both approaches. However, as the input structures are already valid Kekulé structures without aromaticity perceived and annotated, the InChI-derived structure does not result in any changes of single and double bond patterns and the outcome of PubChem standardization applied to originally deposited structure and InChI-derived structure are identical.

**Fig. 27:**
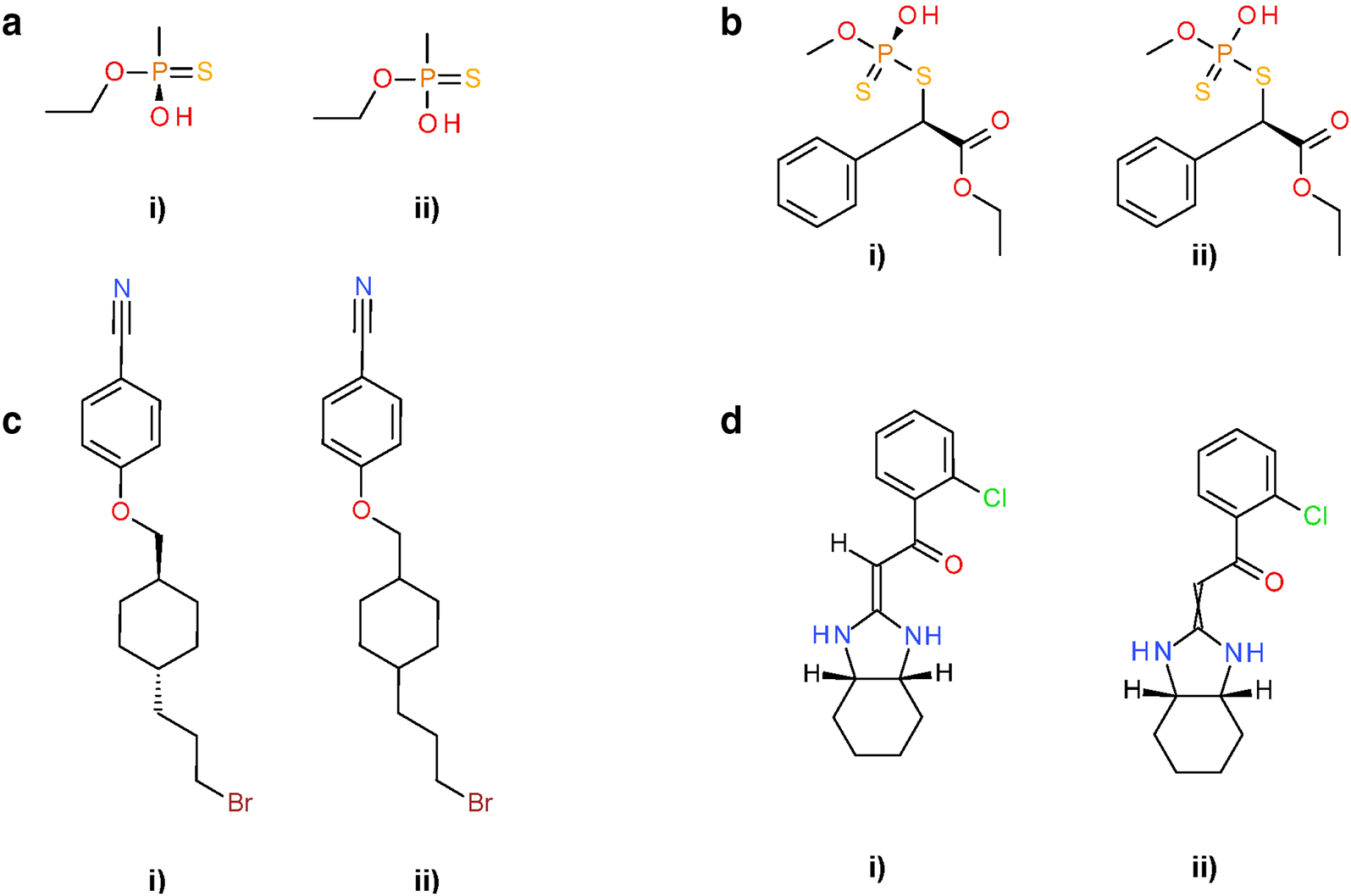
Examples for diverging annotation of stereochemistry in PubChem-standardized and InChI-derived structures. **a** SID 12127575, the phosphorus atom is not considered to be chiral by PubChem standardization. **b** Analogous case in SID 2438124. **c** SID 127817816, PubChem standardization recognizes that the stereogenic carbon atoms do not have neighbors of four different symmetry classes and removes the annotated stereo configurations. **d** SID 158375861, the fully configured double bond in (i) is not considered to be a stereocenter by PubChem standardization due to the identical symmetry classes of adjacent atoms in the ring system. In all cases: (i) InChI-derived structure; (ii) Structure after subsequent PubChem standardization

Differences in *Standardize Stereochemistry* arise from diverging definitions of stereocenters. According to the InChI Technical Manual, P(*)(*)(*)(=*) is recognized as capable of supporting sp^3^ stereochemistry [13]. In PubChem standardization this is not true in general, as certain combinations of ligands that exhibit mesomeric effects negate any annotated stereo configuration (see “Methods” section). This results in a differing number of centers of tetrahedral stereochemistry as illustrated in Fig. 27a, b. The PubChem definition of stereocenters is based on (CIP-style) symmetry classes via OEChem. In some cases, this leads to loss of stereocenters in ring systems (as stereogenic centers may be ignored, as in alicyclic compounds with cis–trans isomerism) when compared to InChI as illustrated in Fig. 27c. The same can be found for double-bond *cis/trans* stereochemistry. As shown in Fig. 27d, in some cases the PubChem standardization protocols do not recognize the same double bonds as stereogenic as does InChI normalization. The bond type C(*)(*)(=*) is generally treated as possibly stereogenic by InChI [13], and the deposited stereo configuration is annotated in the standard InChI. In PubChem standardization, the symmetry groups of adjacent atoms in the example are found to be identical, hence the bond is specified as non-stereogenic. All 90,364 investigated cases (Table 3) differed in the number of stereocenters.

The comparison of PubChem-standardized and InChI-derived structures revealed conceptual differences between the approaches employed to generate them. Identified differences arise from diverging valence models, conventions for the representation of functional groups, tautomeric preference and the definition of stereocenters. In the case of valence bond canonicalization, the approaches are conceptually different. Whereas PubChem standardization aims at identifying a preferred tautomer in a canonic walk using a scoring function, InChI normalization creates a single representation that covers multiple tautomeric states by considering a tautomeric region, which consists of a group of skeletal atoms that share mobile hydrogen atoms involved in tautomerism. The considerable number of unequal InChI-derived/PubChem-standardized structures (60.47% of substances passing both clean-up procedures) shows that those differences in opinion have major impact on the representation of chemical structures. This is especially important considering the increasing prevalence and use of InChI, not only as a chemical descriptor, but also to represent chemical structures (i.e., InChI-derived chemical structure), a use case for which it was never intended.

## Conclusions

The data presented in this study shows that the PubChem structure standardization is an effective and (in general) efficient method that accounts for various sources of molecular diversity and weeds out most improper structures. Its rejection rate for erroneous structures is higher than that of InChI normalization, especially with respect to isotope specifications. The low average processing time (only 0.4% of all substances have an individual standardization time above 0.01 s) and the parallelizability of the problem (embarrassingly parallel) make it suitable for automated compound registration. Yet, the total amount of time necessary to standardize the complete Substance database is dominated by a minority of structures that can be traced to difficulties and inconsistencies in chemical representation when handling organo-metallic complexes (e.g., resulting in negative charges on carbon atoms). A more detailed analysis revealed the generation of a canonical tautomer as the most time-consuming step. The normalization approach used (first developed in 2004 and with periodic major updates between 2005 and 2008) is “ripe” for further optimization, modernization, and improvement.

The representation of chemical structures used in PubChem (after standardization) overcomes problems inherent with chemical information formats. Most prominently, the definition of non-standard bond types (i.e., ionic, complex, and dative bonds) from deposited covalent single bonds remedies their influence on atom valences, ring counts and topological complexity. In this way, PubChem already exceeds what has been recently proposed for the further development of structure file formats [100]. The representation of a stereogenic double bond with undefined *cis/trans* configuration as a crossed double bond is not recommended by IUPAC [101], but it is our opinion that this representation facilitates better understanding of the stereo-configuration of a chemical structure (or lack thereof). It reduces the risk of accidently creating ‘not acceptable’ configurations when using the IUPAC recommended ‘wavy’ bond type. Standardized structures in Compound are made publicly available with explicit hydrogen atoms, eliminating valence ambiguities caused by different implicit-hydrogen valence models.

The comparison to InChI (v1.0.4) normalization and InChI-derived chemical structures revealed discrepancies in tautomeric preference and the definitions of stereocenters. PubChem standardization aims at generating a canonical tautomer with preferred structural properties to enhance its human interpretation. The stereocenter differences could be remedied by an expansion of the stereocenter definitions in PubChem [102–104]. It could also be the basis for further exchange and debate about standards in chemical information, even though the structure standardization problem has not yet found recognition as a grand challenge in cheminformatics [105] or as a hindering factor in computer-assisted drug discovery [106].

With a large pre-existing corpus of structures (tens of millions) complying with diverging approaches, human inspection and curation of structures seems not feasible. Even though ‘RoboChemistry’ is in part responsible for creating the “wasteland” of chemical structures we are dealing with today, automated systems are the only viable option for this task—but they need to be configured, validated, and used with care. The existing standardization system in PubChem faces new challenges every time a new depositor submits data, as the deposition might include chemical representations not seen previously. Any modification to the system must be carefully validated (much like a doctor treating a patient with a promise to “first, do no harm”), with minor changes possibly affecting many thousands of structures. In PubChem, the separation of deposited structures (Substance) and standardized structures (Compound) facilitates the evaluation of alterations to the system, making the creation of a better cleanup and normalization ‘robot’ possible, while keeping provenance clear. As a community, chemical information needs to make progress towards improved digital standards in chemical file formats and chemical structure representation.

## Methods

### PubChem standardization

**Fig. 28:**
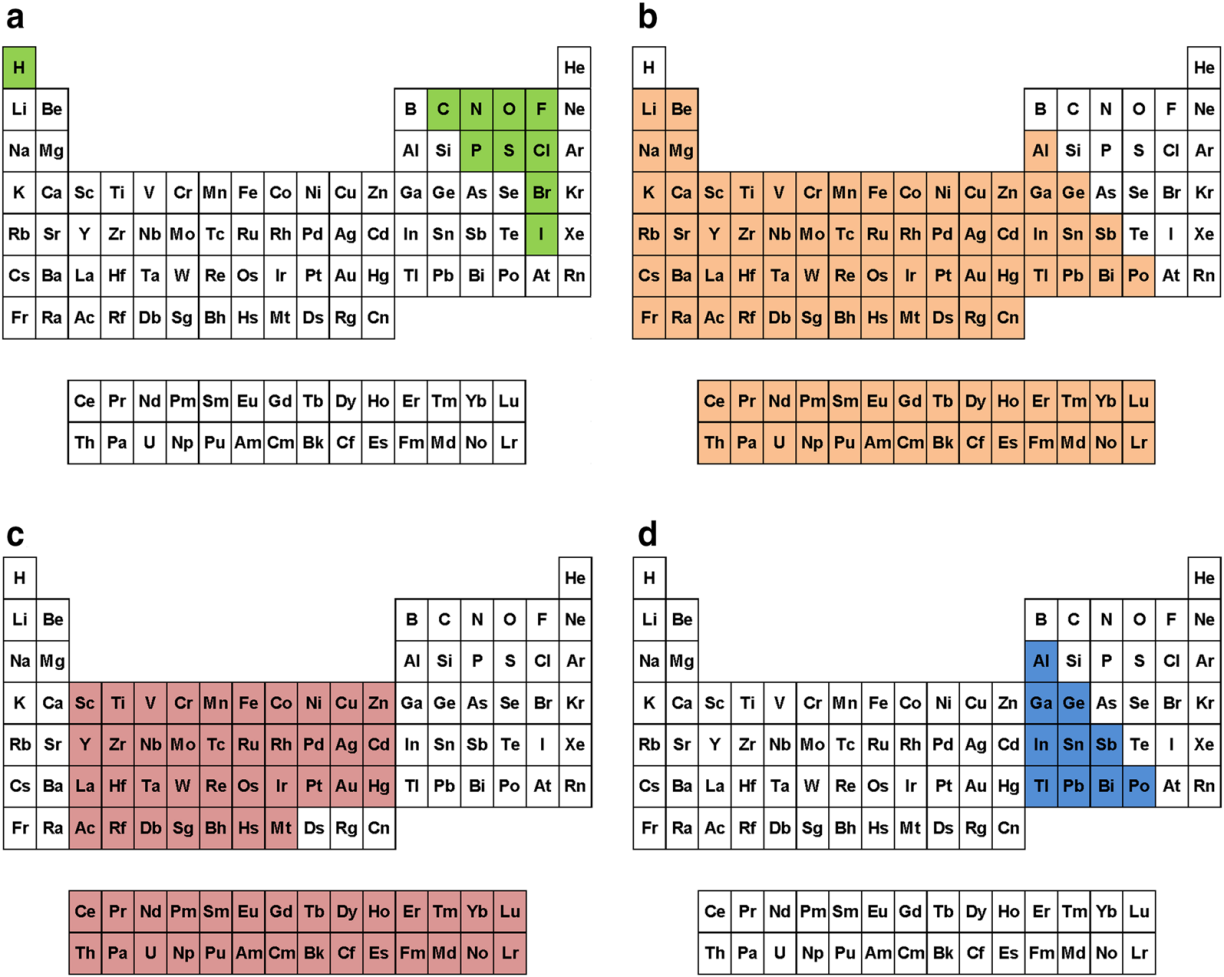
Element classifications as used in PubChem standardization. **a** Organic elements; **b** metals; **c** transition metals; **d** semiconductors. Note that B, Si, As, Te, and At are not included into any element class because of the diversity of bonding possibilities of these elements

The PubChem structure standardization protocols (see Fig. 7) are built on top of the OpenEye Scientific Software, Inc. C++ toolkits [89–92]. It consists of two major phases: structure verification and structure normalization. During verification, atom configurations are checked for their validity with respect to element and valence, as well as in the context of a specified set of functional groups. Valences are corrected as necessary and as possible. The subsequent normalization generates a unique representation with respect to tautomeric state, Kekulé form and the configuration of stereogenic centers, when possible. Each step of the process is described here in detail. The term ‘atom valence’ is used to refer to the number of incident σ and π bonds. The valence of an atom equals the bond order sum of incident covalent bonds (single bond = 1, double bond = 2, triple bond = 3), including those with implicit hydrogen atoms. Elements are grouped into organic elements, metals, transition metals and semiconductors as detailed in Fig. 28. Note that B, Si, As, Te, and At are not included into any element class because of the diversity of bonding possibilities of these elements.

Prior to standardization, a major obstacle in cheminformatics must be addressed: different standards for representing hydrogen atoms. They are typically represented in three ways: (1) as explicit atoms; (2) as a numeric property of atoms; or (3) as implied atoms (e.g., carbon is always tetravalent, with hydrogen being assumed for any valence not already used). In the last case, the implicit hydrogen count of a non-hydrogen atom is determined by a standard value in a valence model. These hydrogen counts are typically based on atomic number, formal charge, and the number and the order of incident bonds. Unfortunately, standard valences can vary between valence models or change for a valence model as a function of time. (For example, in 2017, the default valences for the CTAB/MOL/SDF file format was changed.) Depending on the source of structural information, PubChem deals with all three representations of hydrogen atoms. Consequently, a pre-processing step is performed to unify hydrogen representations. For each atom, implicit hydrogen counts are determined and set according to a simplistic valence model by invoking the function *OEAssignMDLHydrogens* in the OpenEye OEChem C++ toolkit [89]. This model assumes that bond orders and formal charges on atoms are correct and adds implicit hydrogen atoms using the available information. This is used as a simple starting point and adjusted in subsequent steps.

In addition to covalent bonds, PubChem internally supports three non-standard bond types: ionic, complex, and dative bonds.

- Ionic bonds are set in cases where the ionic character of a bond clearly outweighs the covalent part [i.e., when an alkali metal or alkaline earth metal is bonded to an organic element (see Fig. 28)].
- Complex bonds are used to describe coordination complexes. They occur mostly in interactions of organic elements to transition metals and are also used to represent metal–metal bonds. Prominent examples for this bond type are the bonds to central iron and magnesium ions in hemoglobin and chlorophyll, respectively.
- In a dative bond (also known as a dipolar bond), an electron pair is shared between interacting partners, making one the donor and the other one the acceptor. Compared to a covalent bond, where every bonding partner contributes an electron, this bond type has higher polarity, and is weaker and longer. They are annotated in PubChem without placing charges on the bonding partners.

**Fig. 29:**
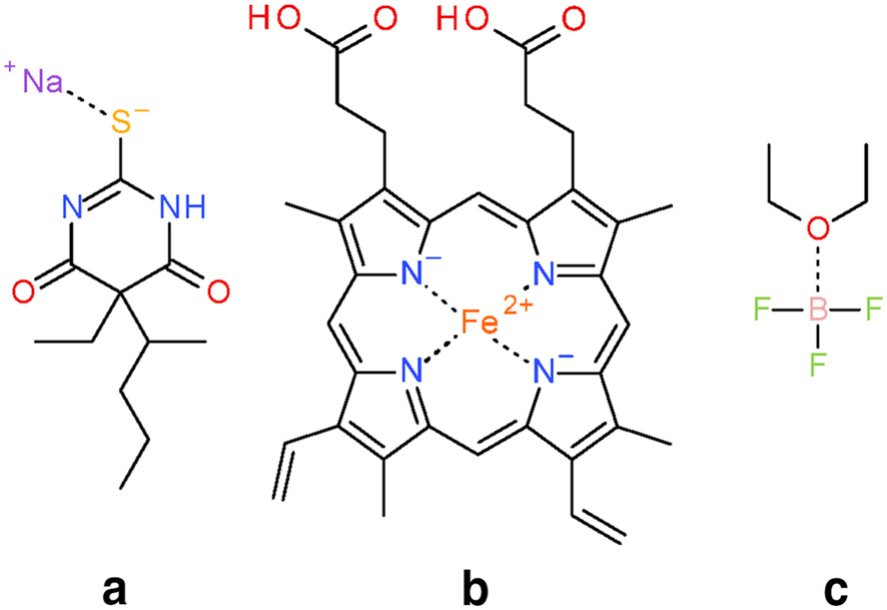
Non-standard bond types in PubChem. **a** Ionic bond between sodium and sulfur in sodium thiopental (CID 23665410); **b** Complex bond between nitrogen atoms and Fe(II) represented as Fe^2+^ in heme b (CID 4973); **c** Dative bond between boron and oxygen in boron trifluoride diethyl etherate (CID 517922). Contrary to other annotations of this bond type, in PubChem, the participating atoms do not get assigned charges

Examples of these bond types in PubChem are shown in Fig. 29. All three bond types are perceived and annotated during standardization. If non-standard bonds are present in a structure, they are indicated as such in the provided structure depiction and annotated in the downloadable files on the PubChem FTP site in Abstract Syntax Notation One (ASN.1, which is the archival format of the PubChem resource), Extensible Markup Language (XML), and Structure-Data File (SDF) format. In the case of SDF files, they are annotated in an associated PubChem-specific SD data field. Non-standard bond types have no influence on atom valence (i.e., they are so-called ‘zero-order bonds’) [100]. By the usage of these three bond types, in addition to those commonly employed in definitions of the molecular graph, PubChem already goes beyond what has been proposed by other sources for future structure file formats in chemical information [100].

In the following subsections, we describe the structure verification and normalization processes performed during PubChem standardization. The verification process consists of atom-based validity checks and modifications. In this way, it is ensured that only structures consisting of valid and reasonably configured atoms are considered in the subsequent normalization process.

#### Verify element

This step evaluates the validity of provided element and isotope information. First, the atomic number of each atom in the structure is checked for its validity. Second, it is determined whether the provided isotope is known and valid. An internal knowledgebase from NUBASE2012 of allowed isotopes is applied. Isotopes are restricted to include only those with a half-life longer than 1 ms (isotopes with shorter half-lives can exist in the Substance database but are excluded from the compound database).

#### Verify hydrogen

The verification of hydrogen atoms aims at generating a representation of the provided chemical structure that only uses implicit hydrogen atoms (as-is possible). For this purpose, explicit hydrogen atoms are converted to implicit ones by incrementing hydrogen counts of the connected atom (count increments by 1 for every deleted explicit hydrogen atom). Excluded from this conversion are hydrogen atoms in H_2_, H^∙^ radicals, and H^+^ or H^−^ ions. Furthermore, the hydrogen atom to be deleted must be connected to an organic atom with a single covalent bond, must not be allowed to have a charge or be isotopically labelled, and must not be incident to an annotated stereo ‘wedge’ bond. If any of those criteria are not met, the explicit hydrogen atom is not removed and the implicit hydrogen atom count of its adjacent atom is not incremented.

Next, a simplistic valence model is applied to molecules with non-zero counts of implicit hydrogen atoms to prevent them from having improper (implicit) hydrogen counts for all (non-hydrogen) heavy atoms. The following changes are made for uncharged heavy atoms:

- Arsenic, phosphorus, and nitrogen atoms with a valence of 5 get assigned a formal charge of + 1 and their implicit hydrogen count is decreased by 1, thus reducing the valence by one.
- Selenium or sulfur atoms with a valence of 6 or 4 get assigned a formal charge of − 1 and their implicit hydrogen count is decreased by 1, thus reducing the valence by one.
- Iodine, bromine, or chlorine atoms with a valence of 7, 5 or 3 get assigned a formal charge of − 1 and their implicit hydrogen count is decreased by 1, thus reducing the valence by one.
- On non-organic atoms (see Fig. 28), the implicit hydrogen count is set to a default value of 0, thus preventing implicit hydrides. (e.g., ‘Li’ does not become ‘LiH’).

#### Verify functional groups

**Fig. 30:**
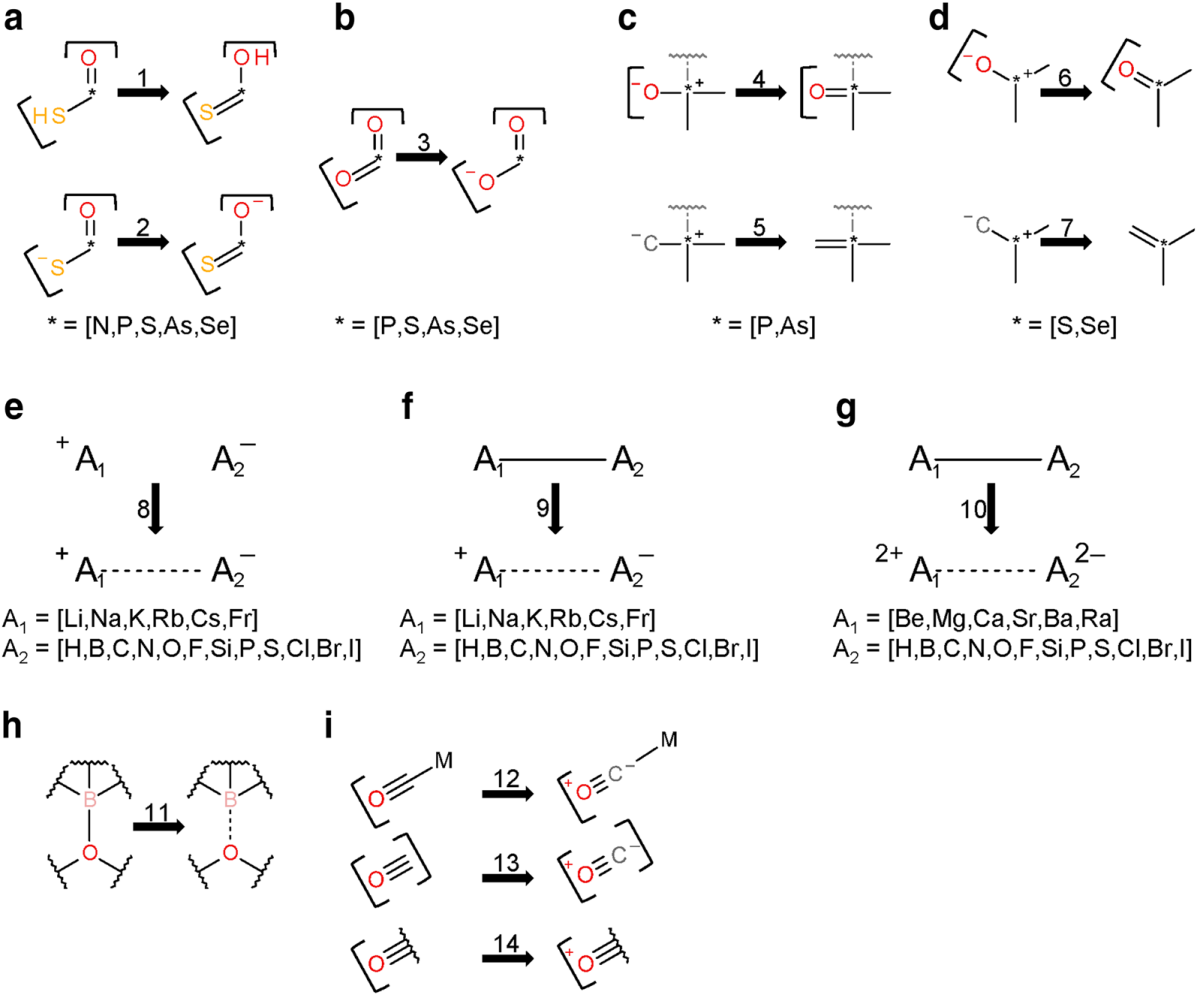
Functional group standardization I. If not mentioned otherwise, hydrogen atoms are as depicted and wildcard asterisks (*), representing connected any atoms, can be hydrogen atoms. Connected carbon atoms are shown without labels and should not be confused with ‘any’ connections. Parenthesis indicates terminal atom. Numbers above arrows are transformation indices for respective standardization rules (see the text for the description of transformation indices). **a** Oxygen and sulfur terminal; no implicit hydrogens on central atom. **b** Both oxygen atoms terminal, no implicit hydrogens on manipulated oxygen or center atom. **c** Center atom has one more explicit connection that is not further specified (with respect to bond order and adjacent atom). Oxygen is terminal, but carbon does not have to be terminal. Center atom and charged partner have no implicit hydrogen atoms. **d** Oxygen is terminal. Hydrogen atoms on uncharged carbon atoms are not checked. Center atom and charged partner cannot have implicit hydrogen atoms. **e** Ionic bond is set if situation is unambiguous, with A_1_ and A_2_ being the only matches of their kind. **f** No charges are assigned if A_2_ is di-valent oxygen or tri-valent nitrogen (after modification to ionic bond). Charge modification is incremental. Charge limit is + 1 on A_1_ and − 1 on A_2_. **g** No charges are assigned if A_2_ is di-valent oxygen or tri-valent nitrogen (after modification to ionic bond). Charge modification is incremental. Charge limit is + 2 on A_1_ and − 2 on A_2_. **h** Bond is annotated as dative bond. **i** M is a metal as defined in Fig. 28

**Fig. 31:**
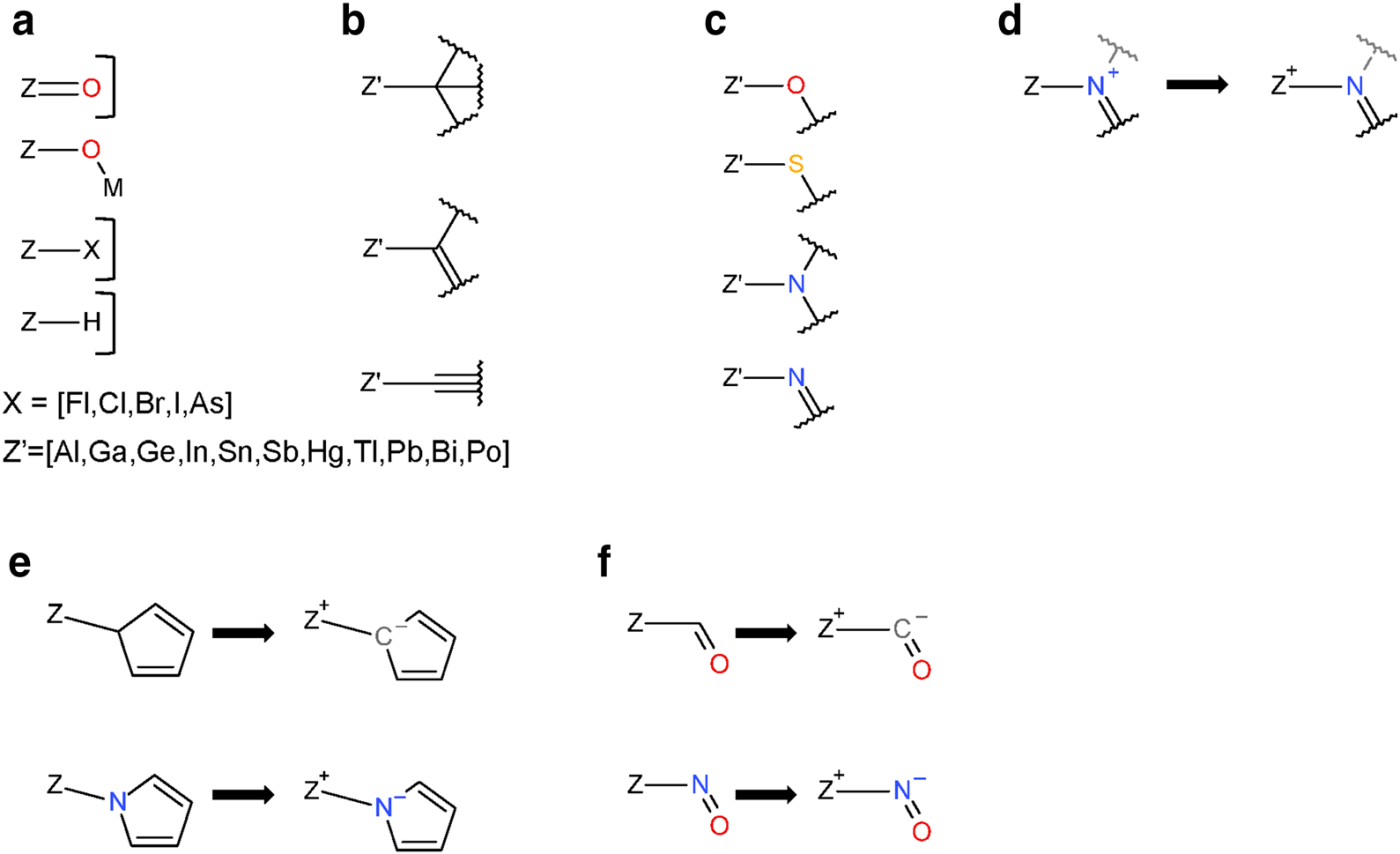
Functional group standardization II. Shown are cases that will not be modified (**a**–**c**) and pre-processing steps carried out before the covalent single bond is replaced by a complex bond. Z indicates the transition metals and semiconductors (see Fig. 28). Z′ as used in **b** and **c** is a subset of the elements in Z. Terminal atoms are specified as such by visually restraining them using a parenthesis ‘]’. The transformation index for transition metal processing (**d**–**f**) is 15 (see the text for the description of transformation indices). **a** Bonds that are not modified (true for all elements in Z): double bond to oxygen, single bond to oxygen that is single-bonded to a metal M (see Fig. 28b), single bond to a halogen X, single bond to hydrogen. **b** Bonds that are not modified for elements in Z’: single bond to tetra-valent carbon. **c** Bonds that are not modified for elements in Z’: single bond to di-valent oxygen, single bond to di-valent sulfur, single-bond to tri-valent nitrogen. **d** A positive charge is moved from tetra-valent nitrogen to the transition metal. **e** Special case of carbon and nitrogen in carbon-only and nitrogen-containing five-membered aromatic rings, respectively. The same transformation applies to 7-membered aromatic carbon-only rings. **f** Special case of carbon and nitrogen double-bonded to oxygen

**Fig. 32:**
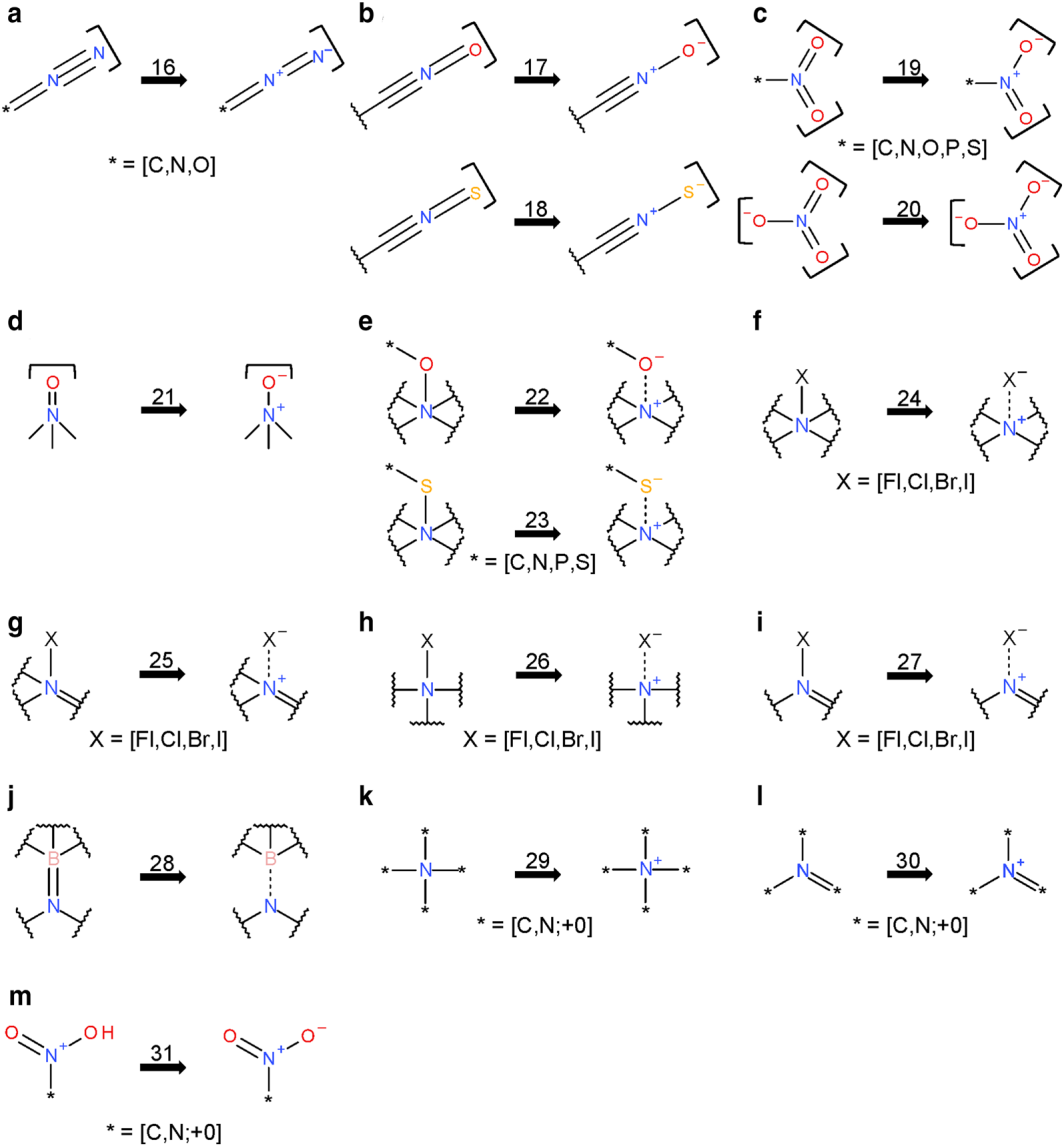
Functional Group Standardization III. If not mentioned otherwise, hydrogen atoms are as depicted, and wildcard asterisks (*), representing connected any atoms, can be hydrogen atoms. Connected carbon atoms are shown without labels and should not be confused with ‘any’ connections. Parenthesis indicates terminal atom. Numbers above arrows are transformation indices for respective standardization rules (see the text for the description of transformation indices). **a** Penta-valent nitrogen connected to terminal nitrogen (triple bond) and carbon, nitrogen or oxygen (double bond). **b** Penta-valent nitrogen connected to terminal oxygen or sulfur (double bond) and non-terminal carbon (triple bond). **c** Nitro group and nitrate (penta-valent representation). **d** Single-bonded atoms adjacent to nitrogen are (not necessarily terminal) carbon. **e** Covalent single bond between penta-valent nitrogen and oxygen or sulfur replaced by ionic bond. **f**, **g** Covalent single bond between penta-valent nitrogen and halogen replaced by ionic bond. **h**, **i** Covalent single bond between tetra-valent nitrogen and halogen replaced by ionic bond. **j** Double bond between tetra-valent nitrogen and boron replaced by dative bond. **k**, **l** Nitrogen without implicit hydrogens. **m** Nitro group (tetra-valent representation)

**Fig. 33:**
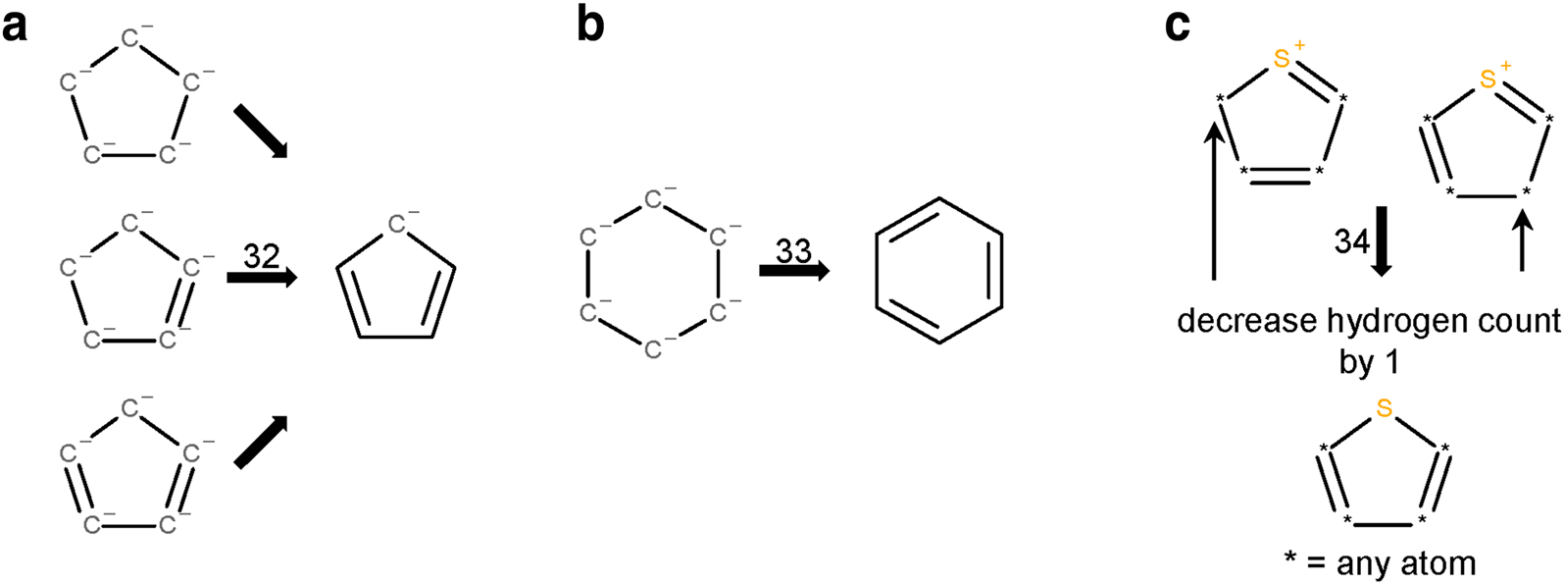
Functional group standardization IV. Numbers above arrows are transformation indices for respective standardization rules (see the text for the description of transformation indices). **a** Different representations of cyclopentadiene used in metallocene structures are unified. **b** Case for benzene analogous to **a**. **c** Standardization of thiophene derivatives. Transformation is only successful if implicit hydrogen counts are sufficient

To normalize functional group representation, the structure is checked against a set of substructures (displayed in Figs. 30, 31, 32, and 33). If they are in a “common” known, non-standard configuration, they are standardized to a preferred representation. Each of these “standardization” rules displayed in these Figures is designated with an integer called a “transformation index”, which is displayed above the arrow. It is in this step that the non-standard bonds (ionic, complex, and dative bonds) are defined.

#### Oxides and analogous cases for carbon

The first group of standardization rules handles the standardization of various oxides and analogous cases for carbon (Fig. 30a–d). Hydrogen and charge preferences are set and valences are adjusted. Many zwitter-ionic bonds are converted to double bonds (which in some cases is an overly aggressive normalization that prevents some known forms of stereochemistry).

#### Ionic bonds

Ionic bonds are set to indicate interactions between charged atoms as appropriate. Nonetheless, the involved atoms keep their charges (Fig. 30e–g). If ionic bonding partners are not connected by a bond, an ionic bond is defined (Fig. 30e). A prerequisite is that the two ionic bonding partners are the only matches for their respective type. If, for example, two Na^+^ ions and one Cl^−^ ion are present, it can’t be decided which one of the Na^+^ is involved in the bond and no ionic bond is set. If the ionic bonding partners are connected by a covalent (single) bond, this bond is replaced by an ionic bond and charges are adapted as necessary (Fig. 30f, g). The conversion of a covalent into an ionic bond also applies to the charged variants of this scenario. The alterations in charge are incremental in this case and not hard coded as + 1/− 1 and + 2/− 2, respectively (this is an area where more aggressive normalization than currently performed may be warranted, given the combinatoric ways of drawing various equivalent salt forms).

#### Tri-valent oxygen

The standardization of tri-valent oxygen handles cases where the coordinate bond between oxygen and boron is represented as covalent single bond (Fig. 30h). In those cases, the bond is replaced with a dative bond. The oxygen and boron atoms must be uncharged prior to this modification.

Three different cases exist for the standardization of tri-valent oxygen (Fig. 30i). The atom must be uncharged and terminal, connected only by a triple bond to another atom. If such an atom is connected to a carbon atom that is connected to a metal by a single bond or a terminal uncharged carbon atom (as in carbon monoxide), a charge of − 1 is placed on the carbon atom and the oxygen gets assigned charge + 1. In all other cases, the oxygen atom gets assigned charge + 1.

#### Transition metals and semiconductor elements

The simplest case for the processing of transition metals and semiconductor elements is when this atom is not connected to other atoms. If it has a charge present in the valence list (provided in Additional file 1), its processing terminates successfully. Otherwise the charge is set to 0 (there are varying approaches to transition metal charge schemes employed, often with the transition metal charge being used to ensure a net neutral molecule as opposed to a known valid formal charge, making it difficult or near impossible to reliably understand what was the original chemist intent from the structure alone). In both cases, standardization proceeds with the next transition metal atom if there is one. If the transition metal atom is connected to other atoms, certain bonding scenarios remain unmodified (Fig. 31a–c). In other cases, covalent bonds will be replaced by complex bonds and the participating atoms’ charges and/or hydrogen counts will be adapted (Fig. 31d–e).

The unmodified bonding scenarios are presented in Fig. 31a–c: a double-bond to terminal oxygen, single bond to oxygen that is connected to a metal atom, single bond to a terminal halogen or hydrogen. Furthermore, a subset of semiconductors and transition metals (Al, Ga, Ge, In, Sn, Sb, Hg, Tl, Pb, Bi, Po) can have covalent (single) bonds to tetra-valent carbon, di-valent oxygen and tri-valent nitrogen. In all those cases, no modifications to atom configurations and bonds are applied. All other adjacent atoms are processed as follows:

- If the transition metal atom is connected to the adjacent atom by anything else other than a single bond or if the other atom does not belong to any of the organic, semiconductor, and metal element classes, and is not boron, silicone, or selenium, it remains unchanged and standardization proceeds with the next neighboring atom.
- If the neighboring atom is a positively charged nitrogen atom that engages in a pi bond, + 1 is added to that of the transition metal atom and that of the nitrogen atom is set to 0 (Fig. 31d).
- For standardization to proceed, the configuration of the connected atom must be in the valence list. If the adjacent atom is uncharged carbon in an aromatic 5- or 7-membered carbon-only ring or uncharged nitrogen in an aromatic 5-membered nitrogen-containing ring, its charge is set to − 1 and that of the transition metal atom is increased by + 1 (as illustrated in Fig. 31e). This accounts, for example, for situations encountered in porphyrin systems.
- The same happens if the adjacent atom is uncharged carbon or nitrogen (both not in a ring) that is connected to an oxygen atom by a double bond: The adjacent atom gets assigned charge − 1 and that of the transition metal is incremented by + 1 (Fig. 31f). Uncharged carbon, uncharged nitrogen and uncharged sulfur (except for the case of tetra-valent sulfur with one hydrogen atom) get assigned a negative charge as well, and the charge of the transition metal is incremented by 1. In the mentioned special case of sulfur, the hydrogen atom is removed. In the case the neighboring atom is a nitrogen with charge + 1, its charge is set to 0. If the charge of the neighboring atom has not been changed by any of those rules, the number of implicit hydrogens on the adjacent atom is incremented by 1.
- Finally, the covalent single bond between the transition metal atom and its neighbor is replaced by a complex bond.

After all adjacent atoms are processed this way, if the collective changes resulted in a configuration of the transition metal atom that is not in the valence list, charge alterations to this transition metal atom are undone by either setting it to its original charge or, if that is also not in the valence list, to 0. If the changes to the connected atoms resulted in invalid configurations, this will be detected in the next standardization step.

#### Penta-valent nitrogen

Seven cases of penta-valent nitrogen are differentiated (Fig. 32a–g). If a penta-valent nitrogen is connected to a terminal nitrogen atom by a triple bond and to another carbon, nitrogen, or oxygen atom by a double bond (e.g., the azide functional group), the triple bond is decreased to a double bond by charge separation; the terminal nitrogen gets assigned a charge of − 1 and the former penta-valent one gets a charge of + 1 (Fig. 32a). If the penta-valent nitrogen is connected to a terminal oxygen or sulfur by a double bond as well as a tetra-valent carbon by a triple bond, the double bond is decreased to a single bond by charge separation; the terminal oxygen or sulfur gets assigned a charge of − 1 and the former penta-valent nitrogen gets a charge of + 1 (Fig. 32b). The nitro group as well as nitrate have their own standardized form with charge separated single bonds (Fig. 32c). If a N=O group is attached to a penta-valent nitrogen connected to three carbon atoms by single bonds, the double bond to nitrogen is replaced by a single bond, placing a positive charge on the nitrogen and a negative charge on the terminal oxygen (Fig. 32d). If one of the adjacent atoms to a penta-valent nitrogen with five single-bonded connections in total is oxygen (or sulfur) that is single-bonded to C, N, P or S, the N–O (or N–S) bond is replaced by an ionic bond, placing a positive charge on the nitrogen and a negative charge on the oxygen (or sulfur) (Fig. 32e). The same processing is applied if a halogen (F, Cl, Br, I) atom is connected to a penta-valent nitrogen with five single-bonded connections (Fig. 32f) or with three single-bonded and one double-bonded connections (Fig. 32g).

#### Tetra-valent nitrogen

Subsequently to penta-valent nitrogen, tetra-valent cases are processed. As a simple rule, if a tetra-valent nitrogen has a zero charge and at least one implicit hydrogen, the charge is considered the more reliable information and the implicit hydrogen count is decreased by 1. Otherwise, the charge on the nitrogen is increased by 1. The additional cases are like those for penta-valent nitrogen. If the nitrogen with four connections (all single-bonded, Fig. 32h) or three connections (two single-bonded and one double-bonded, Fig. 32i) is single-bonded to a halogen, the nitrogen-halogen single bond is replaced by an ionic bond, placing a positive charge on nitrogen and a negative charge on the halogen (Fig. 32h, i). If a tetra-valent nitrogen is connected to a penta-valent boron atom by a double bond, this bond is replaced by a dative bond (Fig. 32j). An uncharged tetra-valent nitrogen atom explicitly connected to carbon or nitrogen atoms by four single bonds (Fig. 32k) or by two single bonds and one double bond (Fig. 32l) gets assigned a charge of + 1. If a nitro group is represented with a charged tetra-valent nitrogen and a single-bonded hydroxyl group (thus could not be fixed using rules for penta-valent nitrogen), the hydroxyl group is deprotonated (Fig. 32m).

#### Ring systems

The last set of standardization rules for functional groups consider select ring systems. One of them is the cyclopentadienyl ring in metallocenes, which is represented as a five-membered ring with negative charges on all carbon atoms and varying bond representations (Fig. 33a). Those representations are unified to a cyclopenta-1,3-diene with a single negative charge on the 5-position. Analogous to this case, a cyclohexane with a negative charge on all carbon atoms is standardized to benzene (Fig. 33b). Finally, a broad spectrum of possible thiophene derivatives is brought to a standardized form with double bonds in 2- and 4-positions (Fig. 33c). Substituents are not further specified in any of the three ring systems, accounting for a variety of molecular contexts.

#### Verify valence

**Fig. 34:**
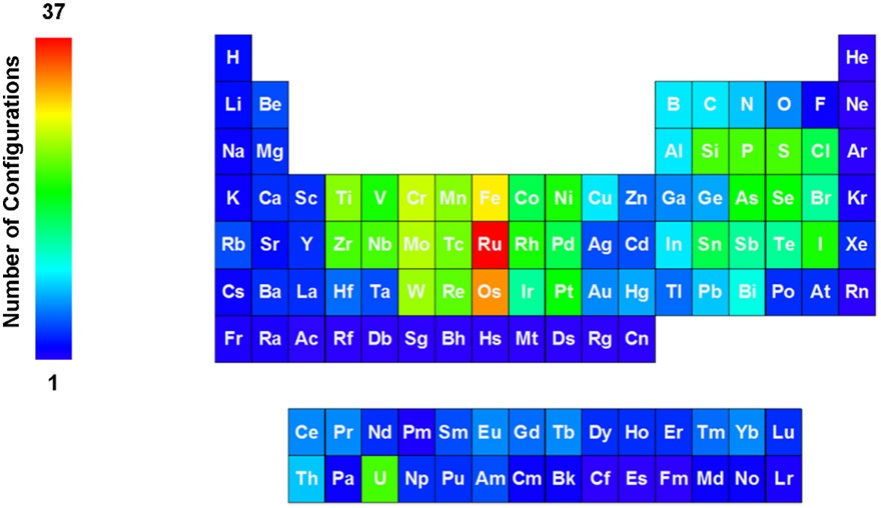
Valence list statistics. This heatmap illustrates the number of configurations per element in the valence database that are valid. For every element, the valence database contains configurations describing valid combinations of the charge, number of sigma bonds, number of pi bonds and number of implicit hydrogen atoms. All combinations are supplied as supporting information in Additional file 1

To verify the valence of an atom, it is compared to an extensive list of allowed configurations for each element type regarding formal charge, the number of σ bonds, the number of π bonds and the maximum allowed number of implicit hydrogen atoms. In total, 981 allowed configurations exist. The distribution of rules amongst elements is shown in Fig. 34. The full valence list is provided as supporting information in Additional file 1.

#### Standardize annotations

PubChem stores bond annotations as properties. These are used to control customized bond visualization, for example, for PubChem-specific non-standard bond types. These annotations can be provided by PubChem data contributors during substance submission. They are converted to covalent bonds during pre-processing and re-perceived. To prevent them from influencing subsequent steps, they are removed at this point during standardization processing.

#### Standardize valence bond form

This step generates a canonical preferred tautomer of a structure, considering protons and charges as mobile elements. For this purpose, the various covalently-connected components of a deposited substance are treated separately and a canonical tautomer is generated for each one of them. If a component has less than two connected atoms, its processing is skipped. Before the actual valence bond canonicalization, the structure is checked against a hand-curated ‘blacklist’ of structures that spent too much time in this step in the past without yielding a better tautomer (vide infra). If the component is on this blacklist (65 structures, provided as canonical SMILES in Additional file 2), it skips valence bond canonicalization. The component is checked against a second list of structures subject to limited processing (1746 structures provided in Additional file 3). The maximum number of generated tautomers per connected component is 250,000 in the unlimited case. In the limited case, this number is reduced to 2500 to reduce processing time at the expense of a less-extensive canonic walk through valence-bond forms.

Explicit hydrogen atoms are made implicit with the same exceptions as described in *Verify Hydrogen*. Certain charges are identified in the component that should not be modified during the valence bond canonicalization (for example, these are charged atoms in annotated complex or ionic bonds, terminal N^−^ as in [N^−^] = [N^+^]=*, and the N^+^ and O^−^ as in a nitro group). These are immobilized on the respective atoms; later, generated tautomers that do not possess the identical pattern of those charges are rejected. This is the case for charged atoms involved in complex bonds (possibly) generated in a previous step, and negative charges around certain nitrogen configurations: (1) if a positively charged and tetra-valent nitrogen with an explicit degree of two is connected to a terminal negatively charged nitrogen by a double bond, the negative charge on the terminal nitrogen is kept in place (e.g., azide group); (2) if a positively charged and tetra-valent nitrogen with an explicit degree of three is connected to a terminal oxygen (or sulfur) atom with charge − 1 and another oxygen (or sulfur) atom by a double bond, the negative charge on the terminal oxygen (or sulfur) atom is kept in place (e.g., nitro group). During the optimization, tautomerization of methyl and methylene groups is not considered, due to an extensive expansion of memory and computational cost. (Improved normalization covering acidic hydrogen atoms on carbon is warranted but not performed, as there are many cases of sp2-hybridized carbon atoms that could also be readily represented in an sp3-hybridized form, especially in keto-enol cases. In some cases, the opposite is true, especially for some heterocycles where the presence of sp3-hybridized carbon prevents aromaticity from being identified.)

Tautomers for each component are enumerated using the function *OEEnumerateTautomers* in the OpenEye Quacpac toolkit [90]. The maximum acceptable energetic category of generated tautomers is defined based on charges present in the component. This value controls which atom types can be generated during the tautomer enumeration. Based on a classification scheme, no tautomer will be generated that has a less preferred class than the original structure. Those classes are from least preferred to most preferred:

1. negatively and positively charged carbon atoms both are present in the structure;
2. a combination of negatively charged nitrogen, oxygen, sulfur, phosphorus or carbon and positively charged nitrogen, oxygen, sulfur, phosphorus or carbon are present in the structure;
3. the structure has any number of charged carbon atoms (positive or negative);
4. at least one positively charged oxygen atom is present in the structure;
5. at least one negatively charged nitrogen atom is present in the structure;
6. at least one positively charged nitrogen or negatively charged oxygen atom is present in the structure;
7. any other case.

The acceptance/rejection of each newly proposed tautomer is based on a greedy selection according to a scoring function based on simple counts and logic. If the immobilized charges are not identical, the new tautomer is rejected. Otherwise, the new tautomer is preferred over another if it has a lower number of less preferred atom valences. This value is generated for a structure based on atom contributions by subtracting the sum of the actual atom valence and the absolute value of its charge from a preferred valence state. Those preferred states are four for carbon, three for nitrogen and phosphorus, and two for oxygen and sulfur. If two tautomers are equal in this criterion, the one with fewer charged atoms is preferred. If they have the same number of charged atoms, the atom type of the charged atoms is considered. For the following list of criteria, a tautomer is preferred if it is ‘better’ in one of them, if and only if the earlier value is the same. If they are equal, the next score is used for prioritization. Those criteria are:

1. fewer positively charged carbon atoms;
2. fewer negatively charged carbon atoms;
3. fewer negatively charged phosphorus atoms;
4. fewer positively charged sulfur atoms;
5. fewer positively charged oxygen atom;
6. fewer negatively charged nitrogen atoms;
7. more positively charged nitrogen atoms;
8. more negatively charged oxygen atoms;
9. more negatively charged sulfur atoms;
10. more positively charges phosphorus atoms;
11. more zwitter-ionic cases of [N^+^]–[O^−^] with a tetra-valent nitrogen or [N^−^]=[N^+^]=*;
12. more hydrogen atoms on carbon;
13. fewer hydrogen atoms on oxygen;
14. fewer hydrogen atoms on sulfur;
15. more aromatic atoms (here, the *OEAroModelMDL* is used because it has stronger emphasis on cyclic systems and ignores exocyclic bonds, which is preferred in this case);
16. fewer hydrogen atoms on nitrogen;
17. fewer hydrogen atoms on phosphorus;
18. fewer hydrogen atoms on atoms in rings;
19. fewer C=C double bonds.

The best tautomer generated during the enumeration is compared to the original structure using the same prioritization criteria as described above. If the best identified tautomer is not preferred over the original structure, a combined evaluation of the number of C=C and N=N double bonds is performed to account for the (empirically determined) preference of the described method for N=N over C=N. An evaluation score is calculated for each tautomer as [count(C=C) − 2 × count(N=N)]. The structure with the lower score is preferred. If the best out of the enumerated tautomers is still not preferred over the original structure, no changes are made. Otherwise, the identified best tautomer is the preliminary result of this standardization step.

The generated structure (with possibly multiple connected components) is subjected to a valence check as described in *Verify Valence*. If the generation of a canonical tautomer yielded at least one atom with a configuration not in the valence list, the substance fails this standardization step, and consequently standardization. In addition to that, a sanity check of local atom neighborhoods is performed. If a situation was created where a charged atom is adjacent to an atom with the identical charge type, the structure fails this standardization step.

If the processing time for one of the components was above 5 min and the iteration limit was 250,000, the structures is flagged as a candidate to be put on the list for limited tautomer enumeration. If the limit already was set to 2500, and 5 min elapsed in this standardization step, it is flagged as a candidate to be put on the blacklist (such lists are periodically updated in source code).

#### Standardize aromaticity

**Fig. 35:**
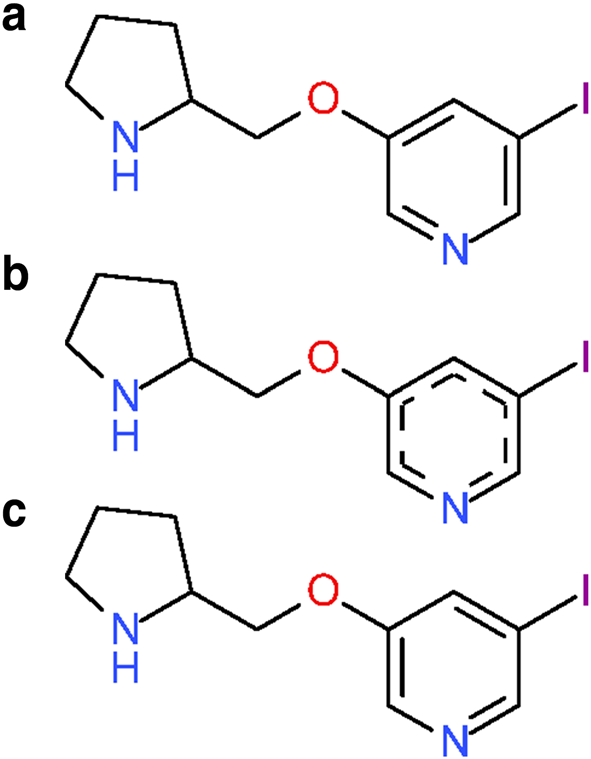
Standardization of Kekulé structure and aromaticity annotation. SID 7 is used as example, without annotation of stereochemistry or isotopic information for clarity. **a** Deposited structure of SID 7. **b** Intermediate representation of the same structure with bond order information in the aromatic system deleted. All bonds are represented as aromatic bonds. This structure is submitted to the OEKekulize function of the OpenEye OEChem C++ toolkit that generates a Kekulé form with single and double bonds instead of aromatic bonds. **c** Result of the aromaticity standardization. The detection of aromaticity, deletion of bond orders and Kekulization resulted in a different Kekulé structure

This step serves two purposes: it normalizes the Kekulé form and also validates roundtrips through the employed aromaticity model in the OpenEye OEChem C++ toolkit [89]. Consequently, it is omitted for structures with less than 3 atoms. First, all existing aromaticity annotation is removed from the structure using the function *OEClearAromaticFlags*. Then, aromaticity is perceived and annotated based on the model *OEAroModelOpenEye* using the function *OEAssignAromaticFlags* with a maximum path length of aromatic cycles of 40 and the prune parameter set to false, preventing rings with exo-double bonds from being annotated as being non-aromatic. The newly assigned aromaticity annotation is used to set the integer bond type of aromatic bonds to a value of 5 (within the OEChem toolkit [89], for non-aromatic bonds, the integer bond types equals the bond order, a value of 5 indicates an aromatic bond), virtually eradicating the present assignment of single and double bonds in the ‘aromatic’ substructures. Atoms and bonds are brought into canonical order by invoking the functions *OECanonicalOrderAtoms* and *OECanonicalOrderBonds*. The modified structure with aromatic bonds is then subjected to the *OEKekulize* function which generates a Kekulé form of the aromatic systems (based on integer bond types). The process is outlined in Fig. 35. Aromaticity annotation as defined by this model is part of the structure specification in the ASN.1 files of standardized entries provided by PubChem but all ‘aromatic’ bonds are represented as being either single or double bonds based on the canonical Kekulé form. In this way, the standardized structure with the generated pattern of single and double bonds is not ambiguous.

#### Standardize stereochemistry

**Fig. 36:**
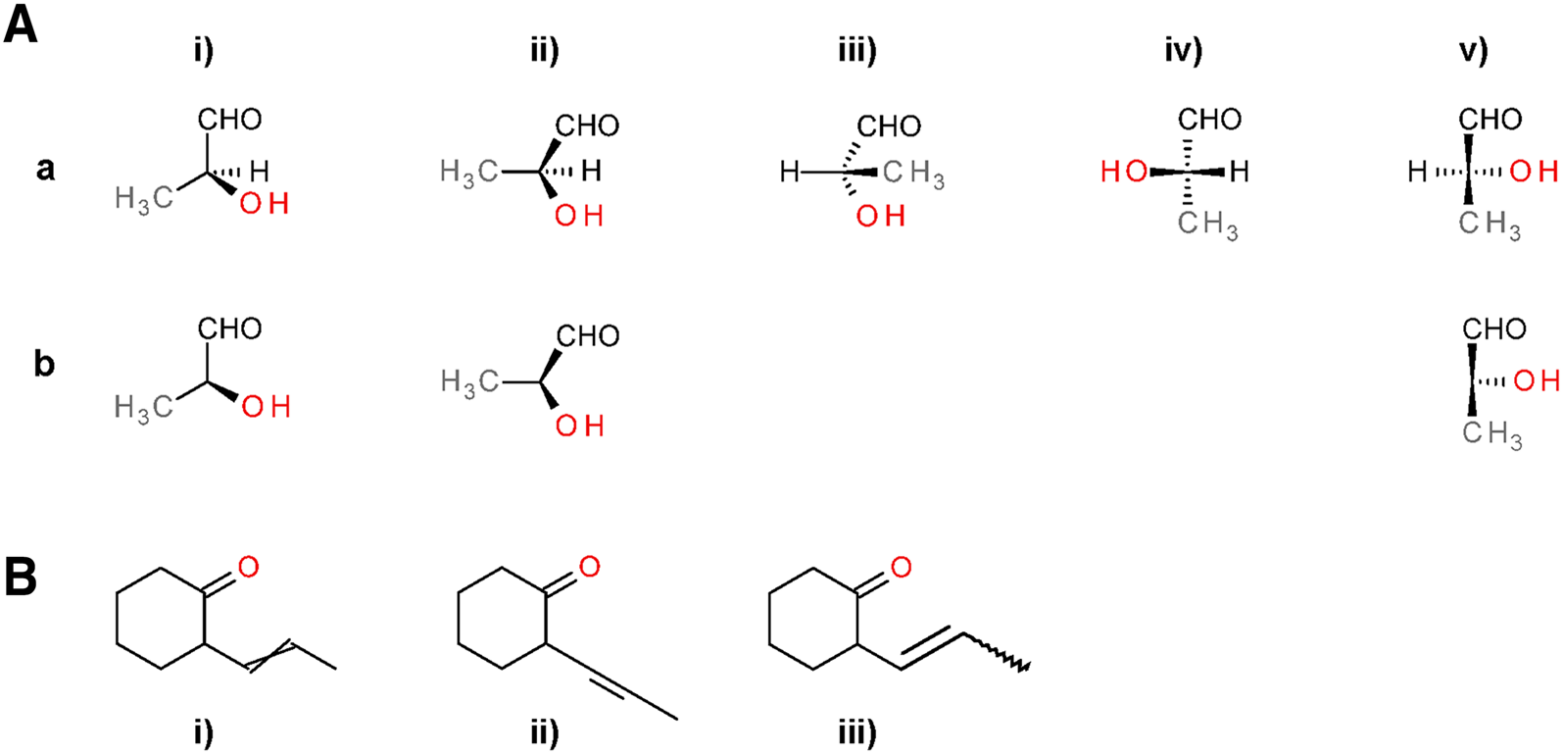
Stereoconfiguration examples. **A** Variants of identical tetrahedral configuration of the same chiral center. **a** Representations with explicit hydrogen atom using: (i) two bonds in, one behind and one in front of the drawing plane (favored representation); (ii) one bond in, two in front of and one behind the drawing plane; (iii) one bond in, one bond in front of and two bonds behind the drawing plane; (iv and v) two bonds in front of and two bond behind the drawing plane. **b** Equivalent representations of the same configuration with implicit hydrogen atom if possible. **B** Representation of a double bond with unknown *cis/trans* configuration as represented in PubChem (i) and corresponding accepted IUPAC recommendations (ii–iii)

This standardization step aims at determining a canonical representation of the configuration of stereocenters: atoms and double bonds with substituents such that interchanging any pair of substituents leads to a different stereoisomer. Previous standardization steps possibly altered the molecule by cleaving or setting new bonds and alternating bond orders. It is possible for such an operation to generate a new stereocenter. In that case, it will be marked as ‘undefined’, because the deposited data could not account for this case. Structures are also tested for the presence of conflicting annotation of stereochemistry. For chiral atoms, for example, their stereo configuration can be annotated as atom property (‘parity’), indicating the direction of travel (clockwise or counter-clockwise) following Cahn–Ingold–Prelog priorities [107] when the substituent with lowest priority is behind the drawing plane. Alternatively, the bonds incident to the chiral atom can be annotated as ‘behind the drawing plane’ (hashed wedge bond), ‘in front of the drawing plane’ (bold wedge bond), or ‘in the drawing plane’. Using bond annotations, several valid representations for the same stereo configuration of an atom exist (see Fig. 36a). Even if both annotations describe an identical configuration of the chiral atom, they can contradict the configuration indicated by 3-D atom coordinates. For double bonds, parity is defined by the substituents on either end with the highest priority. Possible configurations are *E* (position on opposite sides of the double bond), or *Z* (positioned on the same side of the double bond). Again, the configuration specified by atom coordinates can contradict the parity information of the double bond. Here, a complicating factor is that molecule sketching programs [108] can generate arbitrary configurations by automated layout routines. In PubChem, if the *E/Z* configuration of a double bond cannot be resolved, it is configured as ‘undefined’ and represented as a ‘crossed’ double bond. This ‘crossed bond’ representation is chosen due to its simplicity, although it is “not considered acceptable for general use” [101] by IUPAC (Fig. 36b).

#### Stereo configuration of tetrahedral atoms

As a measure of priority, the PubChem standardization protocols employ symmetry classes as implemented in the OpenEye OEChem toolkit [89]. This concept is similar to atom classes in Morgan’s relaxation algorithm [109, 110]. Stereocenters can be easily identified using this concept. If a tetrahedral atom has four adjacent atoms that belong to different symmetry groups, it is chiral. If atoms incident to a stereogenic double bond have adjacent atoms of unequal symmetry groups, that double bond is a stereogenic center. We assign symmetry classes using the function *OEPerceiveSymmetry* in the OpenEye OEChem C++ toolkit [89]. Explicit hydrogen atoms get assigned their own symmetry class of ‘0’ (lowest priority).

In the PubChem structure standardization protocols, stereochemistry standardization relies mostly on routines from the OpenEye OEChem C++ toolkit [89]. If 3-D structural information is provided, stereo information is perceived using the function *OE3DToInternalStereo*. It configures the tetrahedral chirality around atomic centers and the *E/Z* configuration around double bonds based on 3-D atom coordinates, provided they are not set to ‘any’ stereo. If the structure has no atomic coordinates at all (e.g., it was submitted as SMILES string), 2-D coordinates for the structure are generated using the function *OEDepictCoordinates* in the OpenEye OEDepict C++ toolkit that assigns a set of 2-D coordinates to each explicit atom [91]. If tetrahedral atoms in this structure have a defined parity but incident bonds are not annotated as bold or hashed wedges, the parity is used to set this annotation accordingly using the function *OEMDLPerceiveBondStereo*. In all cases of atom-coordinate dimensionality, if tetrahedral atoms are defined only by provided bond annotations (wedge and hashed bonds) the parity is set using the function *OEMDLStereoFromBondStereo*.

Each atom is investigated for its tetrahedral stereochemistry. Atoms are excluded from this step if they are considered aromatic from the earlier aromaticity perception standardization, or have more than one adjacent (or implicit) hydrogen atom, or they are hydrogen atoms, or, in the case of nitrogen, have any adjacent hydrogen atoms. More specific atom tests follow:

- Phosphorus atoms that are not tri-valent and tri-coordinated or penta-valent and tetra-coordinated are non-chiral. The same is true if more than one adjacent atom is of type OH, O^−^, =O, SH, S^−^ or = S, as those may be subject to mesomeric effects (cases of S=P–OH and O=P–SH can be chiral, whereas O=P–OH and S=P–SH cases are achiral).
- Sulfur atoms that are hexa-valent, tetra-coordinated and adjacent via a single bond to carbon with implicit hydrogen atoms or charge, or are incident to a bond that is not a single or a double bond, are non-chiral; the same is true in tetra-valent and tertiary cases if more than one adjacent atom is of type OH, O^−^, =O, SH, S^−^ or = S.
- If an atom is not phosphorous or sulfur, it must be tetra-valent and tetra-coordinated to be considered for chirality tests. Otherwise, it is non-chiral.

If the four adjacent atoms have different symmetry classes, the atom parity is determined. If the parity is not already annotated (*GetStereo* function of the atom returns parity ‘undefined’, indicating the functions invoked earlier failed at parity determination), incident bonds are investigated for wedge-annotation. If they don’t have any, the parity of the chiral atom remains ‘undefined’. Otherwise, stereo configuration is determined based on atom coordinates. If the atom is determined to have parity clockwise or counterclockwise, the reliability of this information is investigated: In the case that the structure does not have 3-D atom coordinates and wedge bond annotation supporting the identified parity is missing, it is annotated as ‘undefined’. Otherwise (if the structure has 3-D information or parity and bond annotation agree), the tetrahedral parity is set as the identified value.

#### Stereo configuration of double bonds

Double bonds considered to exhibit geometric stereoisomerism are non-aromatic double bonds with a connectivity of three for each incident atom. If the bond is in a ring, the smallest ring it is in must be at least of size eight (atoms). If either side has two adjacent (or implicit) hydrogen atoms, it is configured as ‘undefined’. If one of the atoms incident to the double bond is nitrogen, this atom must meet two conditions for further investigating stereochemistry. It is not allowed to have an adjacent atom that is: (1) a hydrogen atom (or an implicit hydrogen atom); or (2) a carbon atom that is adjacent to carbon, hydrogen (or has implicit hydrogen atoms) or incident to a single bond (except for that to the nitrogen atom). Otherwise the double bond is configured as ‘undefined’ (note that structures that do not meet the two conditions may be subject to mesomeric effects).

If the above-mentioned conditions are met, the atoms adjacent to those incident to the double bond are investigated for their symmetry classes. There must be atoms of two different symmetry classes on each side of the double bond, taking implicit hydrogen atoms into account. The bond parity is defined as *E* or *Z* with respect to the pair of adjacent atoms with the highest symmetry class on each side of the double bond. The bond in question is checked for an annotated parity by passing those two atoms to the *GetStereo* function of the double bond. If no bond parity was defined (*GetStereo* returned ‘undefined’) and the atom coordinates were not automatically generated in a prior step, the atom coordinates are used to determine the *E/Z* configuration. If the two defining atoms are on the same (opposite) side of the double bond, it is defined as *Z* (*E*). The IUPAC recommendation for undefined stereochemistry around a double bond is to draw the single bond as extension of the double bond in question, with an angle of 180° between the two. This guideline is implemented with a tolerance of 10°; higher deviations result in the automated perception as *E* or *Z* from atom coordinates. In the case the bond was originally annotated as ‘undefined’, this information has higher priority as the determined parity and the bond remains annotated as undefined (accounting for cases where the 2-D coordinates were only chosen for visualization, not for bond stereo configuration).

#### Standardize explicit hydrogens

All standardized structures in PubChem Compound are available in SDF as well as PubChem-specific ASN.1 or XML format, with explicitly specified hydrogen atoms. So far, the described standardization worked on structures with implicit hydrogen atom counts. In this last step of the standardization, those counts are converted to explicit hydrogen atoms, connected by a single bond to the parent atom.

Only atoms with one or more attached hydrogens are processed in this step, consistent with the definition of an implicit hydrogen count of 0 on all other atoms in the step *Verify Hydrogen*. On each processed atom, the implicit hydrogen counts are set using the function *OEAssignMDLHydrogens* in the OpenEye OEChem C++ Toolkit [89]. The underlying model assumes that the atomic number and formal charge are set to their correct values, which was taken care of in the previous standardization steps. In the case of radicals, hydrogen counts are lower by the number of unpaired valence electrons. The correct position of explicit hydrogen atoms is not determined in this step. This is taken care of separately in the generation of 2-D or 3-D coordinates. The resulting structure must have the count of atoms or bonds not to exceed 999, the upper limit of what is supported by the MDL V2000 MOL file format. Otherwise it fails this standardization step. While not a technical limit of PubChem, this cutoff was a convenient choice to place a limit on what is considered a ‘small’ molecule, and may be changed in the future.

### Unique identifier mapping

The final mapping from substances to entries in PubChem Compound is made based on CACTVS structural hash codes calculated for the standardized structures [111–113]. If the hash code of a standardized structure is not present in Compound, a new entry with a new compound identifier (CID) is created. If a CID with an identical hash code already exists, the substance identifier (SID) of the substance the standardized structure was generated from is associated with this CID and listed as related substance.

### Standardization modification tracking

For this study, we generated a canonical isomeric SMILES (canonical SMILES with stereo information) before and after each step of the standardization procedure using the function *OECreateIsoSmiString* in the OpenEye OEChem C++ toolkit [89]. This way it is possible to detect structural modifications in every step. Isomeric SMILES were generated from de-aromatized structures: prior to string generation, all perceived and annotated aromaticity flags were removed using the function *OEClearAromaticFlags* in the OpenEye OEChem C++ Toolkit [89].

An alternative structure representation for this purpose would have been the IUPAC International Chemical Identifier (InChI) [11–13]. Yet, it does not have an advantage over SMILES in this use case. During the generation of standard InChIs, an InChI-specific structure normalization is performed that would obfuscate modifications resulting from PubChem standardization. InChIs can be configured to be ‘non-standard’ and describe a structure ‘as-is’, essentially making them equivalent to SMILES for our purposes. In this case, there would have been no benefit in choosing InChI and may have created confusion. We also chose SMILES so we could resort to functionalities readily available within the OpenEye Scientific Software Inc. C++ toolkits [89–92], avoiding unnecessary conversion between toolkits or other changes that might alter subsequent analysis.

It is important to note that non-standard bonds used by PubChem are ignored when computing a SMILES. This will make some structures appear to be identical that are not if their nonstandard bonding is different or when compared to structures devoid of such bonds.

### Standardization time statistics

We monitored elapsed standardization per step and total standardization time per substance using the CStopWatch class in the NCBI C++ toolkit [114]. Time was measured as wall time on a mix-use heterogeneous compute cluster. It may not accurately provide actual time spent in cases when a server is overloaded or when using different servers with different processor speeds. With that said, it does give a relative speed on modern hardware.

### Unique structure analysis

The purpose of the described PubChem standardization protocols is the identification of erroneous structures and the compensation for various aspects of chemical structures that lead to multiple valid representations of effectively the same molecular species. Consequently, the number of unique structures in a before/after comparison is expected to be less than the number of processed structures. To determine this degree of structure merging, we compared the numbers of unique structures before and after standardization using their representation as de-aromatized canonical isomeric SMILES. This approach has high structural sensitivity, as it allows distinguishing between stereoisomers as well as different Kekulé structures. Comparison was limited to structures that could be successfully standardized using the PubChem standardization protocols.

### Comparison to InChI structure normalization

Structure normalization is an integral part of the generation of the IUPAC International Chemical Identifier (InChI) [11–13]. The PubChem standardization approach described here was developed independently of InChI and prior to the wide-spread use of InChI. As a first step in the comparison of PubChem standardization and the InChI normalization we compared the numbers of unique structures after standardization identified by their de-aromatized canonical SMILES to those of unique standard InChIs generated from the original structures. For this purpose, standard InChIs were generated using the InChI VC++ projects provided by the InChI Trust [115]. The comparison was limited to the 104,669,789 substances that have complete, non-auto-generated structures. We kept track of differences in standardization/normalization success for both methods. For further analysis, the generated InChIs were converted back to structures (InChI-derived structure) and represented by de-aromatized canonical isomeric SMILES as well. InChI was never designed to be a file format and is not recommended. However, it seemed important to check whether an InChI normalized structure followed by conversion back to a chemical structure would yield the same PubChem-standardized structure to identify caveats/issues.

### Dataset

Results and statistics presented in this study were generated from a local copy of the PubChem Substance ASN.1 files available from the PubChem FTP repository [116], accessed on January 14th 2013. At that time, PubChem contained 116,641,122 substance records with a maximum substance identifier (SID) 144,075,000. The PubChem structure standardization service is accessible as a public resource under https://pubchem.ncbi.nlm.nih.gov/standardize/, and via programmatic interfaces [117].

## Additional files

**Additional file 1.** Valence list. List of valid valences and configurations of atoms with respect to atomic number (column 1), charge (column 2), number of π bonds (column 3), number of σ bonds (column 4) and the maximum number of implicit hydrogens (column 5).

**Additional file 2.** Valence Bond Canonicalization Blacklist. List of 65 structures as SMILES that will not be processed during valence bond canonicalization.

**Additional file 3.** Valence Bond Canonicalization Limitlist. List of 1746 structures as SMILES that are subject to a limited processing during valence bond canonicalization (2500 instead of 250,000 enumerated tautomers).

**Additional file 4.** Substances that failed PubChem standardization but succeed InChI normalization. List of 375,397 substances that failed PubChem standardization but whose InChI strings were successfully generated.

**Additional file 5.** Substances that failed the PubChem valence check but succeeded InChI generation. List of 364,946 substances that failed the PubChem valence check but whose InChI strings were successfully generated.

## References

[1] Brown FK, James AB (1998). Chapter 35—chemoinformatics: what is it and how does it impact drug discovery. Annual reports in medicinal chemistry.

[2] Hann M, Green R (1999). Chemoinformatics—a new name for an old problem?. Curr Opin Chem Biol.

[3] Gasteiger J (2006). Chemoinformatics: a new field with a long tradition. Anal Bioanal Chem.

[4] Engel T (2006). Basic overview of chemoinformatics. J Chem Inf Model.

[5] Varnek A, Baskin II (2011). Chemoinformatics as a theoretical chemistry discipline. Mol Inform.

[6] Vogt M, Bajorath J (2012). Chemoinformatics: a view of the field and current trends in method development. Bioorg Med Chem.

[7] Brecher J (2008). Graphical representation standards for chemical structure diagrams. Pure Appl Chem.

[8] Food and Drug Administration Substance Registration System Standard Operation Procedure Substance Definition Manual. https://www.fda.gov/downloads/ForIndustry/DataStandards/SubstanceRegistrationSystem-UniqueIngredientIdentifierUNII/ucm127743.pdf. Accessed 13 Aug 2016

[9] Weininger D (1988). Smiles, a chemical language and information-system. 1. Introduction to methodology and encoding rules. J Chem Inf Comput Sci.

[10] Weininger D, Weininger A, Weininger JL (1989). Smiles. 2. Algorithm for generation of unique smiles notation. J Chem Inf Comput Sci.

[11] McNaught A (2006). The IUPAC international chemical identifier: InChI—a new standard for molecular informatics. Chem Int.

[12] Heller SR, McNaught AD (2009). The IUPAC international chemical identifier. Chem Int.

[13] Stein SE, Heller SR, Tchekhovskoi DV, Pletnev IV IUPAC International Chemical Identifier (InChI), InChI version 1, software version 1.04 (2011), Technical Manual http://www.inchi-trust.org/fileadmin/user_upload/software/inchi-v1.04/InChI_TechMan.pdf. Accessed 13 Aug 2016

[14] Ash S, Cline MA, Homer RW, Hurst T, Smith GB (1997). SYBYL line notation (SLN): a versatile language for chemical structure representation. J Chem Inf Comput Sci.

[15] Homer RW, Swanson J, Jilek RJ, Hurst T, Clark RD (2008). SYBYL line notation (SLN): a single notation to represent chemical structures, queries, reactions, and virtual libraries. J Chem Inf Model.

[16] Gakh AA, Burnett MN (2001). Modular chemical descriptor language (MCDL): composition, connectivity, and supplementary modules. J Chem Inf Comput Sci.

[17] Gakh AA, Burnett MN, Trepalin SV, Yarkov AV (2011). Modular chemical descriptor language (MCDL): stereochemical modules. J Cheminform.

[18] Panico R, Powell WH, Richter JC (1993). A guide to IUPAC nomenclature of organic compounds recommendations 1993.

[19] Favre HA, Hellwich K-H, Moss GP, Powell WH, Traynham JG (1999). Corrections to a guide to IUPAC nomenclature of organic compounds (IUPAC recommendations 1993). Pure Appl Chem.

[20] Leigh GJ, Favre HA, Metanomski WV (1998). Principles of organic nomenclature.

[21] Dalby A, Nourse JG, Hounshell WD, Gushurst AKI, Grier DL, Leland BA, Laufer J (1992). Description of several chemical-structure file formats used by computer-programs developed at molecular design limited. J Chem Inf Comput Sci.

[22] Accelrys CTFile Formats. http://accelrys.com/products/informatics/cheminformatics/ctfile-formats/no-fee.php. Accessed 13 Aug 2016

[23] TRIPOS Mol2 File Format. http://tripos.com/data/support/mol2.pdf

[24] Warr WA (2011). Representation of chemical structures. Wiley Interdiscip Rev Comput Mol Sci.

[25] Urbaczek S, Kolodzik A, Fischer JR, Lippert T, Heuser S, Groth I, Schuz-Gasch T, Rarey M (2011). NAOMI: on the almost trivial task of reading molecules from different file formats. J Chem Inf Model.

[26] Akhondi SA, Kors JA, Muresan S (2012). Consistency of systematic chemical identifiers within and between small-molecule databases. J Cheminform.

[27] Meng EC, Lewis RA (1991). Determination of molecular topology and atomic hybridization states from heavy-atom coordinates. J Comput Chem.

[28] Baber JC, Hodgkin EE (1992). Automatic assignment of chemical connectivity to organic-molecules in the Cambridge structural database. J Chem Inf Comput Sci.

[29] Hendlich M, Rippmann F, Barnickel G (1997). BALI: automatic assignment of bond and atom types for protein ligands in the Brookhaven Protein Databank. J Chem Inf Comput Sci.

[30] Urbaczek S, Kolodzik A, Groth I, Heuser S, Rarey M (2013). Reading PDB: perception of molecules from 3D atomic coordinates. J Chem Inf Model.

[31] Young D, Martin T, Venkatapathy R, Harten P (2008). Are the chemical structures in your QSAR correct?. QSAR Comb Sci.

[32] Sayle RA (2010). So you think you understand tautomerism?. J Comput Aided Mol Des.

[33] Katritzky AR, Hall CD, El-Dien B, El-Gendy M, Draghici B (2010). Tautomerism in drug discovery. J Comput Aided Mol Des.

[34] Ferrari E, Saladini M, Pignedoli F, Spagnolo F, Benassi R (2011). Solvent effect on keto-enol tautomerism in a new beta-diketone: a comparison between experimental data and different theoretical approaches. New J Chem.

[35] Balabin RM (2009). Tautomeric equilibrium and hydrogen shifts in tetrazole and triazoles: focal-point analysis and ab initio limit. J Chem Phys.

[36] Elguero J, Marzin C, Katritzky AR, Linda P (1976). The tautomerism of heterocycles. Advances in heterocyclic chemistry.

[37] Scior T, Bender A, Tresadern G, Medina-Franco JL, Martinez-Mayorga K, Langer T, Cuanalo-Contreras K, Agrafiotis DK (2012). Recognizing pitfalls in virtual screening: a critical review. J Chem Inf Model.

[38] Sitzmann M, Ihlenfeldt WD, Nicklaus MC (2010). Tautomerism in large databases. J Comput Aided Mol Des.

[39] Pospisil P, Ballmer P, Scapozza L, Folkers G (2003). Tautomerism in computer-aided drug design. J Recept Signal Transduct Res.

[40] Oellien F, Cramer J, Beyer C, Ihlenfeldt WD, Selzer PM (2006). The impact of tautomer forms on pharmacophore-based virtual screening. J Chem Inf Model.

[41] Todorov NP, Monthoux PH, Alberts IL (2006). The influence of variations of ligand protonation and tautomerism on protein-ligand recognition and binding energy landscape. J Chem Inf Model.

[42] Kalliokoski T, Salo HS, Lahtela-Kakkonen M, Poso A (2009). The effect of ligand-based tautomer and protomer prediction on structure-based virtual screening. J Chem Inf Model.

[43] Muchmore SW, Debe DA, Metz JT, Brown SP, Martin YC, Hajduk PJ (2008). Application of belief theory to similarity data fusion for use in analog searching and lead hopping. J Chem Inf Model.

[44] Duarte HA, Carvalho S, Paniago EB, Simas AM (1999). Importance of tautomers in the chemical behavior of tetracyclines. J Pharm Sci.

[45] Jang YH, Goddard WA, Noyes KT, Sowers LC, Hwang S, Chung DS (2002). First principles calculations of the tautomers and pK(a) values of 8-oxoguanine: implications for mutagenicity and repair. Chem Res Toxicol.

[46] Hastings J, Magka D, Batchelor C, Duan L, Stevens R, Ennis M, Steinbeck C (2012). Structure-based classification and ontology in chemistry. J Cheminform.

[47] Bobach C, Bohme T, Laube U, Puschel A, Weber L (2012). Automated compound classification using a chemical ontology. J Cheminform.

[48] Trepalin SV, Skorenko AV, Balakin KV, Nasonov AF, Lang SA, Ivashchenko AA, Savchuk NP (2003). Advanced exact structure searching in large databases of chemical compounds. J Chem Inf Comput Sci.

[49] Martin YC (2009). Let’s not forget tautomers. J Comput Aided Mol Des.

[50] Milletti F, Storchi L, Sforna G, Cross S, Cruciani G (2009). Tautomer enumeration and stability prediction for virtual screening on large chemical databases. J Chem Inf Model.

[51] Greenwood JR, Calkins D, Sullivan AP, Shelley JC (2010). Towards the comprehensive, rapid, and accurate prediction of the favorable tautomeric states of drug-like molecules in aqueous solution. J Comput Aided Mol Des.

[52] Urbaczek S, Kolodzik A, Rarey M (2014). The valence state combination model: a generic framework for handling tautomers and protonation states. J Chem Inf Model.

[53] Gobbi A, Lee ML (2012). Handling of tautomerism and stereochemistry in compound registration. J Chem Inf Model.

[54] Warr WA (2010). Tautomerism in chemical information management systems. J Comput Aided Mol Des.

[55] Schleyer PV, Jiao HJ (1996). What is aromaticity?. Pure Appl Chem.

[56] Lloyd D (1996). What is aromaticity?. J Chem Inf Comput Sci.

[57] Cyranski MK, Krygowski TM, Katritzky AR, Schleyer PV (2002). To what extent can aromaticity be defined uniquely?. J Org Chem.

[58] Randic M (2003). Aromaticity of polycyclic conjugated hydrocarbons. Chem Rev.

[59] Stanger A (2009). What is… aromaticity: a critique of the concept of aromaticity-can it really be defined?. Chem Commun.

[60] Hückel E (1931). Quantentheoretische Beiträge zum Benzolproblem I. Die Elektronenkonfiguration des Benzols und verwandter Verbindungen. Z Phys.

[61] Hückel E (1932). Quantentheoretische Beiträge zum Benzolproblem II. Quantentheorie der induzierten Polaritäten. Z Phys.

[62] Aromaticity Perception. https://docs.eyesopen.com/toolkits/cpp/oechemtk/aromaticity.html. Accessed 23 July 2018

[63] Kekulé A (1865). Sur la constitution des substances aromatiques. Bull Soc Chim Paris.

[64] Kekulé A (1866). Untersuchungen über aromatische Verbindungen. Justus Liebigs Ann Chem.

[65] Herndon WC (1973). Enumeration of resonance structures. Tetrahedron.

[66] Randic M (1976). Enumeration of the Kekule structures in conjugated hydrocarbons. J Chem Soc Faraday Trans.

[67] Blazic BDJ, Trinajstic N (1982). Computer-aided enumeration and generation of the kekule structures in conjugated hydrocarbons. Comput Chem.

[68] Gutman I, Cyvin SJ (1987). A new method for the enumeration of kekule structures. Chem Phys Lett.

[69] Cai F, Shao HQ, Liu CG, Jiang YS (2005). An alternative strategy for count and storage of Kekule and longer range resonance valence bond structures. J Chem Inf Model.

[70] Rashid Z, Van Lenthe JH (2011). Generation of kekule valence structures and the corresponding valence bond wave function. J Comput Chem.

[71] Kearsley SK (1993). A quick robust method for assigning a kekule structure. Comput Chem.

[72] Hansen P, Zheng ML (1995). Assigning a kekule structure to a conjugated molecule. Comput Chem.

[73] Blessington B (1995). A serious problem with computer-processing of stereochemistry in chemical-structure files—the need for standardization. Chirality.

[74] Martin E, Monge A, Duret JA, Gualandi F, Peitsch MC, Pospisil P (2012). Building an R&D chemical registration system. J Cheminform.

[75] Fourches D, Muratov E, Tropsha A (2010). Trust, but verify: on the importance of chemical structure curation in cheminformatics and QSAR modeling research. J Chem Inf Model.

[76] Clark RD, Waldman M (2012). Lions and tigers and bears, oh my! three barriers to progress in computer-aided molecular design. J Comput Aided Mol Des.

[77] Egorova KS, Toukach PV (2012). Critical analysis of CCSD data quality. J Chem Inf Model.

[78] Oprea T, Olah M, Ostopovici L, Rad R, Mracec M, Ford M, Livingstone D, Dearden J, Waterbeemd H (2003). On the propagation of errors in the QSAR literature. EuroQSAR 2002 designing drugs and crop protectants: processes, problems and solutions.

[79] Olah M, Mracec M, Ostopovici L, Rad R, Bora A, Hadaruga N, Olah I, Banda M, Simon Z, Mracec M, Oprea TI (2005) WOMBAT: world of molecular bioactivity. In: Chemoinformatics in drug discovery. Wiley-VCH Verlag GmbH & Co. KGaA, pp 221–239. 10.1002/3527603743.ch9

[80] Tiikkainen P, Bellis L, Light Y, Franke L (2013). Estimating error rates in bioactivity databases. J Chem Inf Model.

[81] Kim S, Thiessen PA, Bolton EE, Chen J, Fu G, Gindulyte A, Han LY, He JE, He SQ, Shoemaker BA, Wang JY, Yu B, Zhang J, Bryant SH (2016). PubChem substance and compound databases. Nucl Acids Res.

[82] Kim S (2016). Getting the most out of PubChem for virtual screening. Expert Opin Drug Discov.

[83] Wang YL, Bryant SH, Cheng TJ, Wang JY, Gindulyte A, Shoemaker BA, Thiessen PA, He SQ, Zhang J (2017). PubChem BioAssay: 2017 update. Nucl Acids Res.

[84] McEntyre J, Lipman D (2001). PubMed: bridging the information gap. Can Med Assoc J.

[85] PubMed. http://www.ncbi.nlm.nih.gov/pubmed

[86] Bolton EE, Chen J, Kim S, Han LY, He SQ, Shi WY, Simonyan V, Sun Y, Thiessen PA, Wang JY, Yu B, Zhang J, Bryant SH (2011). PubChem3D: a new resource for scientists. J Cheminform.

[87] Bolton EE, Kim S, Bryant SH (2011). PubChem3D: conformer generation. J Cheminform.

[88] Kim S, Bolton EE, Bryant SH (2013). PubChem3D: conformer ensemble accuracy. J Cheminform.

[89] OpenEye OEChem C++ Toolkit, version 1.9.0; OpenEye Scientific Software Inc., Santa Fe, NM. http://www.eyesopen.com/oechem-tk

[90] OpenEye Quacpac C++ Toolkit, version 1.9.0; OpenEye Scientific Software Inc., Santa Fe, NM. http://www.eyesopen.com/quacpac-tk

[91] OpenEye OEDepict C++ Toolkit, version 1.9.0; OpenEye Scientific Software Inc., Santa Fe, NM. http://www.eyesopen.com/oedepict-tk

[92] OpenEye Lexichem C++ Toolkit, version 1.9.0; OpenEye Scientific Software Inc., Santa Fe, NM

[93] Warr WA, Bajorath J (2011). Some trends in chem(o)informatics. Chemoinformatics and computational chemical biology, vol 672. Methods in molecular biology.

[94] Fanton M, Floris M, Cristiani A, Olla S, Medda R, Sabbadin D, Bulfone A, Moro S (2013). MMsDusty: an alternative InChI-based tool to minimize chemical redundancy. Mol Inform.

[95] Rogers FB (1963). Medical subject heading. Bull Med Libr Assoc.

[96] Audi G, Bersillon O, Blachot J, Wapstra AH (2003). The NUBASE evaluation of nuclear and decay properties. Nucl Phys A.

[97] Wiberg N (2007) Natürliche Nuklide. In: Lehrbuch der Anorganischen Chemie, 102. Auflage. De Gruyter, Berlin, p 2001

[98] Ehrlich HC, Rarey M (2012). Systematic benchmark of substructure search in molecular graphs—From Ullmann to VF2. J Cheminform.

[99] O’Boyle NM (2012). Towards a universal SMILES representation—a standard method to generate canonical smiles based on the InChI. J Cheminform.

[100] Clark AM (2011). Accurate specification of molecular structures: the case for zero-order bonds and explicit hydrogen counting. J Chem Inf Model.

[101] Brecher J (2006). Graphical representation of stereochemical configuration—(IUPAC recommendations 2006). Pure Appl Chem.

[102] Razinger M, Balasubramanian K, Perdih M, Munk ME (1993). Stereoisomer generation in computer-enhanced structure elucidation. J Chem Inf Comput Sci.

[103] Perdih M, Razinger M (1994). Stereochemistry and sequence rules—a proposal for modification of Cahn–Ingold–Prelog system. Tetrahedron Asymmetry.

[104] Cieplak T, Wisniewski JL (2001). A new effective algorithm for the unambiguous identification of the stereochemical characteristics of compounds during their registration in databases. Molecules.

[105] Wild DJ (2009). Grand challenges for cheminformatics. J Cheminform.

[106] Schneider G (2010). Virtual screening: an endless staircase?. Nat Rev Drug Discov.

[107] Cahn RS, Ingold C, Prelog V (1966). Specification of molecular chirality. Angew Chem Int Ed Engl.

[108] Ertl P (2010). Molecular structure input on the web. J Cheminform.

[109] Morgan HL (1965). The generation of a unique machine description for chemical structures—a technique developed at chemical abstracts service. J Chem Doc.

[110] Figueras J (1993). Morgan revisited. J Chem Inf Comput Sci.

[111] Ihlenfeldt WD, Takahashi Y, Abe H, Sasaki S (1994). Computation and management of chemical-properties in CACTVS—an extensible networked approach toward modularity and compatibility. J Chem Inf Comput Sci.

[112] Ihlenfeldt WD, Gasteiger J (1994). Hash codes for the identification and classification of molecular-structure elements. J Comput Chem.

[113] CACTVS Chemoinformatics Toolkit version 3.365, Xemistry GmbH, Lahntal, Germany. http://www.xemistry.com

[114] NCBI C++ Toolkit. http://www.ncbi.nlm.nih.gov/IEB/ToolBox/CPP_DOC/

[115] InChI Trust, InChI software version 1.04 for Standard and Non-Standard InChI/InChIKey. http://www.inchi-trust.org/fileadmin/user_upload/software/inchi-v1.04/INCHI-1-API.ZIP

[116] PubChem FTP. ftp://ftp.ncbi.nlm.nih.gov/pubchem/

[117] Kim S, Thiessen PA, Bolton EE, Bryant SH (2015). PUG-SOAP and PUG-REST: web services for programmatic access to chemical information in PubChem. Nucl Acids Res.

